# Exploring minimum dietary diversity among cambodian children using four rounds of demographic and health survey

**DOI:** 10.1038/s41598-024-64714-0

**Published:** 2024-06-26

**Authors:** Anjali Singh, Dil B. Rahut, Tetsushi Sonobe

**Affiliations:** 1 Project Associate (MLE), Project Concern International, New Delhi, Delhi 110020 India; 2 Asian Development Bank Institute, 3-2-5 Kasumigaseki, Chiyoda-ku, Tokyo, 100-6008 Japan

**Keywords:** Dietary diversity, Inadequate minimum dietary diversity, Geographical disparity, Cambodia, Health care economics, Diseases, Nutrition disorders, Malnutrition

## Abstract

Dietary diversity among children is a crucial factor influencing their nutritional status; therefore, this paper uses data from four rounds of the Cambodia Demographic and Health Survey (CDHS) to examine the minimum dietary diversity among children aged 6–23 months. Multilevel binary regression is used to evaluate the variation in minimum dietary diversity at the cluster and province levels. The results show that nearly half of Cambodian children consistently lacked access to vitamin A-rich fruits and vegetables. Although the prevalence of inadequate minimum dietary diversity (MDD) among children significantly dropped from 76% in 2005 to 51% in 2021–2022, it is still high and needs attention. A decomposition analysis (Blinder–Oaxaca decomposition) was further used to understand the drivers of this temporal change in dietary diversity. The empirical results show that clusters represented the most significant source of geographic variation with respect to all eight food groups and MDD. Nutritional policy should improve education and awareness, reduce socio-economic disparities, leverage media, and promote full antenatal care to improve dietary diversity in Cambodia. Initiatives targeting the enhancement of insufficient minimum dietary diversity intake should encompass individual aspects and be customized to suit geographic and community settings.

## Introduction

Nutrition plays a pivotal role in fostering the growth and development of children. A well-balanced diet, which includes a variety of nutrient-rich foods from different food groups, is essential for optimal health and development. Dietary variety is linked to improved energy and nutrient consumption, benefiting both adults and children in both developed and developing nations. The Sustainable Development Goals also emphasize the importance of improving nutrition and food security, particularly for vulnerable populations such as children, as they can help meet and even exceed at least six of the goals by 2030^1^. In 2020, an astonishing 148 million children under age 5 years were estimated to experience stunting (characterized by being shorter than their age suggests), while 45 million were affected by wasting (exhibiting thinness relative to their height), and 37 million were overweight or obese. Approximately 45% of fatalities among children under the age of 5 years are associated with undernutrition, predominantly prevalent in low- and middle-income nations^2^. Paradoxically, the aforementioned countries are experiencing an alarming increase in childhood overweight and obesity rates. These figures underscore the pressing necessity to address malnutrition in this age group, promoting healthy growth and development. Malnutrition refers to the imbalance of nutrients in the body, either due to a lack of essential nutrients or an excess intake of unhealthy foods that combines all three aspects: undernutrition, micronutrient malnutrition, and overweight.

The World Health Organization (WHO) has introduced the Infant and Young Child Feeding Practice (IYCF) indicators to evaluate the nutritional status of children and identify possible interventions. Among these indicators is Minimum Dietary Diversity (MDD), which assesses dietary diversity by considering the number of different food groups consumed within a specific timeframe that can provide a wide range of essential nutrients that are crucial for optimal child growth and development^3^. It is defined as the intake of foods from a minimum of five out of eight food groups, including breast milk, grains, roots and tubers, legumes and nuts, dairy products, flesh meats, and fish, eggs, and fruits and vegetables. The design of MDD serves a threefold purpose of significant importance: Evaluating the adequacy of crucial nutrients in infants’ diets, overseeing and assessing the impact of efforts to enhance feeding practices for infants and young children, and pinpointing particular areas requiring focused interventions and support to enhance child nutrition results^3,4^.

WHO and UNICEF have jointly established a framework emphasizing the significance of MDD in attaining optimal child nutrition and health outcomes within two years of a child’s life^2^. The framework emphasizes the significance of implementing proper Infant and Young Child Feeding (IYCF) practices during this critical two-year window, as they significantly impact a child’s overall health and development. These practices have far-reaching effects on various aspects, including health, growth, learning, and behavior, and ultimately play a pivotal role in shaping adult social relationships, well-being, and earnings. Optimal IYCF practices and minimum dietary diversity are challenging in low-income countries, where access to a variety of nutritious foods may be limited. Global estimates for feeding children aged 6–23 months have indicated that only a quarter of children in developing countries meet the minimum dietary diversity requirements^5^. As the feeding rate is low in many developing countries, the research highlights the urgent need for improvement in this area^5–7^. Research in Nepal revealed that just 47% of children aged 6–23 months fulfilled the MDD criteria by consuming foods from at least four of the seven specified food groups^8^. In India, the magnitude of inadequate MDD ranged from 36 to 77%, while Indonesia reported an MDD of 47%^9,10^.

Data from the Cambodia Demographic Health Survey (CDHS, 2014) disclosed a disconcerting drop of nearly 9 percentage points in the practice of exclusive breastfeeding for babies during their first six months, sliding from 74% in 2010 to 65% in 2014^11^. Adding to this, continued breastfeeding for children aged 12–15 months was also on a descending trend. Moreover, pre-lacteal feeding for newborns rose substantially from 19 to 28%^12^. Such shifts in breastfeeding practices could plausibly contribute to the observed low prevalence of minimum dietary diversity among Cambodian children. It has been observed that dietary diversity is significantly associated with child stunting and mortality. Disturbingly, within the same timeframe, out of 1.7 million children under 5 years of age in Cambodia, around 660,000 (40%) are experiencing stunting. The child mortality rate for those under 5 is 54 per 1000 live births, with nearly 45% of these child deaths attributed to different forms of undernutrition^13^.

Government initiatives have been carried out across Cambodia to meet global nutrition targets. The fast-track roadmap is one of the initiatives taken by the Cambodian government under the National Population Program (NPP) to improve the infant and young child feeding practice for a 1000-day period. Efforts have been consistently made; however, achieving the exclusive breastfeeding target remains elusive, with only 65% of infants aged 0 to 5 months being exclusively breastfed. Progress has been made towards reducing stunting, but the prevalence still remains high at 32.4% among children under 5 years of age, surpassing the regional average for Asia at 21.8%^14^.

Limited evidence exists on the frequency and causes of inadequate dietary consumption among Cambodian children under 2 years. Discrepancies in household socioeconomic status, maternal education, religion, caste, and agricultural productivity have been linked to variations in dietary diversity across regions and countries^5^. However, the diverse regional access and food cultures further complicate the assessment and comparison of dietary patterns nationwide. More research is needed to comprehend the localized differences in MDD within districts and between cluster levels since these geographical units are where other indicators of child malnutrition differ. This study, therefore, examines disparities in MDD—comprising breast milk, grains, legumes, dairy, flesh foods, eggs, vegetables, and other fruits—at the micro-regional level.

Further, this study is robust as it uses four rounds of large datasets covering 9056 children from 2005 to 2022 and a multilevel binary regression model to evaluate how various explanatory variables impact minimum dietary diversity and account for variations at the cluster and province levels. This effort is poised to enhance policymaking by illuminating district-separated dietary diversity among children. This study aims to examine the temporal changes and their drivers, such as socio-economic, demographic, and spatial, in the minimum dietary diversity among Cambodian children from 2005 to 2022.

## Result

### Descriptive statistics

A total of 9056 children aged 6–23 months, living with their mothers, were included in this analysis from four consecutive rounds of Cambodian Household and Demographic Survey (CDHS) data, spanning from 2005 to 2021–2022 [2020 (CHDS 2005); 2372 (CHDS 2010); 2143 (CHDS 2014); and 2321 (CDHS 2021–2022)]. Table 1 shows that the sample across all four rounds exhibited similarities in terms of age distribution, region of residence, wealth status, education, occupation, marital status, and more. More than one-third of the sampled children fell in the age range of 6–11 months, with the majority being males and having second or third-order birth across the surveys. Over three-fourths of these children’s mothers were found to be in the age group of 20–34 years, with the highest percentage of mothers receiving education observed in the fourth round (89%), indicating an increasing trend over time. In contrast, fathers’ education peaked during the third round of the survey.

**Table 1:** Sociodemographic characteristics of the mothers/caregivers and the children in a study of inadequate minimum dietary diversity and associated factors among children aged 6–23 months in Cambodia: based on 2005–2021 DHS.

Sample characteristics	CDHS 2005		CDHS 2010		CDHS 2014		CDHS 2021		Combined	
	%^a^ (N^a^)	%^b^	%^a^ (N^a^)	%^b^	%^a^ (N^a^)	%^b^	%^a^ (N^a^)	%^b^	%^a^ (N^a^)	%^b^
Child age										
6–11 months	34.4 (764)	85.0	34.6 (821)	78.6	34.7 (745)	74.5	34.6 (804)	61.5	34.6 (3135)	74.8
12–17 months	33.2 (737)	70.4	33.8 (804)	62.2	31.4 (673)	52.1	32.5 (756)	45.1	32.8 (2970)	57.6
18–23 months	32.3 (719)	71.4	31.5 (747)	68.8	33.7 (724)	51.2	32.7 (761)	47.0	32.5 (2951)	59.5
Child sex										
Male	50.8 (1130)	73.9	52.0 (1234)	69.3	50.6 (1086)	59.7	51.2 (1190)	51.1	51.2 (4640)	63.5
Female	49.1 (1091)	77.7	47.9 (1138)	70.7	49.3 (1057)	59.5	48.7 (1131)	51.7	48.7 (4416)	64.9
Birth order										
First	28.3 (629)	73.4	36.8 (874)	71.7	39.9 (857)	57.9	34.1 (793)	51.7	34.8 (3153)	63.2
Second or third	40.3 (897)	75.6	44.2 (1050)	68.6	44.1 (946)	59.3	56.0 (1300)	50.0	46.3 (4194)	62.3
Fourth or higher	31.2 (694)	78.1	18.8 (448)	69.6	15.8 (340)	64.5	9.8 (227)	58.2	18.8 (1709)	70.5
Mother’s age										
15–19 yrs	4.41 (98)	82.4	4.83 (115)	79.6	5.13 (110)	71.2	4.32 (100)	53.2	4.67 (423)	71.8
20–34 yrs	74.9 (1663)	75.1	80.8 (1918)	69.1	82.7 (1773)	59.4	75.7 (1759)	51.7	78.5 (7112)	63.8
35–49 yrs	20.6 (459)	76.6	14.3 (340)	71.3	12.1 (260)	56.1	19.9 (462)	49.8	16.8 (1521)	63.8
Mother’s education										
No education	22.4 (499)	80.4	16.8 (399)	78.9	12.6 (270)	71.6	10.9 (255)	61.5	15.7 (1424)	74.9
Primary	59.9 (1330)	76.8	56.2 (1333)	71.6	51.1 (1097)	63.0	40.4 (938)	56.1	51.8 (4699)	68.0
Secondary	17.0 (378)	66.3	25.1 (596)	60.3	32.8 (703)	51.0	41.7 (968)	46.7	29.2 (2646)	53.7
Higher	0.55 (12)	61.7	1.83 (43)	68.2	3.4 (73)	45.7	6.86 (159)	36.2	3.18 (288)	44.5
Mother’s occupation										
Not working	0.64 (14)	91.5	20.9 (497)	69.7	30.8 (660)	60.6	32.3 (751)	54.4	21.2 (1923)	60.7
White collar	18.3 (407)	70.9	18.1 (430)	63.0	19.1 (410)	50.0	26.9 (627)	39.2	20.6 (1874)	53.9
Agri worker	51.7 (1149)	77.3	49.1 (1167)	73	32.8 (703)	67.9	13.6 (317)	59.7	36.8 (3336)	72.2
Service/manual work	29.2 (650)	75.8	11.7 (278)	68.2	17.2 (370)	52.5	26.9 (626)	55.8	21.2 (1923)	63.7
Marital status										
Married	95.7 (2125)	75.8	96.0 (2278)	70.2	96.0 (2058)	59.8	95.3 (2213)	51.1	95.7 (8674)	64.2
Not married	4.3 (95)	74.2	3.97 (94)	64.4	3.97 (85)	55.1	4.65 (108)	57.3	4.22 (383)	62.8
Preceding birth interval										
First birth	28.6 (635)	73.3	37.0 (879)	71.7	40.1 (861)	57.9	34.2 (795)	51.6	35 (3170)	63.2
< 36 months	29.4 (654)	80.3	25.0 (594)	72.2	20.8 (448)	67.9	15.1 (352)	48.1	22.6 (2047)	69.7
≥ 36 months	41.9 (932)	74.2	37.9 (899)	66.8	38.9 (834)	56.9	50.6 (1175)	52.3	42.4 (3840)	62.0
Media exposure										
No	14.6 (324)	87.5	15.5 (370)	75.7	19.3 (415)	73.6	56.5 (1313)	56.2	26.7 (2422)	66.3
Partial	68.4 (1519)	76.1	61 (1447)	70.9	66.0 (1415)	58.9	39.4 (915)	46.8	58.4 (5295)	65.0
Full	17 (377)	64.1	23.4 (555)	63.6	14.6 (313)	44.0	4.03 (94)	29.6	14.7 (1340)	56.8
Wanted pregnancy										
Wanted	70.4 (1564)	74.8	84.4 (2004)	69.7	82.4 (1766)	58.8	82.3 (1911)	51.1	80 (7245)	63.3
Unwanted/mistimed	29.5 (656)	78.0	15.5 (368)	71.2	17.6 (377)	63.2	17.6 (410)	52.7	20.0 (1811)	67.8
ANC Visit during pregnancy										
< 4 visit	71.0 (1577)	77.6	35.7 (847)	73.9	22.9 (492)	67.4	12.3 (288)	57.0	35.3 (3203)	73.2
≥ 4 visit	28.9 (644)	71.3	64.2 (1524)	67.8	77.0 (1651)	57.2	87.6 (2034)	50.6	64.6 (5853)	59.2
Place of delivery										
Health facility-Pub	18.7 (416)	70.3	55.3 (1312)	69.7	71.5 (1532)	59.5	78.2 (1817)	55.1	56.0 (5077)	61.4
Health facility-Pri	5.38 (119)	72.7	10.1 (240)	64.5	16.1 (347)	50.6	18.7 (435)	35.7	12.6 (1142)	50.2
Home/Other	75.9 (1685)	77.3	34.5 (820)	71.9	12.3 (264)	71.9	2.95 (68)	52.3	31.3 (2837)	74.7
Visited health facility in last 12 months										
No	55.7 (1237)	76.1	44.9 (1065)	71	36.2 (777)	60.7	40.1 (932)	55.4	44.2 (4011)	67
Yes	44.2 (983)	75.3	55.1 (1307)	69.0	63.7 (1366)	58.9	59.8 (1390)	48.7	55.7 (5045)	61.9
Father’s education										
No education	15.0 (334)	84	12.0 (287)	77.4	9.48 (203)	63.6	14.0 (326)	61.4	12.7 (1150)	72.4
Primary	49.5 (1099)	78.2	44.2 (1050)	71.3	43.6 (936)	67.4	33.6 (782)	54.4	42.7 (3867)	68.9
Secondary	33.2 (739)	69.4	39.4 (936)	66.0	40.0 (858)	52.9	41.2 (956)	49.6	38.5 (3488)	59.0
Higher	2.17 (48)	60.3	4.19 (99)	70.7	6.81 (146)	43.3	11.1 (258)	36.4	6.09 (551)	46.5
Father’s occupation										
Not working	1.2 (27)	78.2	0.74 (18)	69.9	1.18 (25)	45.6	8.74 (203)	54.6	3.01 (272)	57.1
White collar	11.6 (260)	66.3	14.9 (353)	67.7	14.9 (321)	47.7	19.1 (444)	40.6	15.2 (1377)	54.0
Agri worker	60.3 (1339)	79.1	51.9 (1233)	74.0	46.7 (1002)	65.2	19.8 (461)	53.7	44.5 (4034)	71.2
Service/manual work	26.8 (595)	72.4	32.3 (768)	64.5	37.1 (795)	57.8	52.2 (1214)	53.9	37.2 (3372)	60.5
Place of residence										
Urban	14.2 (316)	67.2	16.2 (385)	71.6	14.0 (300)	43.9	38.0 (883)	39.7	20.8 (1884)	51.5
Rural	85.7 (1904)	77.2	83.7 (1987)	69.6	85.9 (1842)	62.1	61.9 (1439)	58.6	79.2 (7172)	67.5
Wealth index										
Poorest	26.5 (590)	82.8	25.5 (606)	74.2	23.9 (514)	72.2	21.6 (502)	59.7	24.4 (2212)	72.7
Poorer	21.8 (486)	80.6	19.8 (470)	73.3	19.9 (427)	64	18.8 (438)	54.3	20.1 (1820)	68.5
Middle	18.1 (404)	74.4	19.4 (460)	73.1	18.5 (397)	62.5	20.0 (466)	58.5	19.0 (1728)	67.0
Richer	16.5 (367)	67.8	18.4 (437)	60.8	17.8 (382)	51.6	21.0 (488)	49.7	18.4 (1673)	57.0
Richest	16.8 (374)	67.6	16.8 (398)	65.8	19.7 (423)	44.3	18.4 (428)	32.9	17.9 (1623)	52.0
Household size										
≤ 4	32.2 (716)	74.4	32.5 (771)	73.3	31.0 (665)	58.5	36.4 (845)	52.4	33.1 (2997)	64.4
5–9	61.0 (1355)	76.5	60.9 (1444)	67.9	62.4 (1339)	60.4	60.1 (1396)	50.7	61.1 (5534)	63.9
≥ 10	6.73 (149)	75.3	6.58 (156)	72.0	6.49 (139)	56.6	3.45 (80)	52.4	5.79 (525)	65.8
Sex of head of household										
Male	83.4 (1852)	75.6	76.4 (1813)	68.8	77.5 (1661)	59.5	72.1 (1675)	51.5	77.3 (7001)	64.3
Female	16.6 (369)	76.3	23.5 (559)	73.6	22.4 (482)	60.0	27.8 (646)	51.0	22.7 (2055)	63.8

Source: Authors estimation using Demographic and Health Survey (DHS) Data.

N^a^-Weighted frequency of the dataset. %^a^- % of the dataset. %^b^ Prevalence of not meeting minimum dietary diversity.

Additionally, approximately 27% of mothers and 19% of fathers held white-collar jobs in the last round of the survey, reflecting an increasing trend over the survey periods. Across all four survey rounds, less than 5% of mothers were not in a partnership or marriage across all rounds. More than half of them had partial exposure to media until 2014, but this figure dropped to 39% in the latest survey round. Furthermore, the percentage of mothers who had visited a healthcare facility in the past year ranged from 44 to 60% across the four rounds.

More than a third of mothers considered their newborn children as ‘wanted,’ and had attended four or more ANC visits, marking an increase from 2005 to 2021–2022 with a change of 59 points. Over half of the children were born in government health facilities, shooting up from 19% in 2005 to 78% in 2021–2022. Conversely, home births decreased over time, with only 3% of children being born at home in the last survey round. Regarding community-level variables, most of the children were rural inhabitants, belonged to households with the poorest wealth index, had household sizes ranging from 5 to 9 members, and had male household heads.

### Prevalence of inadequate minimum dietary diversity

Table 1 illustrates the trend in the prevalence of inadequate minimum dietary diversity (MDD) across four survey rounds in Cambodia spanning from 2005 to 2021–2022, considering selected socio-economic and demographic factors. As seen in Table 1, children aged 6–11 months, those born as fourth or higher birth order, and females, in comparison to males, exhibited the highest proportion of inadequate MDD. However, the gender gap has decreased over the years. Children born to younger mothers and residing in rural areas had a higher prevalence of inadequate MDD compared to their counterparts.

Furthermore, parental factors such as maternal and paternal education, as well as household wealth index, displayed a consistent and significant association with a decreasing trend in inadequate MDD among children. Across all years, children whose mothers had undergone four or more Antenatal Care (ANC) visits, held white-collar jobs (clerical, managerial, professional, technical), had full exposure to media, and had visited a healthcare facility in the last 12 months consistently exhibited the lower prevalence of inadequate MDD. Likewise, children considered as “wanted,” delivered in private healthcare facilities, and residing in households headed by males had the lowest prevalence of inadequate MDD compared to their counterparts.

**Figure 1:**
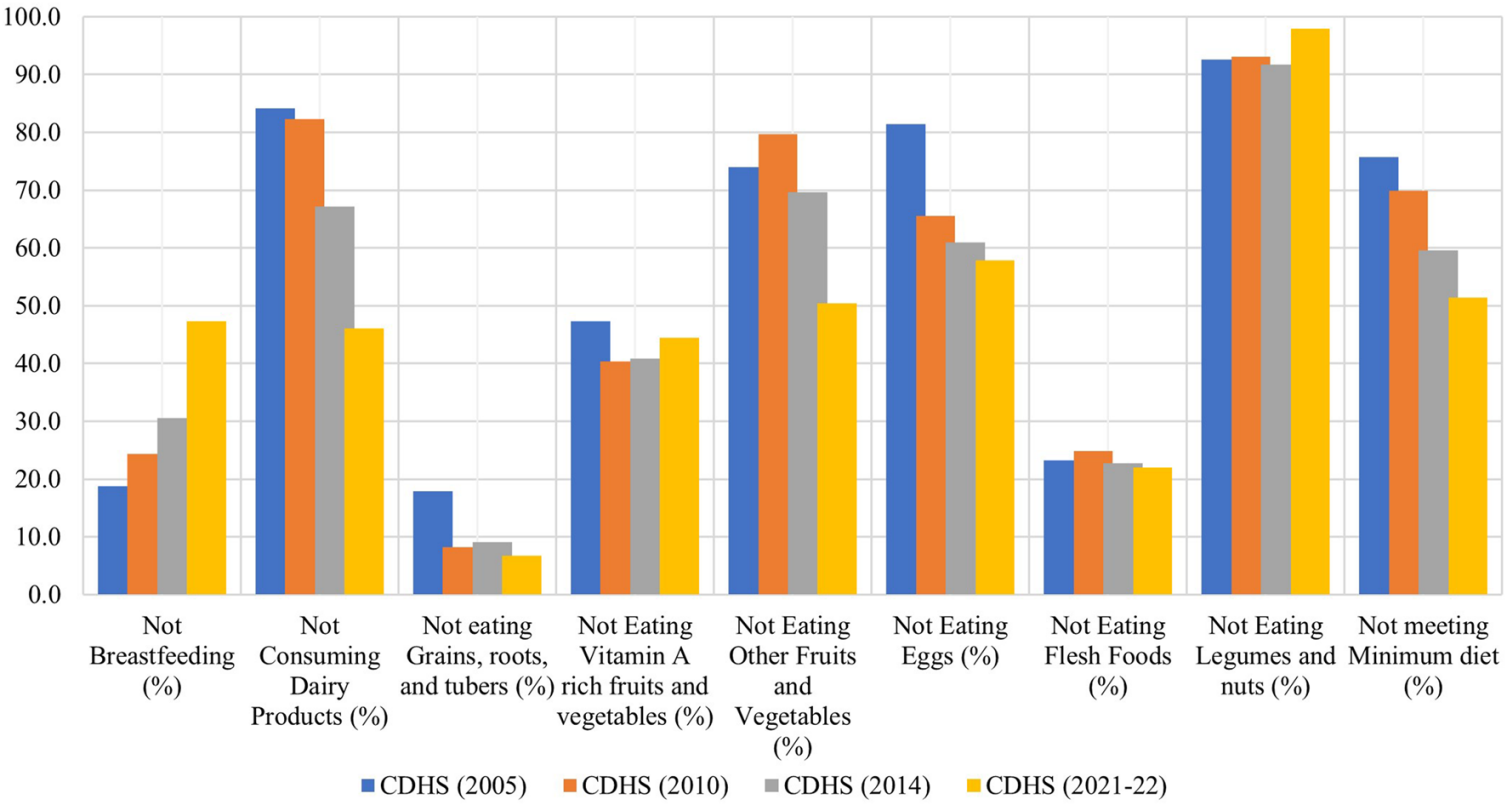
Weighted prevalence of intake of 8 nutrient-rich food groups for children 6–23 months.

Figure 1 illustrates the varying prevalence of each of the eight nutrient-rich food groups included in the minimum dietary diversity metric over the years. Noticeably, there has been an increase in the trend of non-breastfeeding, with a 46% relative change over the last 16 years. Furthermore, legumes and nuts were consistently the least consumed food group in all surveys, with only 2% of sampled children consuming them in the latest survey. Grains, roots, and tubers, on the other hand, remained the most consumed food group in all surveys, with prevalence ranging from 82 to 93% from 2005 to 2021–2022. The consumption of flesh foods remained consistent over the years, with more than three-fourths of sampled children incorporating them into their diet. In contrast, protein sources such as eggs, dairy products, and fruits and vegetables had lower prevalence compared to other food groups, contributing to inadequate MDD. Over three-fourths of the children previously lacked access to these food items. However, in a recent survey, children aged 6–23 months showed improved access to dairy products (54%), fruits and vegetables (50%), and eggs (42%). Nearly half of the Cambodian children consistently lacked access to vitamin A-rich fruits and vegetables. According to data, the prevalence of inadequate MDD among children aged 6–23 months has significantly dropped from 76% in 2005 to 51% in 2021–2022.

**Figure 2:**
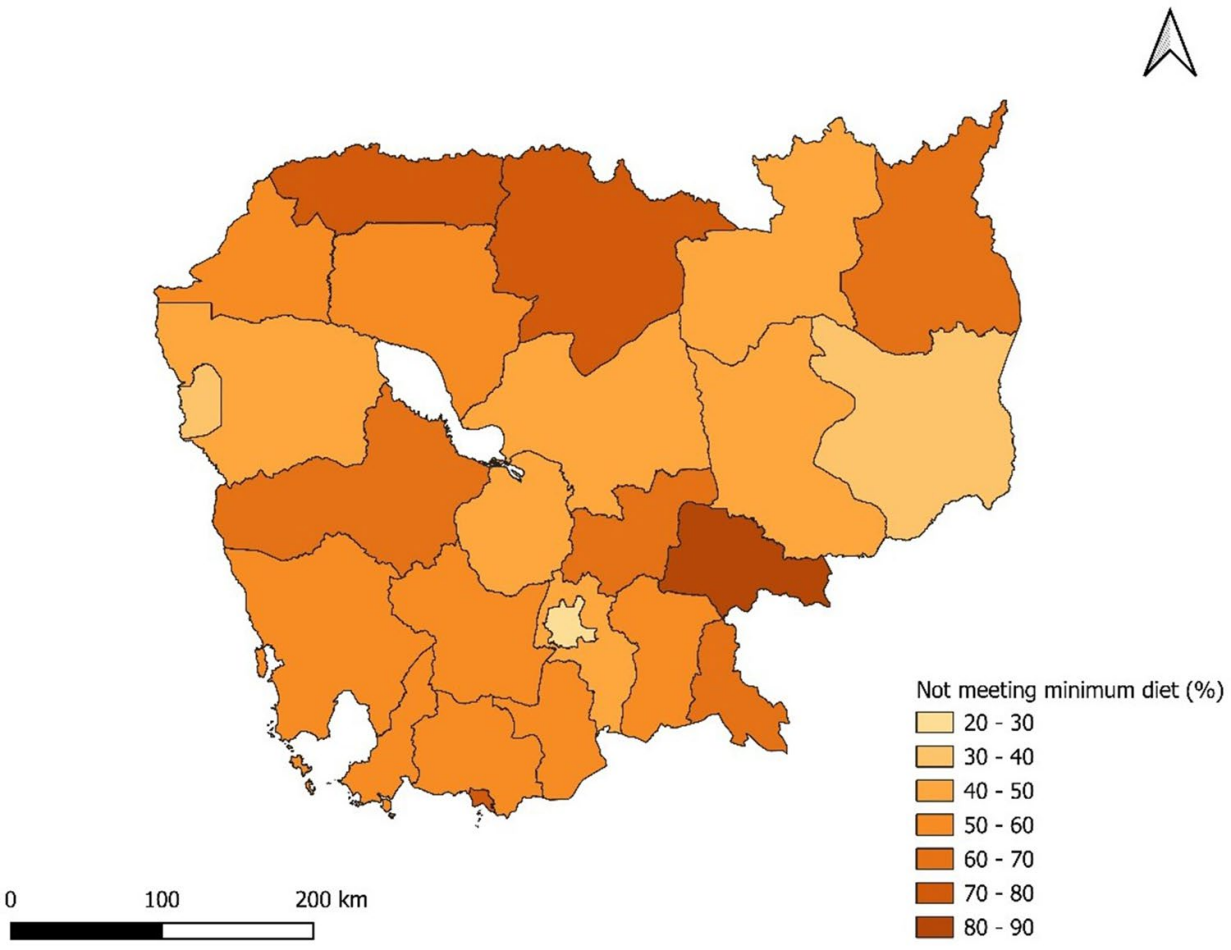
Geographic distribution of percent children not meeting MDD across 19 provinces in Cambodia, 2021–2022.

**Figure 3:**
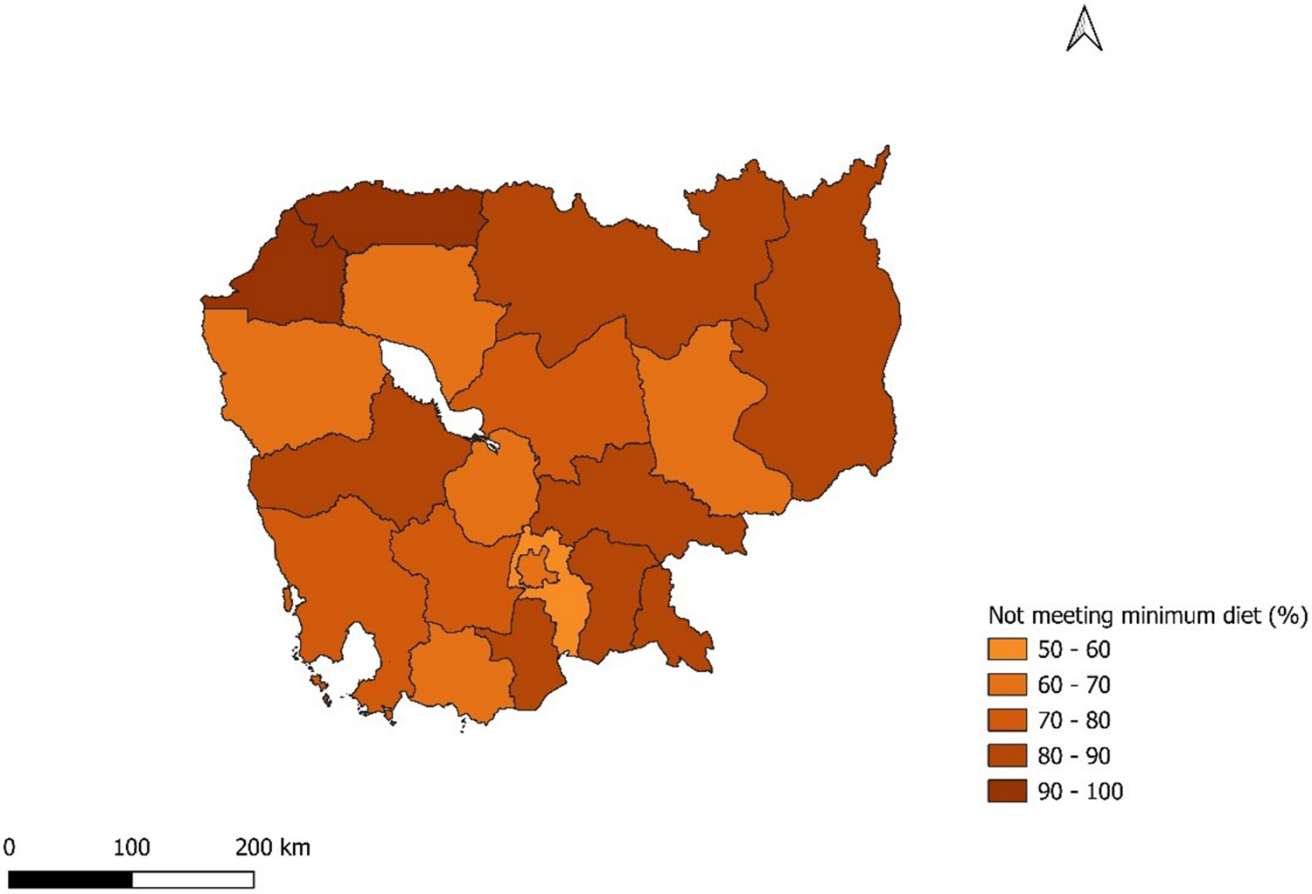
Geographic distribution of percent children not meeting MDD across 19 provinces in Cambodia, 2005.

**Figure 4:**
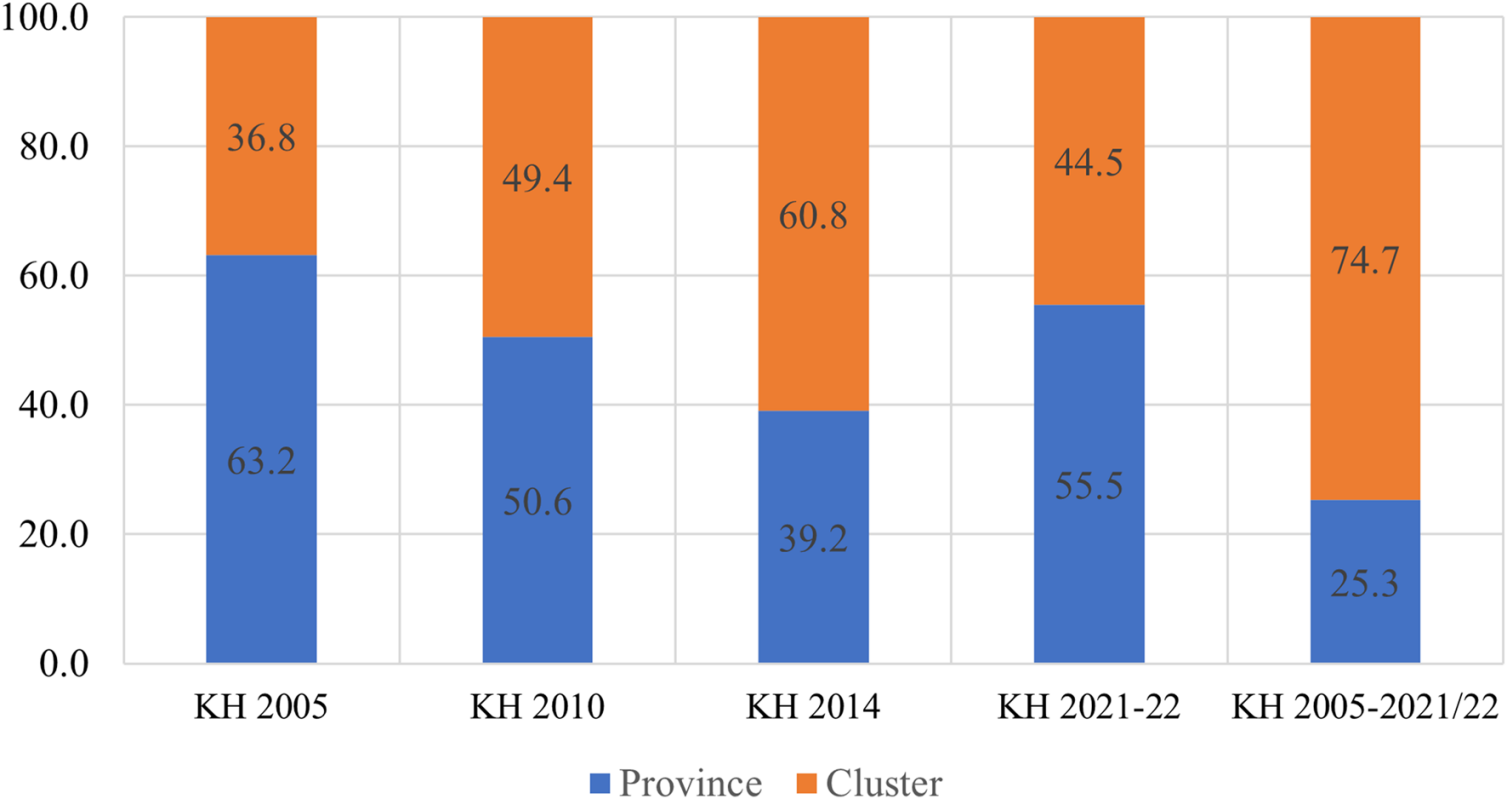
Proportion of variance partitioned between clusters, and provinces for not meeting minimum dietary diversity.

**Figure 5:**
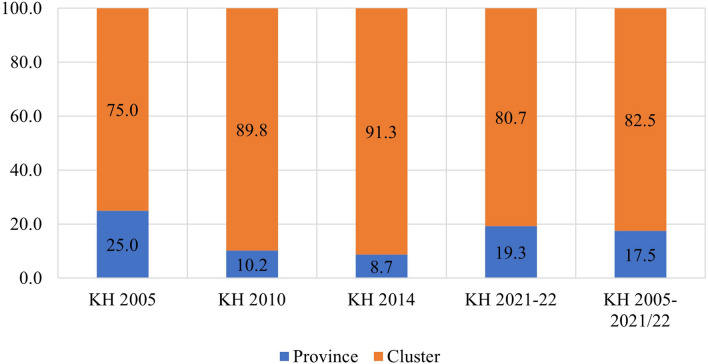
Proportion of Variance partitioned between clusters, and provinces for inadequate breastfeeding.

**Figure 6:**
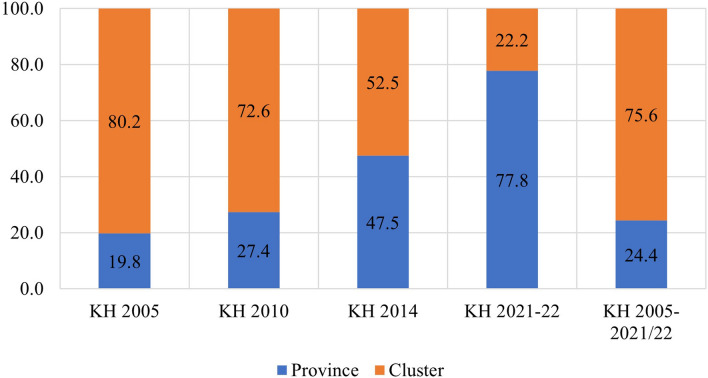
Proportion of Variance partitioned between clusters, and province for consumption of Dairy Products.

**Figure 7:**
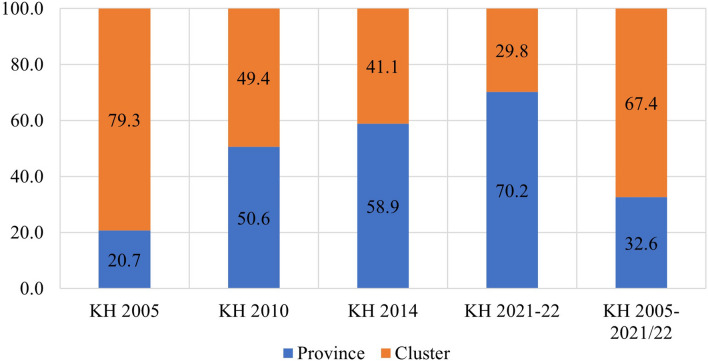
Proportion of variance partitioned between clusters and province for consumption of Grains, roots, and tubers.

**Figure 8:**
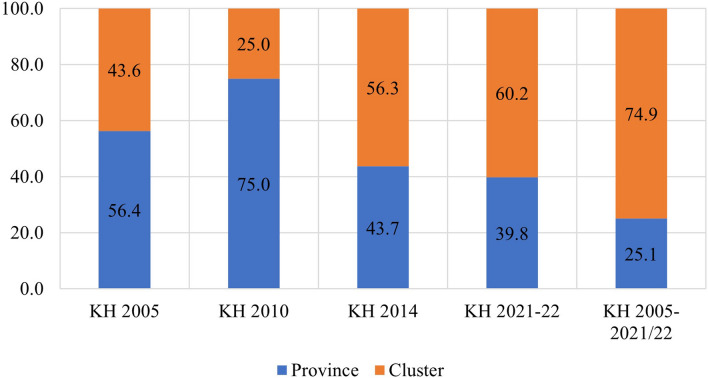
Proportion of variance partitioned between clusters, districts, and provinces for inadequate consumption of Vitamin A rich fruits and vegetables.

Before analyzing the role of socio-economic and demographic factors on minimum dietary diversity, it is essential to discuss the prevalence of inadequate MDD across Cambodia’s provinces, as illustrated in Figs. 2 and 3. In 2005, all provinces had over half of the children (6–23 months) with access to four or fewer diverse nutrient-rich food items. Notably, in 10 out of 19 provinces listed, over 80% of children had inadequate MDD. However, a positive shift is evident in the recent survey where 9 out of 25 provinces saw less than a 50% prevalence in inadequate MDD. Comparisons show a downward trend in all provinces. Phnom Penh led this decline, decreasing by 47 points, and registered the lowest inadequate MDD at 23% in 2021–2022. In contrast, Tboung Khmum reported the highest occurrence, with only 17% of children having access to a minimum of five food groups from the eight nutrient groups in 2021–2022. The regional prevalence over the years for inadequate consumption of each of the eight food groups and the variables used in the empirical analysis is also provided in Supplementary file (Appendix 1, Figures 1–8), which confirms improvement in the minimum dietary diversity and food category but with huge variation at the provincial level.

### Drivers of inadequate dietary diversity

After identifying the factors that influence dietary diversity among children through previous bivariate analysis and literature review, we employ a multilevel logistic model to assess their combined impact on the prevalence of inadequate minimum dietary diversity (MDD). In this multilevel logit model, we categorize units at the lower level (level 1) as children nested within higher-level units, which include clusters and provinces (level 2 and level 3). The regression results, expressed in terms of log odds along with a 95% confidence interval (CI), are presented in Table 2. Year-specific odds ratios for children failing to meet the minimum dietary diversity criteria, compared to those meeting MDD, are displayed in Columns II to IV. The final Column V, presents the outcomes of Model V, which combines data from all four rounds of surveys.

**Table 2:** Multilevel logistic regression results of inadequate minimum dietary diversity and associated factors among children aged 6–23 months in Cambodia: based on 2005–2021 DHS.

Variable	CDHS 2005	CDHS 2010	CDHS 2014	CDHS 2021	Combined
	Odds Ratio [95% CI]	Odds Ratio [95% CI]	Odds Ratio [95% CI]	Odds Ratio [95% CI]	Odds Ratio [95% CI]
Child age					
6–11 months	1.000	1.000	1.000	1.000	1.000
12–17 months	0.383 [0.289, 0.507]	0.405 [0.313, 0.524]	0.337 [0.262, 0.435]	0.488 [0.392, 0.607]	0.421 [0.373, 0.475]
18–23 months	0.380 [0.284, 0.509]	0.521 [0.399, 0.680]	0.397 [0.309, 0.510]	0.491 [0.392, 0.615]	0.465 [0.411, 0.526]
Child Sex					
Male	1.000	1.000	1.000	1.000	1.000
Female	1.195 [0.961, 1.485]	1.061 [0.866, 1.301]	1.053 [0.864, 1.284]	1.045 [0.874, 1.248]	1.101 [1.001, 1.212]
Birth order					
First	1.000	1.000	1.000	1.000	1.000
Second or third	0.489 [0.059, 4.028]	0.517 [0.050, 5.299]	0.623 [0.075, 5.143]	0.262 [0.018, 3.790]	0.504 [0.164, 1.541]
Fourth or higher	0.509 [0.060, 4.274]	0.48 [0.045, 5.031]	0.516 [0.060, 4.379]	0.340 [0.022, 5.068]	0.531 [0.171, 1.643]
Mother’s age					
15–19 yrs	1.000	1.000	1.000	1.000	1.000
20–34 yrs	0.921 [0.524, 1.621]	0.677 [0.403, 1.139]	0.873 [0.553, 1.378]	1.274 [0.837, 1.939]	0.948 [0.750, 1.198]
35–49 yrs	0.939 [0.493, 1.790]	0.882 [0.474, 1.642]	0.861 [0.484, 1.532]	1.070 [0.655, 1.749]	0.910 [0.690, 1.200]
Mother’s education					
No education	1.000	1.000	1.000	1.000	1.000
Primary	0.916 [0.678, 1.238]	0.733 [0.527, 1.018]	0.883 [0.621, 1.257]	0.781 [0.569, 1.072]	0.830 [0.710, 0.971]
Secondary	0.855 [0.562, 1.298]	0.445 [0.299, 0.663]	0.811 [0.542, 1.215]	0.675 [0.475, 0.958]	0.693 [0.576, 0.833]
Higher	0.663 [0.141, 3.123]	0.476 [0.205, 1.105]	0.850 [0.435, 1.661]	0.551 [0.309, 0.983]	0.633 [0.446, 0.897]
Mother’s occupation					
Not working	1.000	1.000	1.000	1.000	1.000
White collar	0.958 [0.237, 3.873]	0.876 [0.636, 1.207]	1.040 [0.766, 1.414]	0.785 [0.606, 1.017]	0.922 [0.790, 1.076]
Agri worker	0.768 [0.192, 3.071]	1.007 [0.729, 1.389]	1.152 [0.862, 1.539]	1.081 [0.809, 1.446]	0.967 [0.829, 1.129]
Service/manual work	0.996 [0.249, 3.980]	0.873 [0.596, 1.278]	1.145 [0.828, 1.584]	1.008 [0.774, 1.314]	1.077 [0.918, 1.263]
Marital status					
Married	1.000	1.000	1.000	1.000	1.000
Not married	0.683 [0.387, 1.205]	0.678 [0.396, 1.159]	0.874 [0.528, 1.447]	1.395 [0.700, 2.777]	0.837 [0.639, 1.096]
Preceding birth interval					
First birth	1.000	1.000	1.000	1.000	1.000
< 36 months	2.709 [0.328, 22.362]	1.901 [0.184, 19.56]	2.203 [0.263, 18.411]	2.723 [0.187, 39.639]	2.154 [0.702, 6.607]
≥ 36 months	1.767 [0.215, 14.518]	1.502 [0.146, 15.406]	1.499 [0.180, 12.430]	3.173 [0.220, 45.713]	1.683 [0.550, 5.149]
Media exposure					
No	1.000	1.000	1.000	1.000	1.000
Partial	0.508 [0.346, 0.744]	1.029 [0.734, 1.442]	0.675 [0.496, 0.918]	0.749 [0.614, 0.913]	0.696 [0.610, 0.795]
Full	0.494 [0.303, 0.805]	1.105 [0.735, 1.661]	0.439 [0.294, 0.655]	0.673 [0.426, 1.064]	0.619 [0.514, 0.746]
Wanted pregnancy					
Wanted	1.000	1.000	1.000	1.000	1.000
Unwanted/Mistimed	0.694 [0.47, 1.026]	0.999 [0.678, 1.472]	0.984 [0.695, 1.394]	1.163 [0.800, 1.69]	0.925 [0.772, 1.109]
ANC visit during pregnancy					
< 4 visit	1.000	1.000	1.000	1.000	1.000
≥ 4 visit	0.869 [0.667, 1.133]	0.727 [0.57, 0.928]	0.888 [0.679, 1.159]	0.854 [0.66, 1.105]	0.833 [0.735, 0.944]
Place of delivery					
Health facility-Pub	1.000	1.000	1.000	1.000	1.000
Health facility-Pri	1.263 [0.726, 2.198]	0.813 [0.576, 1.146]	0.893 [0.666, 1.197]	0.711 [0.533, 0.95]	0.817 [0.695, 0.961]
Home/Other	0.844 [0.605, 1.178]	1.081 [0.838, 1.396]	1.461 [1.013, 2.107]	0.972 [0.614, 1.538]	1.040 [0.893, 1.211]
Visited health facility in last 12 months					
No	1.000	1.000	1.000	1.000	1.000
Yes	1.054 [0.837, 1.326]	1.025 [0.824, 1.277]	1.041 [0.838, 1.293]	0.694 [0.571, 0.843]	0.889 [0.804, 0.984]
Father’s education					
No education	1.000	1.000	1.000	1.000	1.000
Primary	0.783 [0.552, 1.111]	0.733 [0.505, 1.063]	1.187 [0.817, 1.725]	0.754 [0.545, 1.044]	0.785 [0.663, 0.930]
Secondary	0.643 [0.432, 0.956]	0.649 [0.435, 0.967]	0.95 [0.638, 1.415]	0.774 [0.551, 1.085]	0.683 [0.569, 0.818]
Higher	0.787 [0.331, 1.871]	0.695 [0.36, 1.345]	0.755 [0.42, 1.356]	0.700 [0.418, 1.174]	0.633 [0.473, 0.847]
Father’s occupation					
Not working	1.000	1.000	1.000	1.000	1.000
White collar	0.540 [0.175, 1.657]	2.651 [0.858, 8.193]	1.105 [0.415, 2.939]	0.900 [0.548, 1.48]	0.878 [0.636, 1.211]
Agri worker	0.788 [0.264, 2.354]	2.559 [0.853, 7.678]	0.960 [0.352, 2.62]	0.918 [0.576, 1.464]	0.879 [0.644, 1.199]
Service/manual work	0.748 [0.250, 2.233]	2.145 [0.715, 6.432]	0.920 [0.341, 2.478]	1.138 [0.728, 1.777]	0.878 [0.649, 1.188]
Place of residence					
Urban	1.000	1.000	1.000	1.000	1.000
Rural	1.323 [0.970, 1.804]	0.752 [0.543, 1.041]	0.857 [0.628, 1.172]	1.082 [0.85, 1.376]	0.973 [0.846, 1.118]
Wealth index					
Poorest	1.000	1.000	1.000	1.000	1.000
Poorer	0.924 [0.659, 1.296]	0.978 [0.698, 1.37]	0.906 [0.654, 1.256]	0.808 [0.607, 1.076]	0.931 [0.798, 1.086]
Middle	0.762 [0.534, 1.087]	1.232 [0.863, 1.759]	1 [0.688, 1.454]	1.130 [0.831, 1.536]	1.067 [0.903, 1.260]
Richer	0.648 [0.434, 0.965]	0.835 [0.570, 1.223]	0.765 [0.516, 1.135]	0.928 [0.671, 1.282]	0.831 [0.695, 0.993]
Richest	0.637 [0.386, 1.052]	0.718 [0.446, 1.158]	0.662 [0.415, 1.057]	0.821 [0.542, 1.244]	0.758 [0.608, 0.944]
Household size					
≤ 4	1.000	1.000	1.000	1.000	1.000
5–9	1.064 [0.815, 1.389]	0.744 [0.584, 0.948]	1.146 [0.909, 1.445]	0.953 [0.785, 1.155]	0.955 [0.855, 1.067]
≥ 10	1.108 [0.697, 1.763]	0.742 [0.477, 1.153]	1.045 [0.678, 1.612]	0.862 [0.512, 1.453]	0.988 [0.793, 1.231]
Sex of head of household					
Male	1.000	1.000	1.000	1.000	1.000
Female	1.029 [0.746, 1.421]	1.463 [1.123, 1.904]	0.996 [0.782, 1.268]	0.986 [0.800, 1.216]	1.12 [0.992, 1.266]
Year of survey					
2005					1.000
2010					0.832 [0.7000, 0.99]
2014					0.558 [0.461, 0.675]
2021					0.340 [0.274, 0.421]

Source: Authors estimation using Demographic and Health Survey (DHS) Data.

The odds of children aged 12–17 months having inadequate MDD were 63% lower compared to those aged 6–11 months. When we examine households from the poorest to the richest, the odds of inadequate MDD among children decreased by 24%. Children with highly educated mothers were nearly 37% less likely to have inadequate MDD than mothers with no formal education. Girls aged 6–23 months had a 10% higher likelihood of inadequate MDD compared to boys of the same age group. Exposure to media among mothers decreased the odds of inadequate MDD in children; this finding remained consistent over the years, except for the 2010 survey. Children whose mothers received full antenatal care (ANC) during pregnancy and delivered in a private healthcare facility had lower odds compared to mothers with less than four ANC visits and who delivered in a public health facility. Finally, children whose mothers had visited a healthcare facility in the past 12 months before the survey were nearly 11% less likely to have inadequate MDD. Interestingly, we did not detect a significant association between the place of residence and the likelihood of inadequate MDD. The multilevel analysis examining inadequate consumption of each of the eight food groups, along with the variables utilized in the empirical analysis, is presented in Supplementary file (Appendix 2, Tables 1–8). The results reveal a similar pattern of association between inadequate consumption and the child’s age, sex, education, parent’s occupation, and household wealth status.

**Figure 9:**
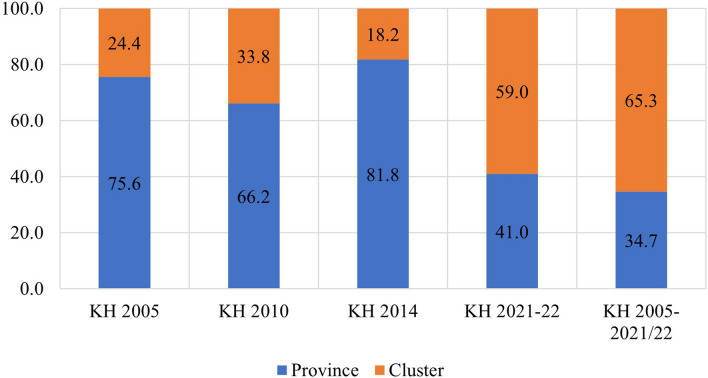
Proportion of variance partitioned between clusters, and provinces for inadequate consumption of other rich fruits and vegetables.

**Figure 10:**
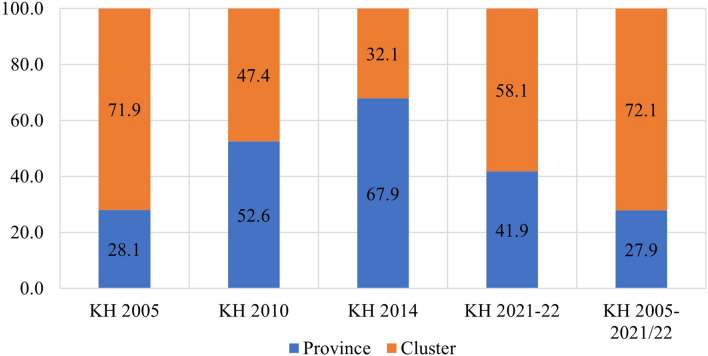
Proportion of variance partitioned between clusters, and provinces for inadequate consumption of Eggs.

**Figure 11:**
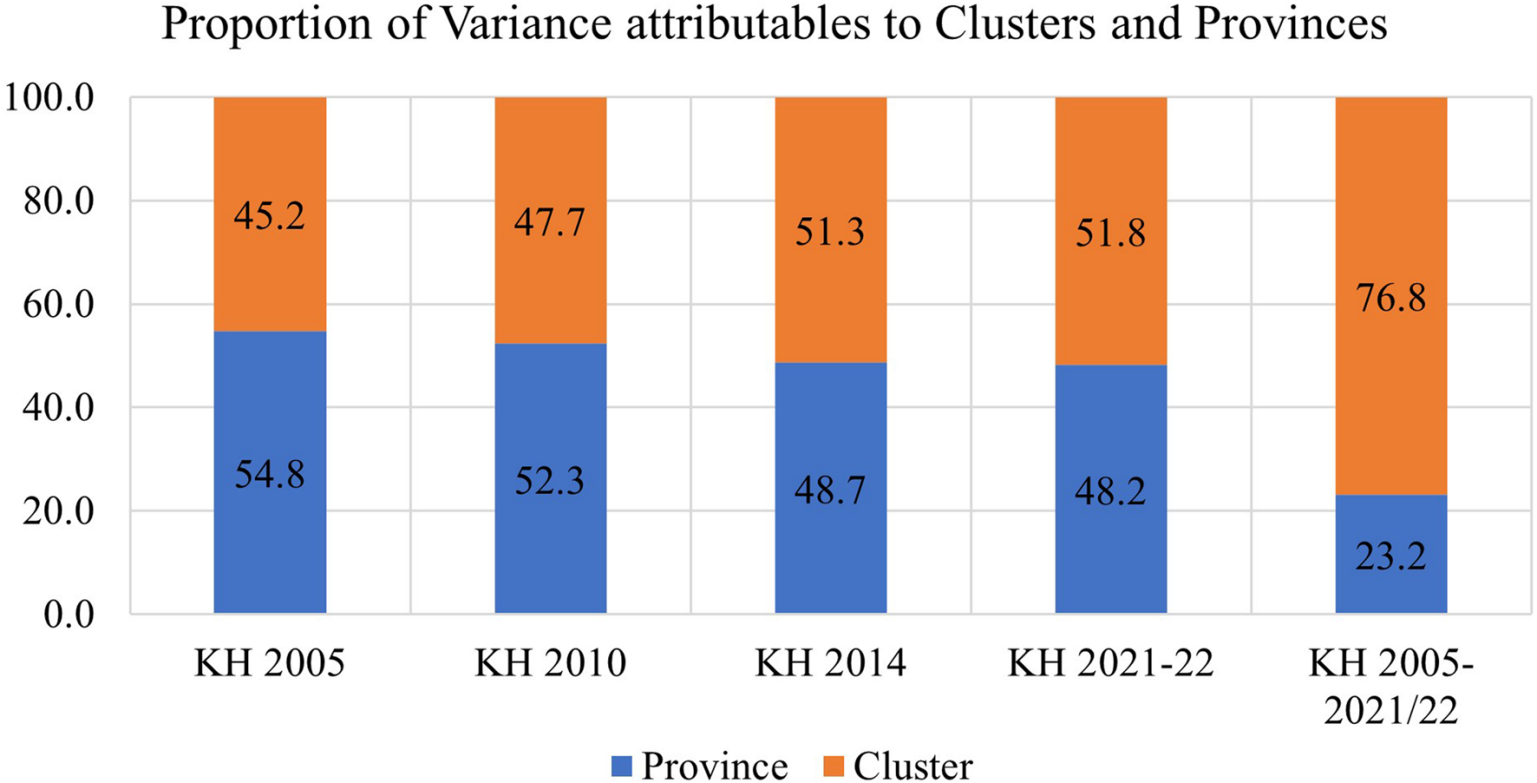
Proportion of variance partitioned between clusters, and provinces for inadequate consumption of Flesh Foods.

**Figure 12:**
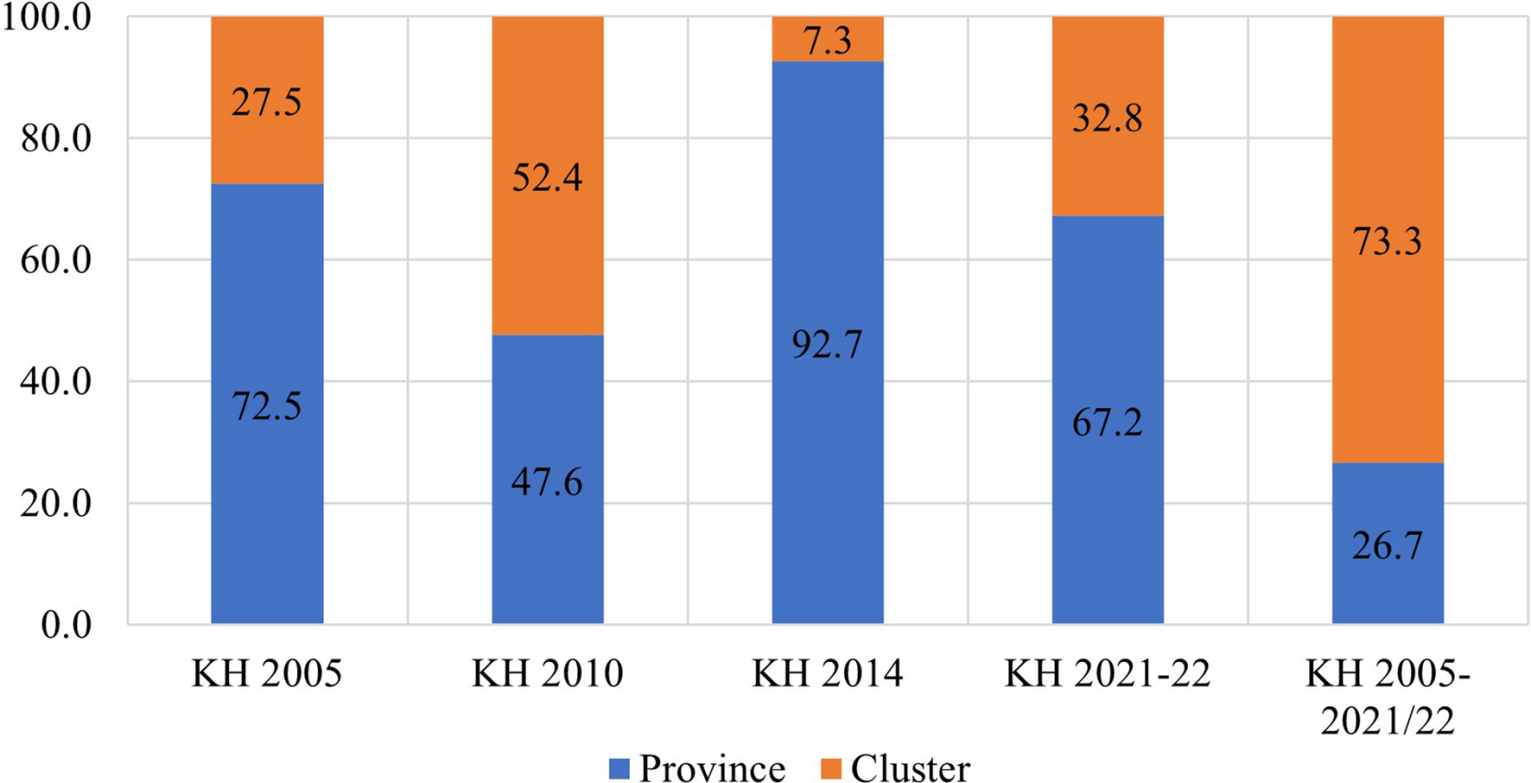
Proportion of variance partitioned between clusters, and provinces for inadequate consumption of Legumes and nuts.

The study found that in the pooled data from all survey rounds, clusters represented the primary source of geographic variation for inadequate MDD and each constituent food group. In contrast, provinces accounted for the least amount of geographic variation among all nine outcomes. Upon examining the trends in variation across the four surveys, the following findings emerged: The food groups comprising Vitamin A-rich fruits and vegetables, flesh foods, and other fruits and vegetables exhibited higher variation at the province level until 2010 and 2014, after which they displayed a reverse trend. Conversely, food groups like eggs and dairy products initially showed greater variation at the cluster level, but over time, the province emerged as the primary source of variation. The percentages of geographic variation for different categories of food, as illustrated in Figs. 4, 5, 6, 7, 8, 9, 10, 11 and 12, were computed after adjusting for specific covariates associated with child malnutrition, such as the child’s age, gender, household wealth, dwelling setting, mother’s education, and ANC visits, among others. Figure 4 shows that in 2005, the share of variance in minimum dietary diversity due to provincial-level factors was 63.2%, while it was only 36.8% from cluster-level factors cluster, which gradually changed to 55.5% and 44.5% in 2021/2022. Figure 5 indicates a greater variation in breastfeeding at the cluster level in all the years. For the dairy products, the share of variation due to cluster factor declined from 80.2% in 2005 to 22.2% in 2021/22 (see Fig. 6). For grain, roots, and tubers, as shown in Fig. 7, the share of variation due to cluster factor declined from 79.3% in 2005 to 29.8% in 2021/22. Figure 8 shows that in the case of vitamin-rich fruits and vegetables, the share of variation due to cluster factor increased from 43.6% in 2005 to 60.2% in 2021/22. For other fruits and vegetables, the share of variation due to the cluster factor increased from 24.3% in 2005 to 59% in 2021/22 (Fig. 9). It can be seen in Fig. 10 that in the case of eggs, the share of variation due to the cluster factor decreased from 71.9% in 2005 to 58% in 2021/22. For flesh food, the share of variation due to cluster factor is consistent around 50% (Fig. 11). For nuts and legumes, there was no pattern, but in 2021/22, the share of variation due to cluster was 32.8% while that of the province was 67.2% (Fig. 12).

Table 3 clearly illustrates that in the most recent survey year, children aged 6–23 months exhibited a lower prevalence of inadequate MDD compared to those reported in the year 2005. Out of the total difference in inadequate MDD within this group, approximately 9% can be attributed due to endowment effects, which are explained by changes in the composition of children. The remaining 91% of the unexplained difference was driven by the coefficients and interaction effects of the selected explanatory variables, the result of the interaction effect was found to be insignificant at the 5% significance level. The coefficient effect accounted for approximately 118% of the variation in inadequate minimum dietary diversity.

**Table 3:** Oaxaca–Blinder decomposition: year-wise difference in inadequate MDD among children aged (6–23 months), Cambodia (2005–2021–22).

	Coeff.	SD	p value	LL (95% CI)	UL (95% CI)
2005	0.773	0.009	0.000	0.756	0.790
2021–2022	0.547	0.010	0.000	0.528	0.567
Difference	0.225	0.013	0.000	0.199	0.252
Endowments	0.019	0.041	0.043	0.032	0.100
Coefficients	0.266	0.036	0.000	0.196	0.336
Interaction	−0.060	0.053	0.259	−0.164	0.044

Source: Authors estimation using Demographic and Health Survey (DHS) Data.

## Discussion

Inadequate Infant and Young Children Feeding (IYCF) practices pose a universal concern, particularly in developing countries such as Cambodia. These practices hugely contribute to malnourishment and impact optimal growth in the initial life years. Hence, tackling the modifiable determinants of malnutrition is a priority to improve child health^15^. This study’s primary objective was to assess the trend estimates of inadequate Minimum Dietary Diversity (MDD) intake and the associated factors affecting children aged 6–23 months in Cambodia. Our investigation yielded four notable findings:

First, we observed that clusters represented the most significant source of geographic variation regarding children’s dietary patterns, particularly with respect to all eight food groups and MDD. Moreover, in a recent year survey, province emerged as the primary source of geographic variation in children failing to meet MDD criteria, not consuming dairy products, not consuming legumes and nuts, and not consuming grains and roots. This underscores the importance of dissecting the consumption of each food group based on geographical variations. The variation at the cluster level suggests that, even within provinces with a higher percentage of children not meeting MDD or not consuming specific food groups, there may still be areas within the province with significantly higher percentages of children facing these dietary inadequacies. This highlights the persistence of inequality in nutritional access.

Secondly, over the course of four successive surveys, our findings revealed an upward trend in the minimum dietary diversity requirement for children over a 16-year span in Cambodia’s history. However, it is concerning that more than 50% of children still do not have access to a diverse diet. In comparison to figures from other Asian countries, our findings align closely with those from Indonesia (46%) (IDHS)^16^, and the Philippines (53%) (PDHS)^17^. Yet, these figures are considerably lower than the findings reported in India (72%)^18^, and Pakistan (85%)^18^, which suggests that children aged 6–23 months face inadequate access to MDD across different countries. This discrepancy may be attributed to variations in food cultures^19–21^. Some societies may place a greater emphasis on agricultural products in child feeding, while others rely more on animal products^21^. Additionally, factors such as geographical variations, population growth, and the socioeconomic status of countries might have contributed to these differences. Studies have shown that children in Sub-Saharan Africa (SSA) experience growth challenges from early infancy through the second year of life^22^. It is crucial to prioritize governmental efforts focused on enacting national nutritional initiatives and addressing cultural beliefs related to complementary feeding^23,24^. Moreover, factors like food availability and accessibility play a significant role in achieving adequate MDD^25^.

Thirdly, this study revealed that the child’s age and sex, mother’s age and education, and household wealth are linked with Minimum Dietary Diversity (MDD) in Cambodia. It is evident from the study that inadequate MDD decreased as the age of the child increased, corroborating with prior research in countries such as Indonesia^26^, Bangladesh^27^, and SSA countries^28^. This could be attributed to the general perception of mothers regarding the inadequate digestive capacity of younger children for certain food items such as bananas, eggs, and pumpkins^29^, resulting in a delayed introduction of a diversified diet, or restricting their diet to just milk or cereals^30^. Factors such as teething and increased infections, which can lead to appetite loss and weight reduction, might affect feeding practices amongst children aged 6–8 months^31^. Older children’s acceptance of diverse food textures, differing tastes, and increasing familiarity with various foods could be potential reasons for this association^32^.

Differences in socio-economic status were evident in our observations, where children from affluent households tend to display greater dietary variety compared to those from less privileged families. This could be attributed to enhanced accessibility to food purchased from sources market^33,34^. An interesting trend noted is that as mothers’ education, employment statuses, and media exposure levels improve, their children are more likely to meet MDD^19,20,25,27,35^. A possible explanation is that working mothers tend to have a stronger socio-economic standing, which enables them to ensure a balanced diet and, consequently, influences their approach to complementary feeding practices^36^. Employed mothers often have higher levels of education, which can lead to more informed household decision-making regarding purchasing and providing appropriate foods for their children^37^. The findings suggest that children whose mothers have media access are less prone to inadequate MDD. Media is widely perceived as a dependable source of health and nutrition information, contributing to educating mothers and encouraging behavioral shifts^28,36^.

Additionally, health service-related factors, such as children whose mothers have had full antenatal care (ANC) visits, delivered at health facilities, and visited healthcare facilities within the last 12 months of the survey, had a lower likelihood of inadequate MDD. This finding aligns with studies conducted in South Asia and African countries^20,30,35^. The effective communication and counseling on nutrition and baby care provided by the health workers during the ANC visits may help to improve the dietary diversity among children^20^. We found that girls in Cambodia were disproportionately at risk of not meeting MDD. This finding differs from past studies indicating no difference in infant and young child feeding practices in other countries^35^.

### Strengths and limitations of the study

The study’s robustness in transforming discourse surrounding minimum dietary diversity (MDD) needs lies in its comprehensive examination of sociocultural and ecological influences on behavior. Employing a hierarchical framework, we positioned children within clusters and clusters within provinces. This approach facilitated the identification of pivotal influencers across various tiers (cluster and province), offering distinctive perspectives on multifaceted interventions crucial for reshaping discussions on the need for minimum dietary diversity, a critical aspect of child health nationwide. These insights will empower the government to craft strategies tailored to hyperlocal contexts, effectively addressing nutritional needs.

This study has a couple of limitations. Firstly, the responses to questions about food consumption rely on self-reporting and pertain to the food consumed in the 24 h preceding the interview, even though many foods are consumed less frequently than that^38^. Secondly, this study employs food groups defined by WHO. As a result, we have not evaluated the intake of conventional foods that are commonly found in Cambodia and serve as significant sources of vitamin A and protein^39^. Using an Oaxaca–Blinder approach has its own limitations; it might be difficult to infer the main cause of the unexplained part of the temporal change in inadequate MDD. Another challenge arises from the “indicator problem”, wherein the choice of the omitted or base category may influence the outcomes related to categorical variables in the model. While this doesn't impact the explained portion of the gap, it can influence the extent to which the unexplained portion is attributed to variations in the intercept as opposed to differences in the coefficient estimates^40^.

## Conclusion

Inadequate Infant and Young Children Feeding (IYCF) practices, especially prevalent in developing nations like Cambodia, remain a pressing global concern, contributing significantly to malnutrition and hindering optimal growth during crucial early life stages. Addressing the modifiable determinants of malnutrition stands as a paramount priority for enhancing child health. This study assesses trends in inadequate Minimum Dietary Diversity (MDD) and associated factors among children aged 6–23 months in Cambodia and reveals several key insights.

Firstly, geographic disparities, notably at the cluster level, underscore the persistence of nutritional inequality, necessitating targeted interventions even within provinces with seemingly higher MDD rates. Secondly, despite an upward trend in meeting MDD requirements over 16 years, more than half of Cambodian children lack access to a diverse diet, aligning with regional trends but lagging behind counterparts in Asia. The observed differences in dietary diversity can be attributed to cultural, geographical, and socio-economic factors, emphasizing the need for tailored nutritional initiatives and addressing cultural beliefs. Furthermore, maternal and household characteristics significantly influence MDD, with maternal education, employment, and media access emerging as pivotal determinants of dietary adequacy. Notably, health service-related factors, such as full antenatal care and healthcare facility visits, correlate with improved dietary diversity, highlighting the role of healthcare interventions in promoting optimal feeding practices. However, gender disparities persist, with girls facing a higher risk of inadequate MDD. These findings emphasize the multifaceted nature of child nutrition, urging comprehensive strategies encompassing education, healthcare, and cultural sensitivities to ensure equitable access to nutritious diets for all Cambodian children. Efforts must prioritize empowering mothers, enhancing healthcare services, and fostering a supportive environment conducive to healthy feeding practices, thereby mitigating the pervasive threat of childhood malnutrition in Cambodia.

## Method

### Data source

The current study utilized data from multiple rounds of the Cambodia Demographic and Health Survey (CDHS). Up to the present time, five rounds of CDHS have been conducted, spanning from 2000 to 2022. The National Institute of Statistics (NIS), operating under the Ministry of Planning, spearheaded the implementation in conjunction with the Ministry of Health. This Demographic and Health Survey received financial support from the United States Agency for International Development (USAID) and technical support from the Inner City Fund (ICF).

The CDHS, a nationally representative survey based on the population, employed a two-stage stratified cluster sampling design for participant selection. For first-stage sampling, a specific number of enumeration areas (EAs or clusters) were chosen in a manner that was proportionate to their size, while considering urban and rural distinctions. In the subsequent stage, a defined number of households (usually 25–30) were randomly selected from the listed households within each EA, utilizing a simple random selection process. The survey covered eligible women (aged 15–49), men (aged 15–54), and children under the age of 5 years residing in the selected households. The objective of the survey was to furnish estimates related to fertility, mortality, maternal and child health care services, and reproductive health facilities at both national and sub-national levels. Comprehensive details concerning the study’s design, sampling method, framework, and non-response rate are available in specific reports for each round of the survey^11,41–43^.

We extracted data from the last four rounds of the CDHS Kid’s records for current research. The initial round of the survey was excluded due to the absence of information on the dietary diversity variable, which stands as the focal point of interest. The guidelines from the Demographic and Health Survey pertaining to dietary diversity calculations are intended for the youngest child aged between 6 and 23 months, who resides with their mother. Accordingly, the final sample encompassed 9056 youngest children falling within the age bracket of 6 to 23 months, currently living with their mothers. This amalgamation includes data from CDHS 2005 (2020 participants), CDHS 2010 (2372 participants), CDHS 2014 (2143 participants), and CDHS 2021–2022 (2321 participants).

### Dependent and independent variables

The outcome variable of interest is inadequate minimum dietary diversity. In the survey, the mother or caregiver of the child was inquired about the types of food the child had eaten in 24 h leading up to the interview. These food groups are: (i) breast milk, (ii) dairy products (milk, yogurt, cheese, infant formula), (iii) grains, roots, and tubers, (iv) vitamin A-rich fruits and vegetables, (v) other fruits and vegetables (vi) eggs, (vii) flesh foods (viii) legumes and nuts. Aligned with the most recent guidelines from the WHO (WHO, 2017) and the Demographic and Health Surveys (as outlined in the Guide to DHS Statistics DHS-7)^44^, we assign a score of 1 for each food group consumed, while non-consumption was denoted by a score of 0. The total score was then calculated by summing the scores across all food groups. Subsequently, a binary outcome variable was generated to compute the inadequate MDD. Children who consumed five or more groups were assigned a score of “0”, while those who consumed fewer than five groups were assigned a score of “1”. Until 2017, adequate MDD intake was defined as consuming four out of seven food groups. The revised indicator acknowledges breast milk as an additional food group and changes the criterion for achieving MDD. It now requires the consumption of a minimum of 5 food groups out of 8, in contrast to the previous requirement of a minimum of 4 food groups out of 7^45,46^. To maintain homogeneity and ensure the comparability of outcome variables across the surveys, we followed revised guidelines to compute inadequate MDD in each survey round.

To measure the disparity in inadequate MDD, we computed the inequality for all eight food groups that make up the MDD separately. Considering this, we ended with a total of nine outcomes: (1) not meeting the MDD, (2) not currently breastfeeding, (3) did not consume dairy, (4) did not eat grains, roots, and tubers, (5) did not eat vitamin A-rich fruits and vegetables, (6) did not eat other fruits and vegetables, (7) did not eat eggs, (8) did not eat flesh foods, (9) did not eat legumes and nuts^26^.

To establish the conceptual framework for comprehending the variations in failing to achieve minimum dietary diversity, distinct determinants were selected at both child and maternal levels. These selections were guided by insights gleaned from the literature review, primarily due to their established associations with MDD^9,26,47–49^. Child-related factors encompassed the child’s sex, age recategorized from a continuous scale into three groups (6–11, 12–17, and 18–23 months), and birth order reclassified as “first,” “second or third,” and “fourth or higher.”

Maternal factors included maternal age, categorized into three groups (15–29, 30–39, and 40–49 years old), employment, educational, and marital status, preceding birth interval categorized into three groups (First birth, < 36 months, and ≥ 36 months), media exposure combining newspapers, radio, and television into levels: no exposure, partial (two media), and full (all three), and wealth quintiles derived through asset indices based on household attributes like amenities and materials. A wealth score, a pivotal variable reflecting data variability, was computed through principal component analysis. Urban/rural households were assessed separately, and the indices were standardized. Households were divided into quintiles based on wealth scores, ranging from the poorest (20%) to the richest (20%)^50^.

The study also considered place of residence (rural vs. urban), household gender composition, and size categorized into three groups (< 4, 5–9, and ≥ 10 members). Paternal factors, including the father’s education and occupation, were included as well. Health service utilization factors encompassed the number of antenatal care (ANC) visits during pregnancy (categorized into < 4 visits and ≥ 4 visits), place of delivery, and visits to healthcare facilities in the last 12 months (yes vs. no).

### Analytical approach

The data from the CDHS demonstrate a hierarchical structure, wherein children aged 6–23 months form the first level nested within clusters at the second level and provinces at the third level. This hierarchical arrangement could potentially violate standard logistic regression assumptions, such as independence and equal variance^51^. Given the presence of regional heterogeneity, a single-level model proves insufficient, leading to inaccuracies in parameter estimation. To evaluate how various layers of explanatory variables impact inadequate minimum dietary diversity and account for variations at the cluster and province levels, we utilized a multilevel binary regression model. This approach allows for a simultaneous examination of effects at both group levels (clusters and provinces) and individual levels on outcomes while addressing the lack of independence among observations within groups^52^. Hierarchal analysis enables the investigation of both inter-group and intra-group variability, as well as the relationship between variables at both group and individual levels. To achieve this, we employed a three-level variance component model. This model initially decomposes the overall geographic variation into clusters and provinces, with respect to the probability of a child “i” in cluster “j” and province “k” for inadequate MDD or inadequate consumption of each of the food groups, utilizing the Eq. (1)

1 $$log\frac{\left({\pi }_{ijk}\right)}{\left({1-\pi }_{ijk}\right)}= \alpha +{X}_{ijk}\beta +{\mu }_{jk}+{\Omega }_{k}$$

where, subscript $i,j, k$ denote children, cluster, and province, respectively.

${\pi }_{ijk}$ is the probability of i^th^ children of cluster j and province k inadequate MDD.

$\alpha$ is the intercept that is the effect of feeding inadequate minimum DD when the effect of all explanatory variables is absent.

${X}_{ijk}$ selected socio-economic and demographic characteristics for i^th^ children of cluster j and province k.

$\beta$ vector of constants giving the log odds resultant from one unit change in variable ${X}_{ijk}$

${\mu }_{jk}$ & ${\Omega }_{k}$ are the random effect for cluster $j$ and province $k$

Each of the residual differentials is assumed to be normally distributed with a mean of zero and variances of ${\sigma }_{\mu }^{2}$ and ${\sigma }_{\Omega }^{2}$ , variances quantify the between-cluster (${\sigma }_{\mu \Omega }^{2}$), between-district (${\sigma }_{\Omega }^{2}$) variation. The variance at level one (children) is assumed to be a constant in binary models^53,54^. Furthermore, we investigated the proportion of geographic variation attributed to clusters and provinces for each of the nine outcomes within the hierarchical model. This was achieved by dividing the variance at a specific level by the total geographic variation (i.e., for the cluster level, $\frac{{\sigma }_{\mu }^{2}}{{\sigma }_{\mu }^{2}+{\sigma }_{\Omega }^{2}}$).

The initial and final survey years, 2005 and 2021–22, were divided into two groups to assess factors contributing to inadequate MDD disparities between the two periods, and non-linear Binder Oaxaca decomposition analysis was employed^55–57^. The Blinder–Oaxaca technique was first introduced by Blinder and Oaxaca in 1973^58,59^. This approach segregates differences into three parts: explained endowment, coefficient, and unexplained interaction. Endowment signifies variation due to variable changes, while coefficient results from variable composition shifts. For instance, in our study, focusing on inadequate MDD, a child’s age influences it. The difference in inadequate MDD attributed to a child’s age change is explained, while unexplained accounts for variations in age effects. The analysis draws from the prevalence of not meeting MDD and coefficients from multivariate binary logistic regression models for each survey year. Compliant with DHS guidelines, analyses incorporated survey weights, clustering, and stratification to ensure national representativeness^60^.

### Ethical approval

Procedures and questionnaires for standard Demographic and Health Surveys (DHS) surveys have been reviewed and approved by the Inner City Fund (ICF) “Alliance for Public Health” Institutional Review Board (IRB). Additionally, country-specific DHS survey protocols are reviewed by the ICF IRB and typically by an IRB in the host country. ICF IRB ensures that the survey complies with the U.S. Department of Health and Human Services regulations for the protection of human subjects (45 CFR 46), while the host country IRB ensures that the survey complies with the laws and norms of the nation. The present analysis utilizes a secondary data set with no identifiable information on the survey participants. This dataset is available in the public domain for research use; hence, no approval was required from any institutional review board as there is no question of human subject protection in this case.

### Informed consent

The DHS survey was anonymous and was administered with the authorization of all participants and the commitment to the privacy and confidentiality of the information gathered.

### Supplementary Information

Supplementary Information.

## Data Availability

The dataset analyzed in the current study is publicly available online on the official website of the Demographic Health Survey Program https://www.dhsprogram.com, and third party is not allowed to share it. Authors do not have the right to upload as required by DHS.

## Abbreviations

ANC: Antenatal Care
CDHS: Cambodia Demographic Health Survey
EA: Enumeration Areas
ICF: Inter City Fund
IYCF: Infant and Young Child Feeding Practice
MDD: Minimum Dietary Diversity
NIS: National Institute of Statistics
UNICEF: United Nations International Children's Emergency Fund
USAID: United States Agency for International Development
WHO: World Health Organization
