# Supplementary material for: Exploring minimum dietary diversity among cambodian children using four rounds of demographic and health survey
Source: Sci Rep. 2024 Jun 26;14:14719. doi: 10.1038/s41598-024-64714-0 (PMC11208556; doi:10.1038/s41598-024-64714-0)
Supplement: Supplementary file 1 — Supplementary Information. [file 41598_2024_64714_MOESM1_ESM.docx]

**Exploring Minimum Dietary Diversity among Cambodian Children Using Four Rounds of Demographic and Health Survey**

**APPENDIX**

**APPENDIX 1**

**PERCENT CHILDREN NOT BREASTFEEDING ACROSS 19 PROVINCES IN CAMBODIA**

| 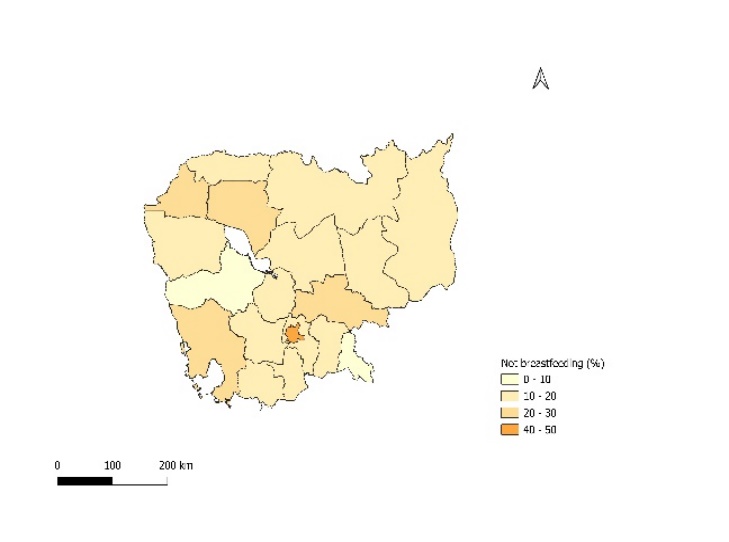  2005 | 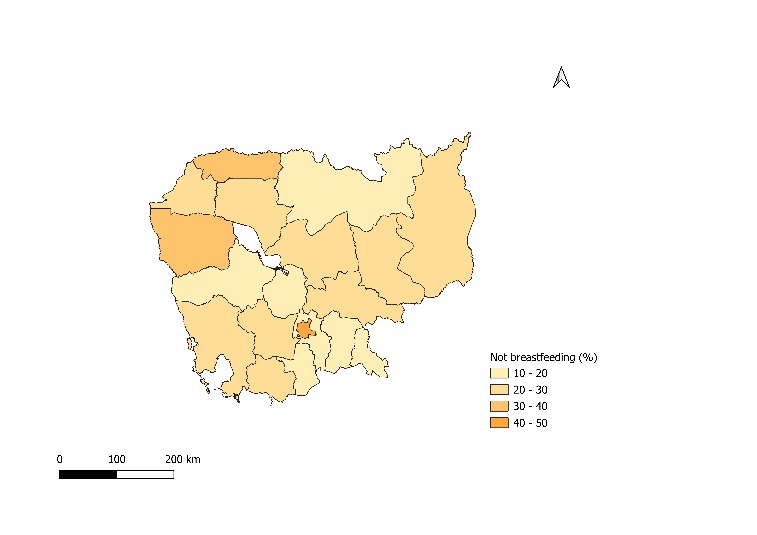  2010 |
| --- | --- |
| 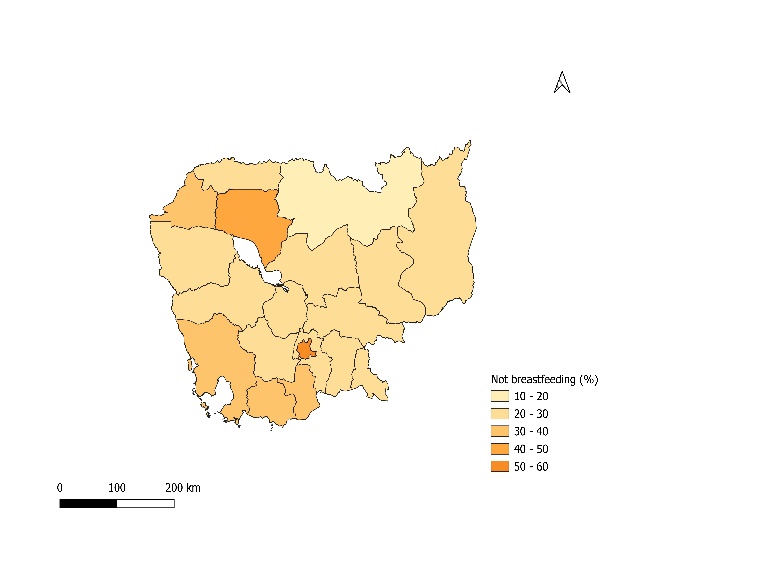  2014 | 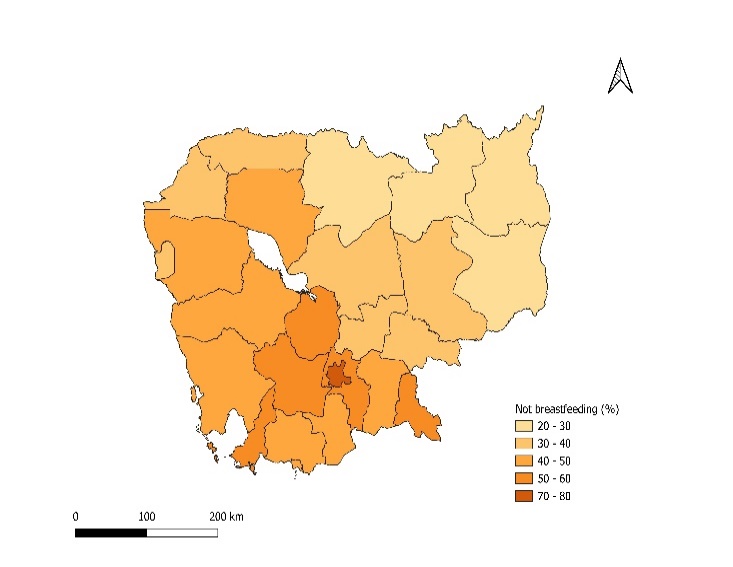  2021-22 |

**Appendix 1 Figure 1|** Geographic distribution of percent children not breastfeeding across 19 provinces in Cambodia.

Source: Authors estimation using Demographic and Health Survey (DHS) Data.

**PERCENT CHILDREN NOT CONSUMING DAIRY PRODUCTS ACROSS 19 PROVINCES IN CAMBODIA**

| 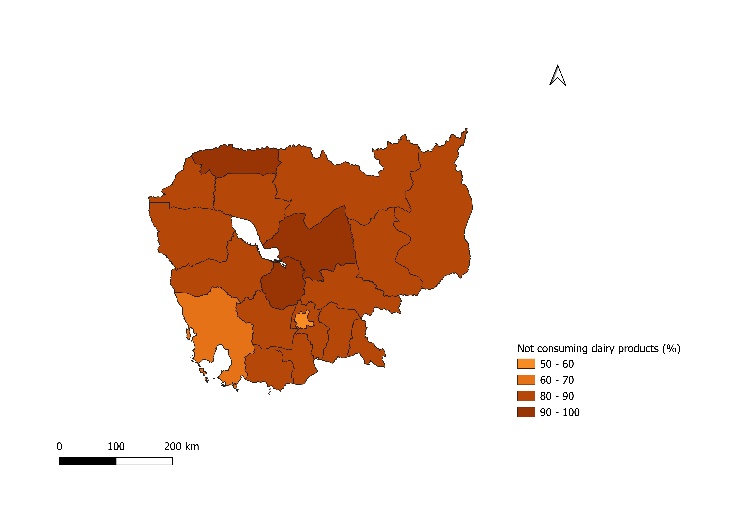  2005 | 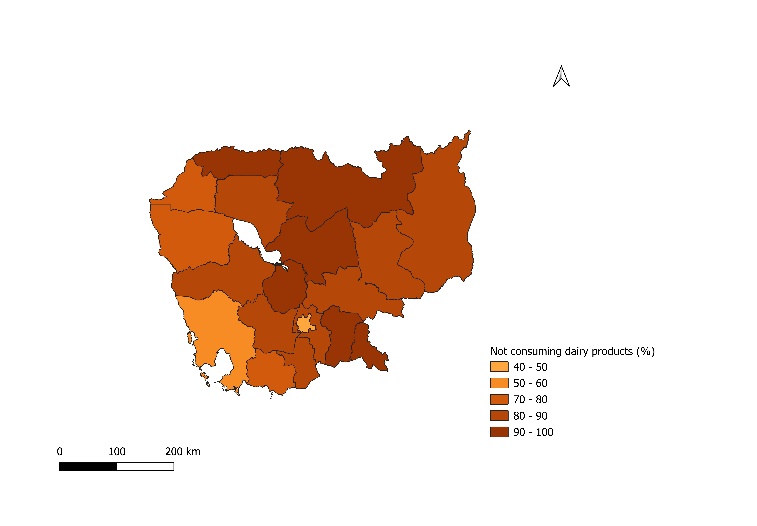  2010 |
| --- | --- |
| 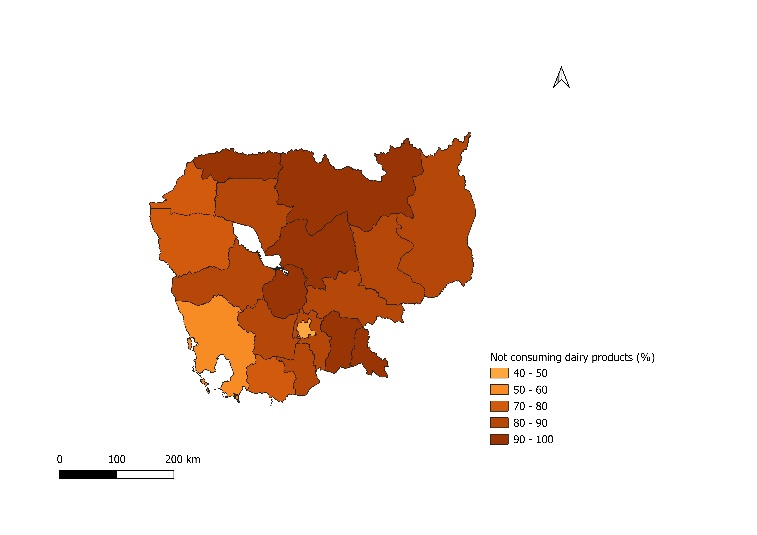  2014 | 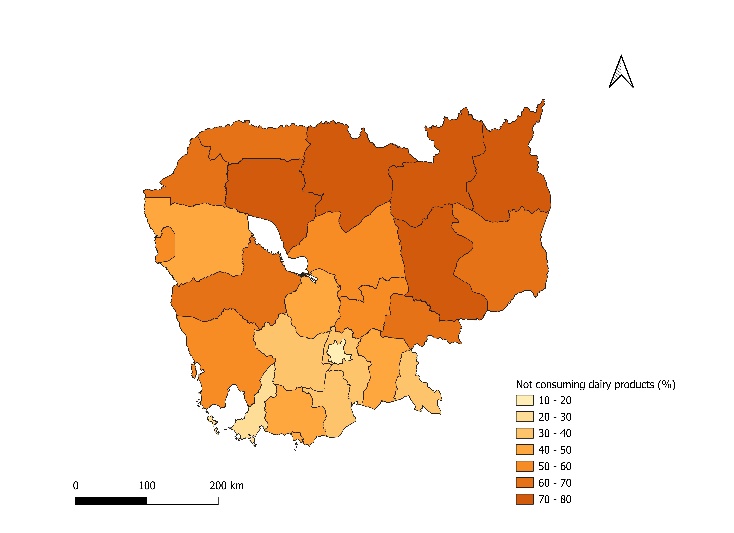  s  2021-22 |

**Appendix 1** Figure 2| Geographic distribution of percent children not consuming dairy products across 19 provinces in Cambodia.

Source: Authors estimation using Demographic and Health Survey (DHS) Data.

**PERCENT CHILDREN NOT EATING GRAINS, ROOTS & TUBERS ACROSS 19 PROVINCES IN CAMBODIA**

| 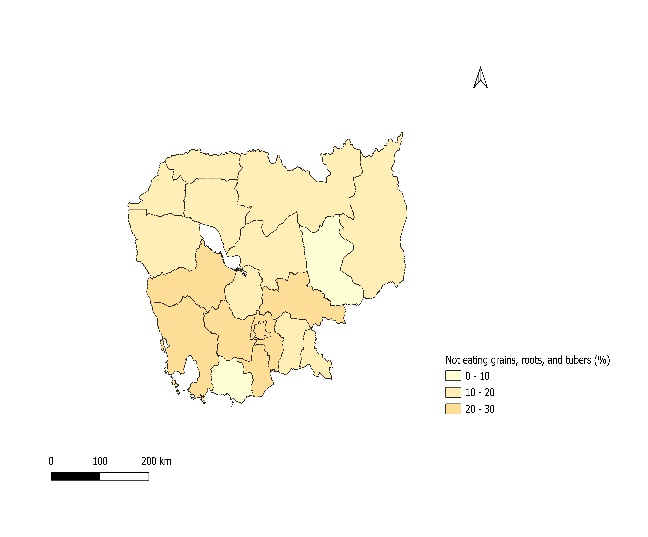  **2005** | 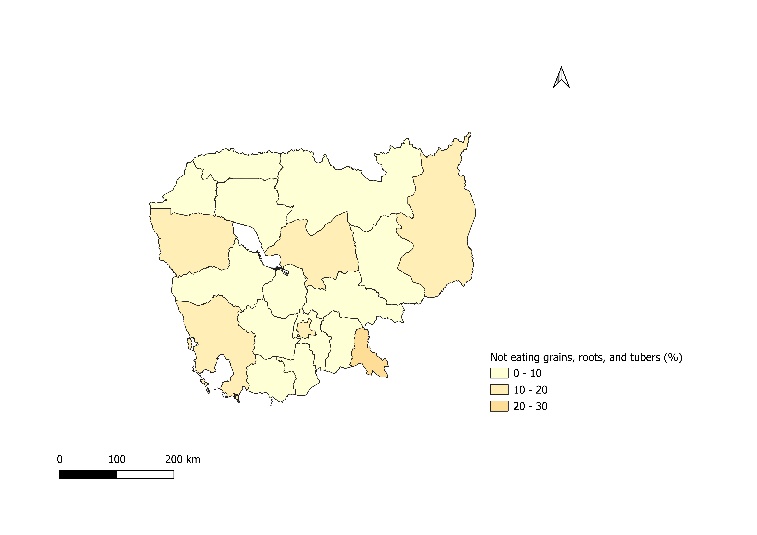  **2010** |
| --- | --- |
| 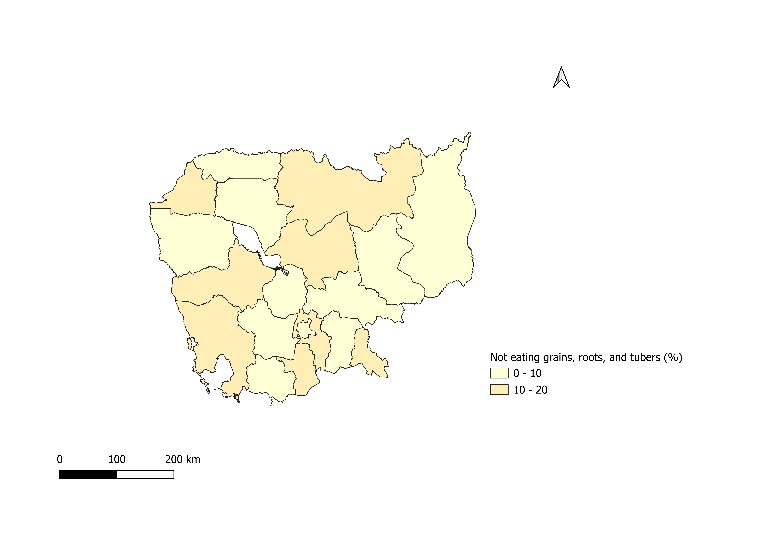  **2014** | 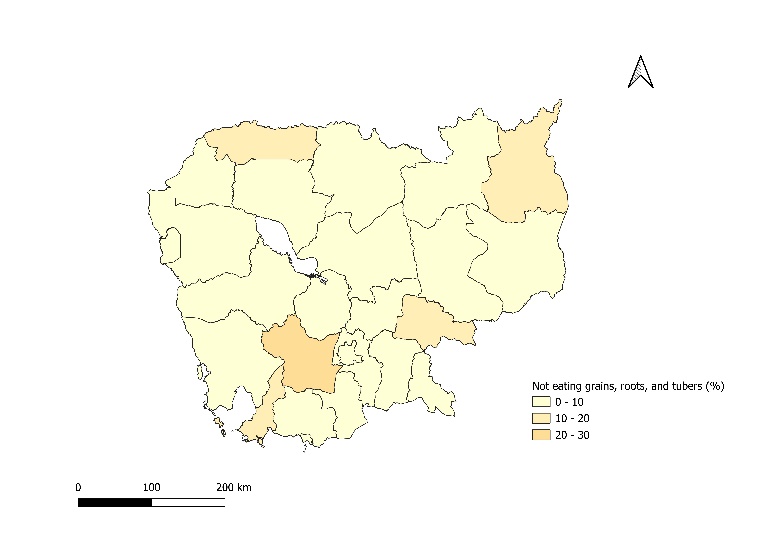  **2021-22** |

**Appendix 1 Figure 3|** Geographic distribution of percent children not eating grains, roots & tubers across 19 provinces in Cambodia.

Source: Authors estimation using Demographic and Health Survey (DHS) Data.

**PERCENT CHILDREN NOT EATING VITAMIN A RICH FRUITS & VEGETABLES ACROSS 19 PROVINCES IN CAMBODIA**

| 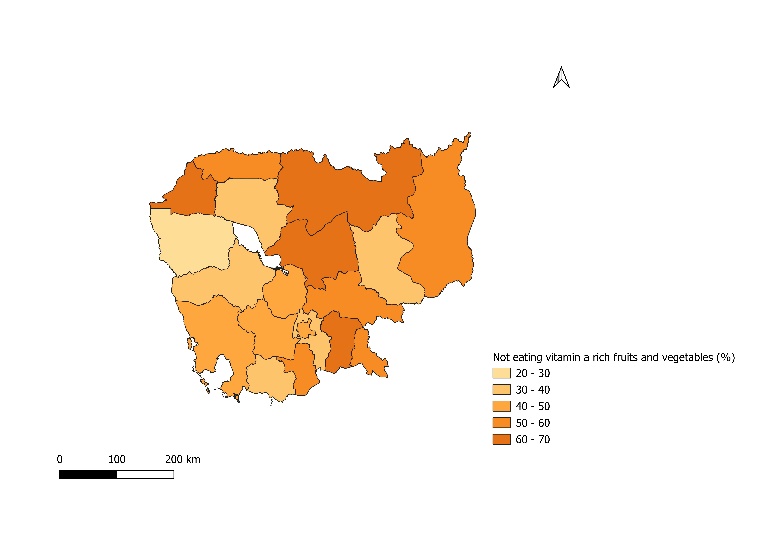  **2005** | 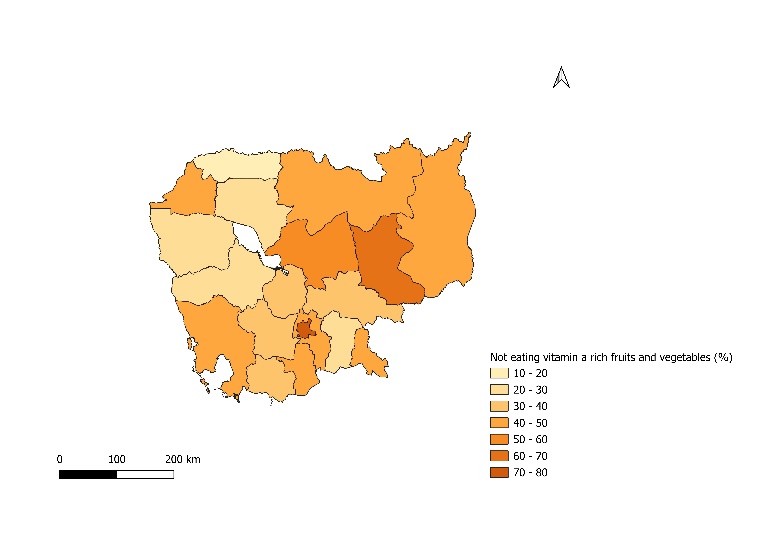  **2010** |
| --- | --- |
| 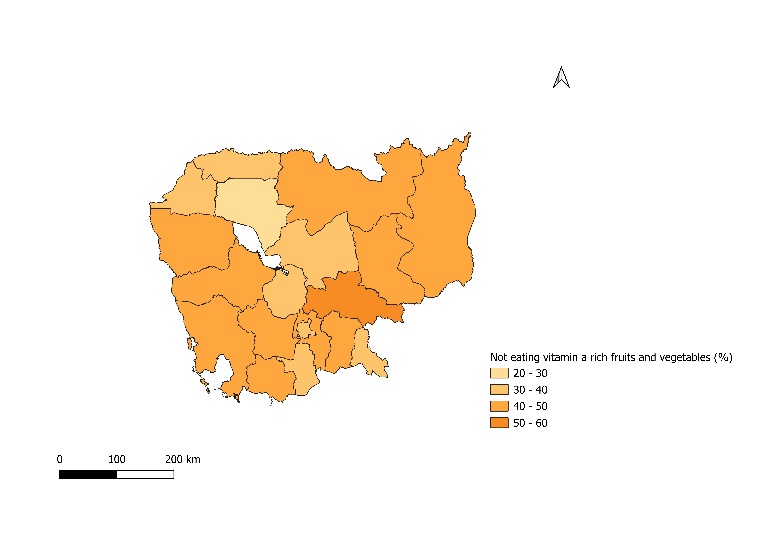  **2014** | 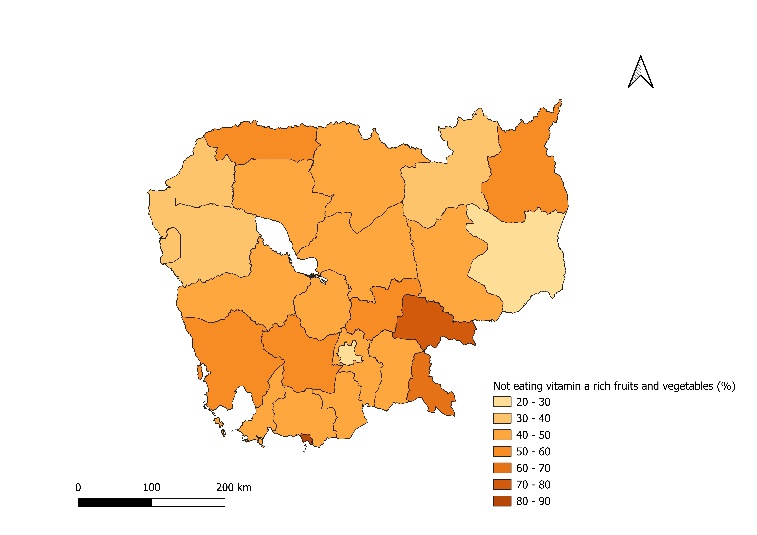  **2021-22** |

**Appendix 1 Figure 4|** Geographic distribution of percent children not eating vitamin A rich fruits & vegetables across 19 provinces in Cambodia.

Source: Authors estimation using Demographic and Health Survey (DHS) Data.

**PERCENT CHILDREN NOT EATING OTHER FRUITS & VEGETABLES ACROSS 19 PROVINCES IN CAMBODIA**

| 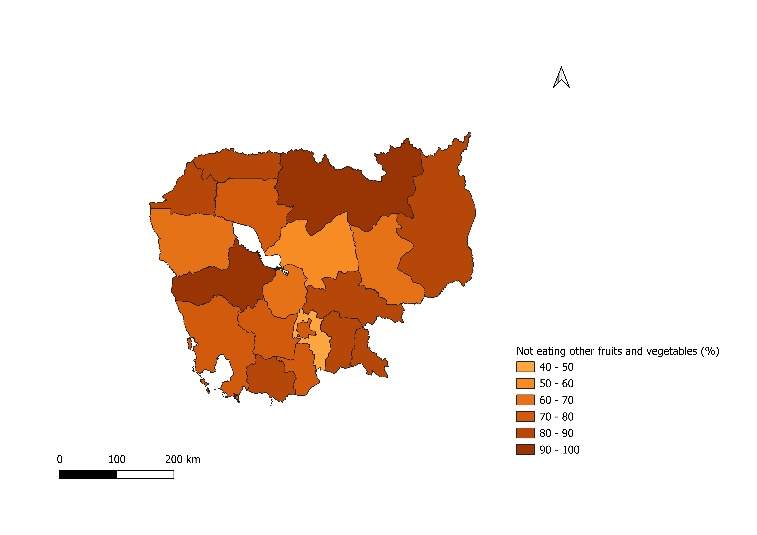  2005 | 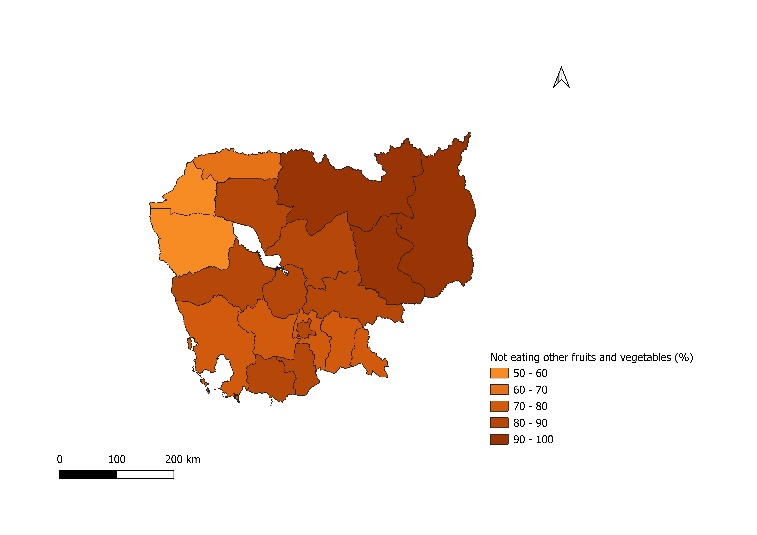  2010 |
| --- | --- |
| 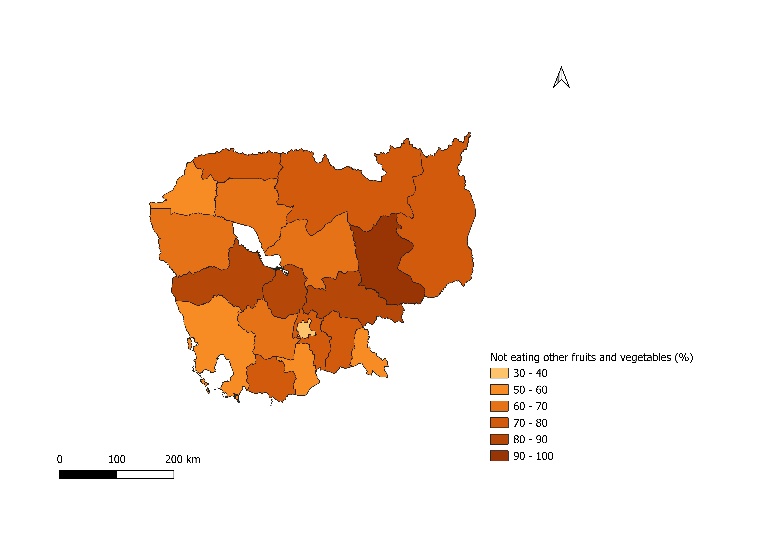  2014 | 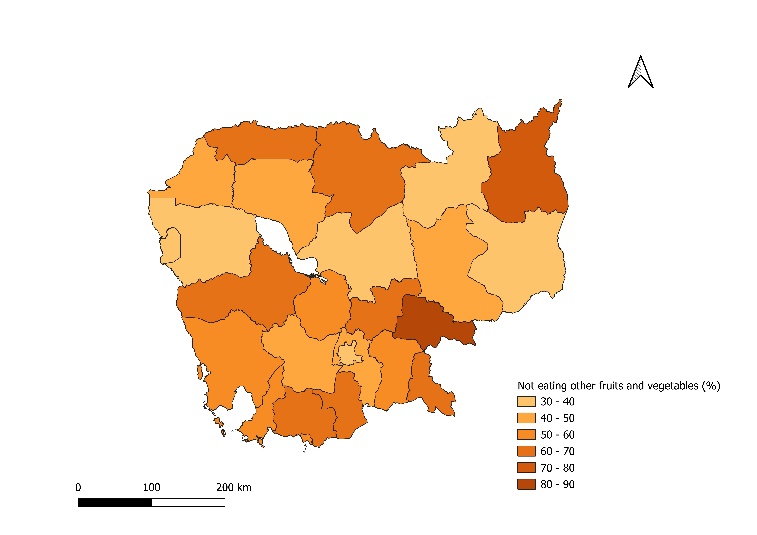  2021-22 |

**Appendix 1 Figure 5|** Geographic distribution of percent children not eating other fruits & vegetables across 19 provinces in Cambodia.

Source: Authors estimation using Demographic and Health Survey (DHS) Data.

**PERCENT CHILDREN NOT EATING EGGS ACROSS 19 PROVINCES IN CAMBODIA**

| 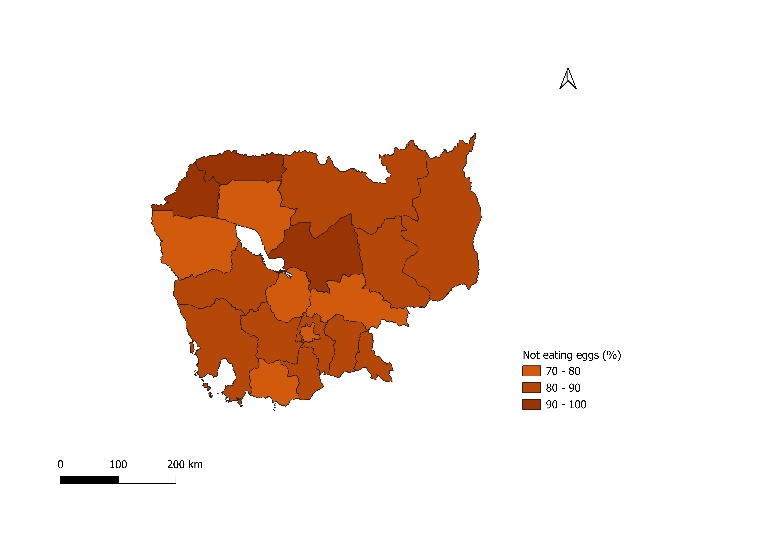  2005 | 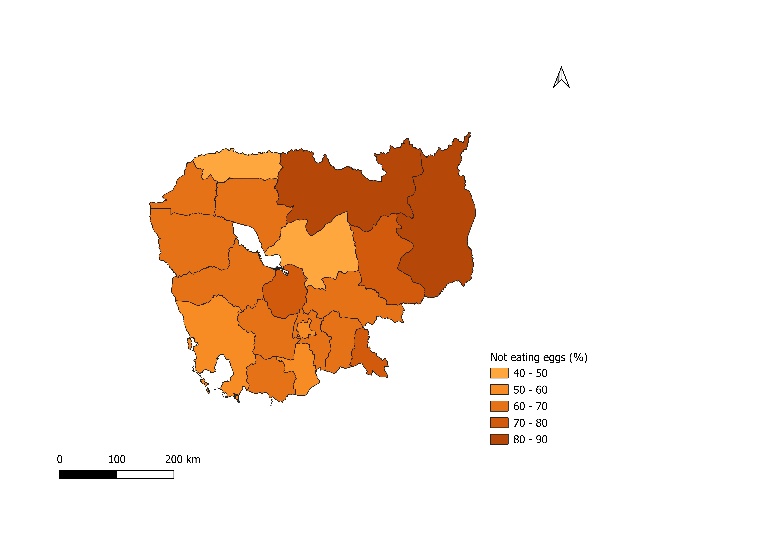  2010 |
| --- | --- |
| 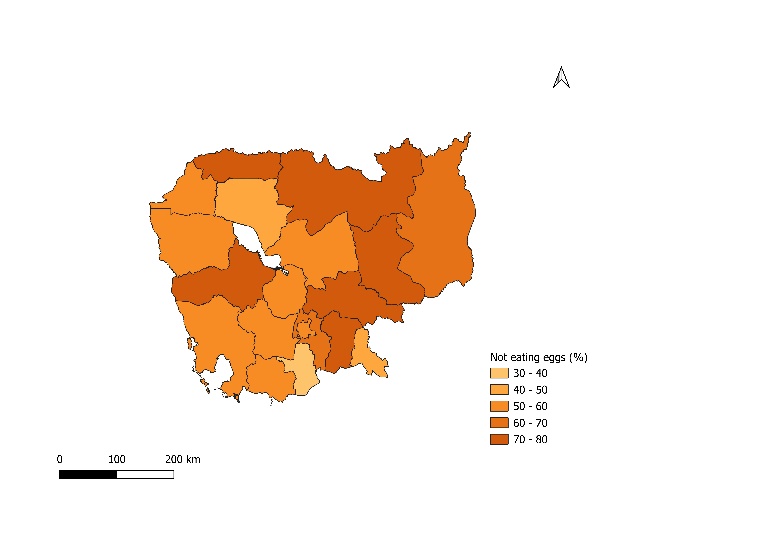  2014 | 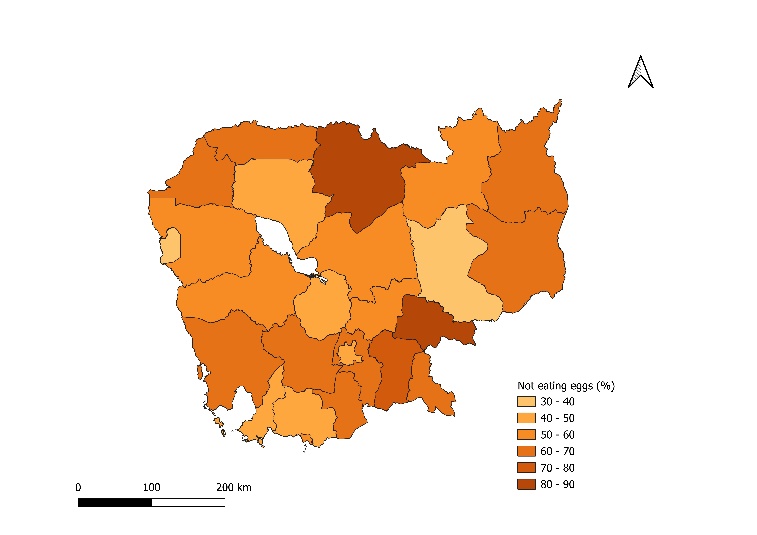  2021-22 |

**Appendix 1 Figure 6**| Geographic distribution of percent children not eating eggs across 19 provinces in Cambodia, 2005.

Source: Authors estimation using Demographic and Health Survey (DHS) Data.

**PERCENT CHILDREN NOT EATING FLESH FOODS ACROSS 19 PROVINCES IN CAMBODIA.**

| 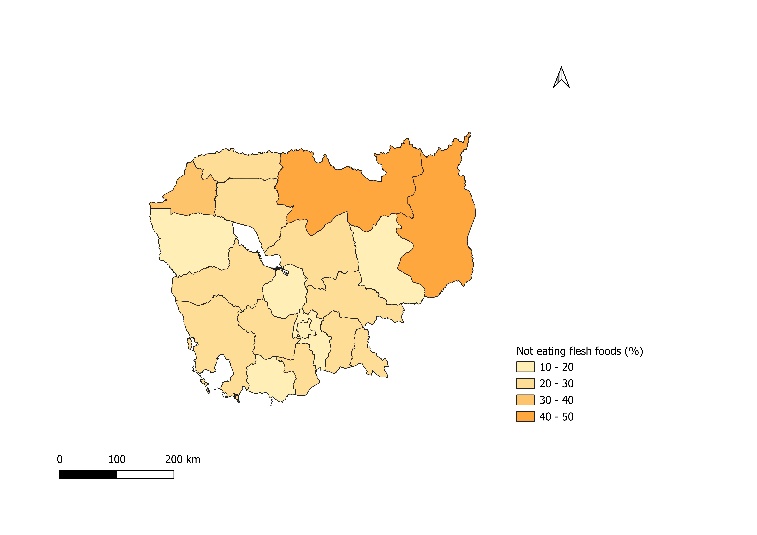  2005 | 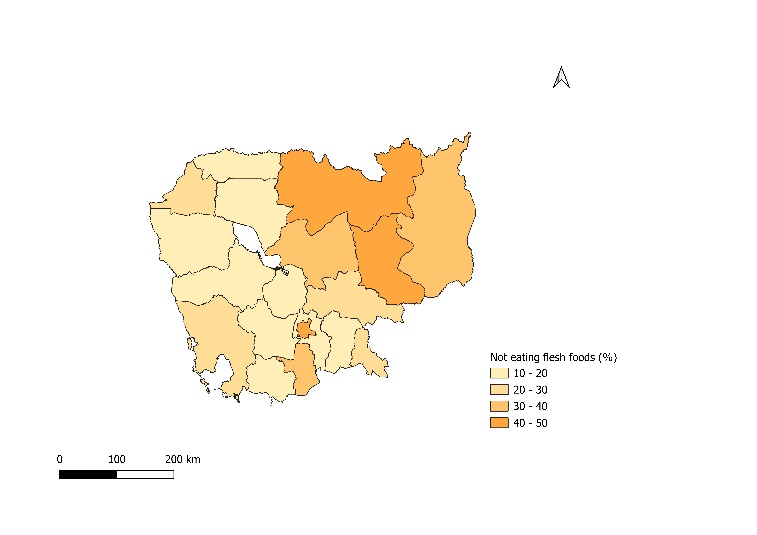  2010 |
| --- | --- |
| 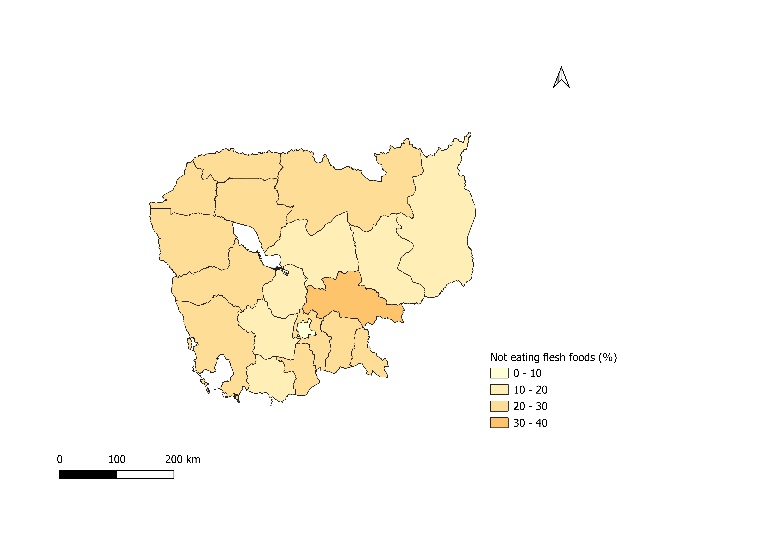  2014 | 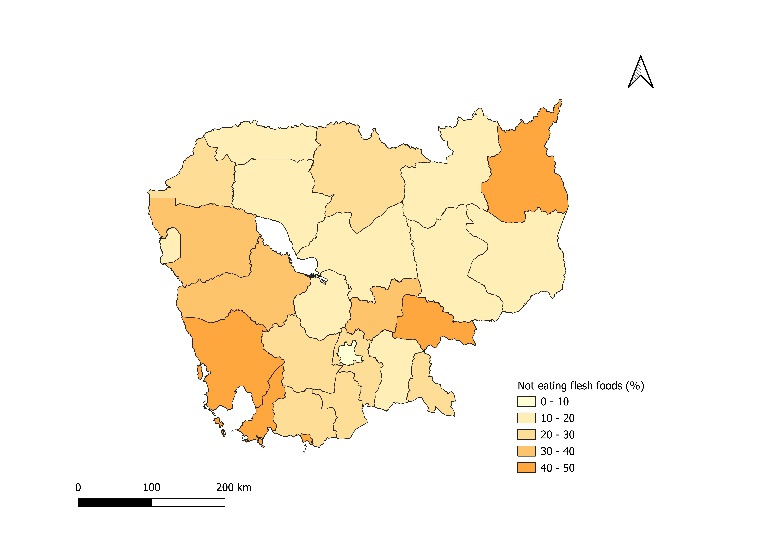  2021-22 |

**Appendix 1 Figure 7**| Geographic distribution of percent children not eating Flesh foods across 19 provinces in Cambodia, 2005.

Source: Authors estimation using Demographic and Health Survey (DHS) Data.

**PERCENT CHILDREN NOT EATING LEGUMES & NUTS ACROSS 19 PROVINCES IN CAMBODIA**

| 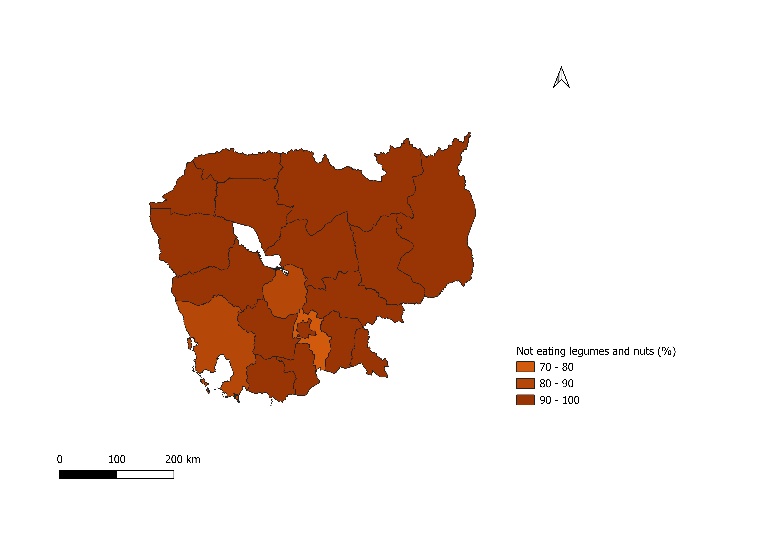  2005 | 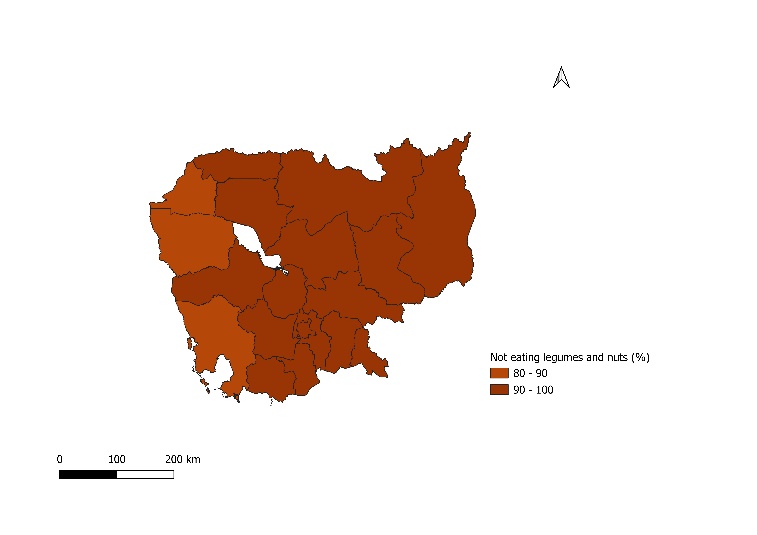  2010 |
| --- | --- |
| 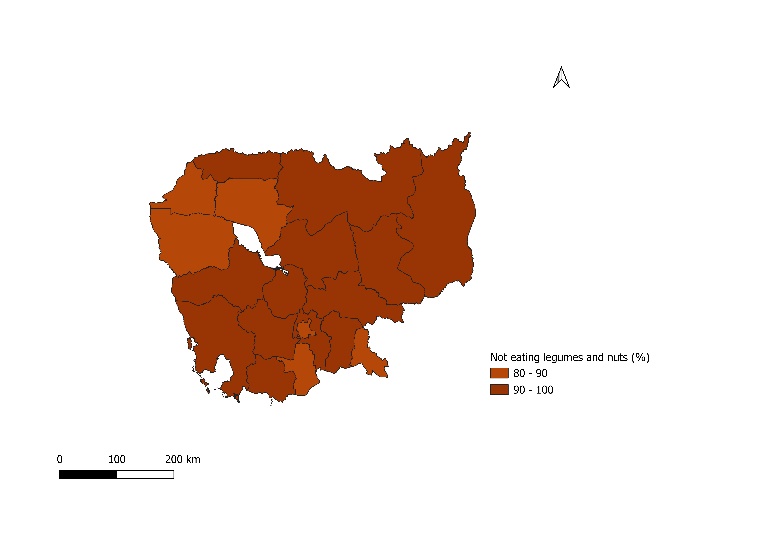  2014 | 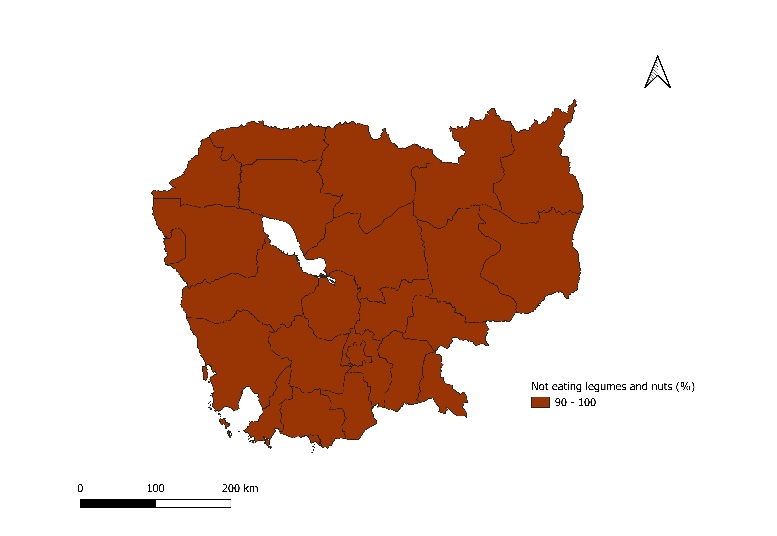  2021-22 |

**Appendix 1 Figure 8** | Geographic distribution of percent children not eating legumes & Nuts across 19 provinces in Cambodia.

Source: Authors estimation using Demographic and Health Survey (DHS) Data.

**APPENDIX 2**

**Appendix 2 TABLE 1** | Multinomial logistic regression results of inadequate Breastfeeding and associated factors among children aged 6–23 months in Cambodia: based on 2005–2021 DHS.

|  | **CDHS 2005** | **CDHS 2010** | **CDHS 2014** | **CDHS 2021** | **Conbined** |
| --- | --- | --- | --- | --- | --- |
| **Variables** | Odds Ratio^a^ [95% CI] | Odds Ratio^b^ [95% CI] | Odds Ratio^c^ [95% CI] | Odds Ratio^d^ [95% CI] | Odds Ratio^e^ [95% CI] |
| **Child Age** |  |  |  |  |  |
| 6-11months | 1.000 | 1.000 | 1.000 | 1.000 | 1.000 |
| 12-17months | 3.625 [2.332,5.636] | 3.138 [2.238,4.399] | 2.585 [1.85,3.612] | 1.989 [1.574,2.513] | 2.424 [2.09,2.811] |
| 18-23months | 20.689 [13.365,32.027] | 15.77 [11.078,22.448] | 18.549 [13.077,26.312] | 5.87 [4.578,7.526] | 11.196 [9.612,13.04] |
| **Child Sex** |  |  |  |  |  |
| Male | 1.000 | 1.000 | 1.000 | 1.000 | 1.000 |
| Female | 1.15 [0.889,1.486] | 1.364 [1.086,1.713] | 1.098 [0.865,1.393] | 0.983 [0.816,1.185] | 1.123 [1.008,1.251] |
| **Birth order** |  |  |  |  |  |
| First | 1.000 | 1.000 | 1.000 | 1.000 | 1.000 |
| Second or third | 0.581 [0.184,1.83] | 1.066 [0.105,10.785] | 3.285 [0.249,43.255] | 0.553 [0.482,0.635] | 2.261 [0.672,7.604] |
| Fourth or higher | 0.496 [0.156,1.579] | 0.884 [0.084,9.239] | 3.686 [0.27,50.302] | 0.426 [0.37,0.49] | 2.007 [0.588,6.849] |
| **Mother's age** |  |  |  |  |  |
| 15-19yrs | 1.000 | 1.000 | 1.000 | 1.000 | 1.000 |
| 20-34yrs | 0.884 [0.465,1.68] | 1.298 [0.723,2.331] | 0.955 [0.543,1.68] | 1.365 [0.862,2.159] | 1.157 [0.887,1.509] |
| 35-49yrs | 0.614 [0.289,1.303] | 1.484 [0.739,2.977] | 0.788 [0.387,1.604] | 1.104 [0.649,1.879] | 0.978 [0.714,1.34] |
| **Mother's Education** |  |  |  |  |  |
| No education | 1.000 | 1.000 | 1.000 | 1.000 | 1.000 |
| Primary | 1.555 [1.084,2.231] | 1.309 [0.905,1.893] | 0.895 [0.59,1.358] | 1.019 [0.727,1.43] | 1.185 [0.993,1.413] |
| Secondary | 1.676 [1.011,2.778] | 1.302 [0.835,2.029] | 0.784 [0.484,1.269] | 0.947 [0.655,1.37] | 1.133 [0.922,1.394] |
| Higher | 3.251 [0.642,16.448] | 1.945 [0.801,4.726] | 1.087 [0.499,2.367] | 1.122 [0.615,2.046] | 1.488 [1.017,2.176] |
| **Mother's Occupation** |  |  |  |  |  |
| Not working | 1.000 | 1.000 | 1.000 | 1.000 | 1.000 |
| White collar | 0.564 [0.167,1.899] | 1.449 [1.021,2.057] | 1.707 [1.184,2.462] | 1.386 [1.062,1.809] | 1.597 [1.348,1.892] |
| Agri worker | 0.241 [0.072,0.798] | 1.063 [0.738,1.531] | 1.214 [0.846,1.743] | 0.928 [0.679,1.269] | 1.058 [0.887,1.262] |
| Service/manual work | 0.391 [0.117,1.298] | 1.615 [1.069,2.44] | 1.496 [1.014,2.208] | 2.089 [1.599,2.729] | 1.718 [1.443,2.045] |
| **Marital Status** |  |  |  |  |  |
| Married | 1.000 | 1.000 | 1.000 | 1.000 | 1.000 |
| Not married | 0.853 [0.433,1.681] | 1.176 [0.655,2.112] | 2.182 [1.226,3.883] | 1.338 [0.665,2.691] | 1.271 [0.951,1.698] |
| **Preceding birth interval** |  |  |  |  |  |
| first birth | 1.000 | 1.000 | 1.000 | 1.000 | 1.000 |
| <36months | 7.216 [2.064,25.223] | 0.806 [0.079,8.201] | 0.239 [0.017,3.199] | 6.933 [2.108,22.802] | 0.346 [0.102,1.17] |
| >=36months | 5.357 [1.538,18.656] | 0.695 [0.068,7.049] | 0.256 [0.019,3.398] | 5.277 [1.61,17.298] | 0.344 [0.102,1.159] |
| **Media exposure** |  |  |  |  |  |
| No | 1.000 | 1.000 | 1.000 | 1.000 | 1.000 |
| Partial | 0.877 [0.596,1.292] | 1.029 [0.708,1.496] | 1.259 [0.87,1.822] | 0.77 [0.626,0.947] | 0.886 [0.766,1.024] |
| Full | 1.12 [0.672,1.867] | 0.955 [0.609,1.497] | 1.245 [0.769,2.015] | 1.254 [0.776,2.027] | 0.921 [0.75,1.13] |
| **Wanted Pregnancy** |  |  |  |  |  |
| Wanted | 1.000 | 1.000 | 1.000 | 1.000 | 1.000 |
| Unwanted/Mistimed | 0.873 [0.638,1.195] | 1.092 [0.78,1.528] | 0.843 [0.59,1.204] | 0.931 [0.713,1.215] | 0.912 [0.783,1.061] |
| **ANC Visit during pregnancy** |  |  |  |  |  |
| <4 visit | 1.000 | 1.000 | 1.000 | 1.000 | 1.000 |
| >=4 visit | 1.174 [0.859,1.605] | 0.81 [0.617,1.062] | 0.989 [0.718,1.361] | 1.041 [0.79,1.371] | 0.98 [0.851,1.127] |
| **Place of delivery** |  |  |  |  |  |
| Health facility-Pub | 1.000 | 1.000 | 1.000 | 1.000 | 1.000 |
| Health facility-Pri | 1.043 [0.583,1.865] | 1.174 [0.813,1.696] | 1.461 [1.04,2.054] | 1.569 [1.172,2.1] | 1.411 [1.187,1.678] |
| Home/Other | 0.743 [0.511,1.08] | 0.885 [0.663,1.181] | 0.64 [0.413,0.992] | 0.825 [0.495,1.377] | 0.91 [0.767,1.081] |
| **Visited health facility in last 12 months** |  |  |  |  |  |
| No | 1.000 | 1.000 | 1.000 | 1.000 | 1.000 |
| Yes | 0.889 [0.681,1.16] | 0.937 [0.741,1.186] | 0.977 [0.757,1.263] | 0.912 [0.748,1.111] | 0.913 [0.816,1.021] |
| **Father's education** |  |  |  |  |  |
| no education | 1.000 | 1.000 | 1.000 | 1.000 | 1.000 |
| primary | 0.995 [0.67,1.475] | 0.95 [0.641,1.407] | 1.127 [0.715,1.777] | 1.022 [0.722,1.447] | 1.012 [0.84,1.22] |
| secondary | 0.839 [0.53,1.33] | 1.083 [0.704,1.666] | 1.292 [0.796,2.096] | 0.975 [0.681,1.396] | 1.022 [0.837,1.249] |
| higher | 1.441 [0.55,3.777] | 1.254 [0.624,2.519] | 0.98 [0.487,1.971] | 0.972 [0.567,1.666] | 1.083 [0.787,1.489] |
| **Father's occupation** |  |  |  |  |  |
| Not working | 1.000 | 1.000 | 1.000 | 1.000 | 1.000 |
| white collar | 2.911 [0.655,12.929] | 0.887 [0.228,3.443] | 0.536 [0.186,1.546] | 0.963 [0.574,1.613] | 1.07 [0.757,1.513] |
| Agri worker | 2.501 [0.58,10.78] | 0.657 [0.173,2.49] | 0.387 [0.129,1.159] | 0.713 [0.435,1.168] | 0.793 [0.567,1.109] |
| service/manual work | 2.841 [0.663,12.179] | 0.747 [0.197,2.834] | 0.41 [0.139,1.205] | 1.033 [0.649,1.643] | 0.978 [0.707,1.353] |
| **Place of residence** |  |  |  |  |  |
| Urban | 1.000 | 1.000 | 1.000 | 1.000 | 1.000 |
| Rural | 0.67 [0.476,0.942] | 0.788 [0.558,1.113] | 0.704 [0.483,1.025] | 0.797 [0.631,1.006] | 0.747 [0.645,0.864] |
| **Wealth Index** |  |  |  |  |  |
| Poorest | 1.000 | 1.000 | 1.000 | 1.000 | 1.000 |
| Poorer | 0.59 [0.395,0.883] | 0.829 [0.562,1.222] | 0.986 [0.664,1.465] | 1.279 [0.947,1.728] | 0.976 [0.818,1.164] |
| Middle | 0.901 [0.596,1.363] | 1.111 [0.743,1.661] | 0.973 [0.62,1.526] | 1.62 [1.184,2.218] | 1.246 [1.034,1.5] |
| Richer | 0.728 [0.457,1.162] | 0.917 [0.586,1.435] | 0.793 [0.489,1.285] | 1.897 [1.361,2.646] | 1.15 [0.941,1.406] |
| Richest | 1.034 [0.59,1.813] | 2.24 [1.311,3.828] | 1.784 [1.02,3.119] | 3.715 [2.42,5.705] | 2.01 [1.578,2.56] |
| **Household Size** |  |  |  |  |  |
| <=4 | 1.000 | 1.000 | 1.000 | 1.000 | 1.000 |
| 5-9 | 0.914 [0.672,1.243] | 0.638 [0.489,0.832] | 0.994 [0.752,1.313] | 1.041 [0.851,1.273] | 0.896 [0.792,1.013] |
| >=10 | 1.26 [0.756,2.101] | 0.878 [0.552,1.396] | 1.117 [0.666,1.872] | 1.129 [0.655,1.944] | 1.072 [0.843,1.364] |
| **Sex of head of household** |  |  |  |  |  |
| Male | 1.000 | 1.000 | 1.000 | 1.000 | 1.000 |
| Female | 1.054 [0.7,1.587] | 1.143 [0.857,1.525] | 1.171 [0.878,1.562] | 0.974 [0.785,1.21] | 1.069 [0.935,1.223] |
| **Year of Survey** |  |  |  |  |  |
| 2005 |  |  |  |  | 1.000 |
| 2010 |  |  |  |  | 1.572 [1.301,1.901] |
| 2014 |  |  |  |  | 1.938 [1.574,2.387] |
| 2021 |  |  |  |  | 3.737 [2.959,4.720] |

a= Cambodia DHS survey year 2005, b= Cambodia DHS survey year 2010, c= Cambodia DHS survey year 2014, d= Cambodia DHS survey year 2021, e= Cambodia DHS survey year 2005-2021

Source: Authors estimation using Demographic and Health Survey (DHS) Data.

**Appendix 2 TABLE 2** | Multinomial logistic regression results of inadequate consumption of Dairy Products and associated factors among children aged 6–23 months in Cambodia: based on 2005–2021 DHS

|  | **CDHS 2005** | **CDHS 2010** | **CDHS 2014** | **CDHS 2021** | **Combined** |
| --- | --- | --- | --- | --- | --- |
| **Sample characteristics** | Odds Ratio^a^ [95% CI] | Odds Ratio^b^ [95% CI] | Odds Ratio^c^ [95% CI] | Odds Ratio^d^ [95% CI] | Odds Ratioe [95% CI] |
| **Child Age** |  |  |  |  |  |
| 6-11months | 1.000 | 1.000 | 1.000 | 1.000 | 1.000 |
| 12-17months | 0.616 [0.448,0.846] | 0.709 [0.514,0.977] | 0.463 [0.347,0.619] | 0.517 [0.412,0.648] | 0.542 [0.472,0.623] |
| 18-23months | 0.571 [0.411,0.795] | 0.749 [0.534,1.049] | 0.374 [0.281,0.498] | 0.331 [0.261,0.418] | 0.432 [0.375,0.497] |
| **Child Sex** |  |  |  |  |  |
| Male | 1.000 | 1.000 | 1.000 | 1.000 | 1.000 |
| Female | 0.996 [0.772,1.284] | 0.969 [0.747,1.256] | 1.093 [0.873,1.367] | 0.94 [0.782,1.13] | 1 [0.895,1.117] |
| **Birth order** |  |  |  |  |  |
| First | 1.000 | 1.000 | 1.000 | 1.000 | 1.000 |
| Second or third | 0.277 [0.026,2.932] | 0.167 [0.005,4.826] | 0.365 [0.043,3.075] | 0.19 [0.054,0.661] | 0.165 [0.048,0.566] |
| Fourth or higher | 0.26 [0.024,2.823] | 0.172 [0.005,5.119] | 0.477 [0.054,4.186] | 0.257 [0.072,0.908] | 0.196 [0.056,0.682] |
| **Mother's age** |  |  |  |  |  |
| 15-19yrs | 1.000 | 1.000 | 1.000 | 1.000 | 1.000 |
| 20-34yrs | 0.922 [0.492,1.728] | 0.603 [0.308,1.179] | 0.638 [0.372,1.096] | 0.84 [0.539,1.309] | 0.746 [0.569,0.979] |
| 35-49yrs | 0.706 [0.341,1.459] | 0.653 [0.293,1.453] | 0.537 [0.274,1.052] | 0.797 [0.477,1.332] | 0.666 [0.483,0.917] |
| **Mother's Education** |  |  |  |  |  |
| No education | 1.000 | 1.000 | 1.000 | 1.000 | 1.000 |
| Primary | 0.894 [0.616,1.298] | 0.684 [0.414,1.129] | 0.895 [0.586,1.367] | 0.864 [0.619,1.207] | 0.846 [0.697,1.027] |
| Secondary | 0.676 [0.417,1.096] | 0.39 [0.226,0.673] | 1.023 [0.638,1.64] | 0.76 [0.528,1.093] | 0.687 [0.553,0.854] |
| Higher | 0.329 [0.066,1.624] | 0.138 [0.051,0.371] | 0.612 [0.287,1.306] | 0.592 [0.321,1.093] | 0.436 [0.292,0.651] |
| **Mother's Occupation** |  |  |  |  |  |
| Not working | 1.000 | 1.000 | 1.000 | 1.000 | 1.000 |
| White collar | 2.476 [0.746,8.215] | 0.703 [0.496,0.995] | 0.601 [0.433,0.835] | 0.755 [0.584,0.977] | 0.672 [0.569,0.793] |
| Agri worker | 3.311 [1.007,10.88] | 1.626 [1.042,2.537] | 1.06 [0.751,1.496] | 1.1 [0.812,1.491] | 1.097 [0.913,1.317] |
| Service/manual work | 3.096 [0.945,10.137] | 0.567 [0.372,0.864] | 0.516 [0.364,0.731] | 0.393 [0.302,0.512] | 0.555 [0.467,0.659] |
| **Marital Status** |  |  |  |  |  |
| Married | 1.000 | 1.000 | 1.000 | 1.000 | 1.000 |
| Not married | 0.871 [0.438,1.73] | 1.11 [0.56,2.203] | 0.954 [0.548,1.661] | 0.97 [0.49,1.919] | 1.009 [0.742,1.371] |
| **Preceding birth interval** |  |  |  |  |  |
| first birth | 1.000 | 1.000 | 1.000 | 1.000 | 1.000 |
| <36months | 5.785 [0.541,61.789] | 6.496 [0.223,188.533] | 4.858 [0.566,41.637] | 0.899 [0.266,3.033] | 8.589 [2.496,29.552] |
| >=36months | 5.248 [0.495,55.608] | 5.41 [0.186,156.852] | 3.572 [0.421,30.314] | 0.694 [0.206,2.334] | 7.109 [2.073,24.373] |
| **Media exposure** |  |  |  |  |  |
| No | 1.000 | 1.000 | 1.000 | 1.000 | 1.000 |
| Partial | 0.727 [0.466,1.135] | 1.113 [0.658,1.882] | 0.979 [0.676,1.418] | 1.295 [1.058,1.586] | 1.138 [0.976,1.327] |
| Full | 0.494 [0.285,0.857] | 1.378 [0.766,2.476] | 0.729 [0.46,1.156] | 0.811 [0.499,1.318] | 0.97 [0.784,1.198] |
| **Wanted Pregnancy** |  |  |  |  |  |
| Wanted | 1.000 | 1.000 | 1.000 | 1.000 | 1.000 |
| Unwanted/Mistimed | 1.146 [0.838,1.568] | 1.078 [0.727,1.598] | 1.257 [0.891,1.771] | 1.068 [0.823,1.385] | 1.09 [0.933,1.274] |
| **ANC Visit during pregnancy** |  |  |  |  |  |
| <4 visit | 1.000 | 1.000 | 1.000 | 1.000 | 1.000 |
| >=4 visit | 0.921 [0.678,1.249] | 1.037 [0.756,1.423] | 1.236 [0.908,1.682] | 0.952 [0.728,1.244] | 1.01 [0.873,1.168] |
| **Place of delivery** |  |  |  |  |  |
| Health facility-Pub | 1.000 | 1.000 | 1.000 | 1.000 | 1.000 |
| Health facility-Pri | 0.911 [0.531,1.565] | 0.698 [0.482,1.011] | 0.66 [0.482,0.902] | 0.59 [0.439,0.792] | 0.671 [0.564,0.797] |
| Home/Other | 0.969 [0.669,1.404] | 1.091 [0.767,1.552] | 1.136 [0.743,1.737] | 1.234 [0.743,2.048] | 1.035 [0.862,1.242] |
| **Visited health facility in last 12 months** |  |  |  |  |  |
| No | 1.000 | 1.000 | 1.000 | 1.000 | 1.000 |
| Yes | 1.037 [0.793,1.357] | 1.06 [0.806,1.393] | 1.069 [0.839,1.363] | 1.065 [0.876,1.294] | 1.039 [0.925,1.166] |
| **Father's education** |  |  |  |  |  |
| no education | 1.000 | 1.000 | 1.000 | 1.000 | 1.000 |
| primary | 0.863 [0.565,1.318] | 1.076 [0.63,1.838] | 1.572 [1.018,2.429] | 1.039 [0.739,1.462] | 1.063 [0.869,1.301] |
| secondary | 0.706 [0.442,1.129] | 0.989 [0.573,1.706] | 1.051 [0.67,1.649] | 0.886 [0.624,1.259] | 0.896 [0.726,1.106] |
| higher | 0.526 [0.218,1.268] | 1.062 [0.503,2.244] | 0.958 [0.503,1.825] | 0.801 [0.469,1.37] | 0.86 [0.624,1.185] |
| **Father's occupation** |  |  |  |  |  |
| Not working | 1.000 | 1.000 | 1.000 | 1.000 | 1.000 |
| white collar | 0.766 [0.261,2.244] | 3.624 [1.079,12.165] | 0.968 [0.332,2.823] | 0.806 [0.489,1.33] | 0.839 [0.596,1.182] |
| agri worker | 1.194 [0.418,3.411] | 4.965 [1.502,16.41] | 1.258 [0.417,3.792] | 1.147 [0.713,1.846] | 1.261 [0.903,1.759] |
| service/manual work | 1.087 [0.381,3.096] | 5.562 [1.684,18.373] | 1.079 [0.364,3.196] | 0.86 [0.548,1.348] | 1.077 [0.781,1.485] |
| **Place of residence** |  |  |  |  |  |
| Urban | 1.000 | 1.000 | 1.000 | 1.000 | 1.000 |
| Rural | 1.185 [0.839,1.675] | 1.169 [0.816,1.674] | 1.021 [0.735,1.419] | 1.237 [0.989,1.549] | 1.229 [1.061,1.424] |
| **Wealth Index** |  |  |  |  |  |
| Poorest | 1.000 | 1.000 | 1.000 | 1.000 | 1.000 |
| Poorer | 0.687 [0.443,1.065] | 1.13 [0.586,2.179] | 0.548 [0.362,0.831] | 0.649 [0.486,0.866] | 0.669 [0.55,0.813] |
| Middle | 0.628 [0.398,0.99] | 0.484 [0.271,0.866] | 0.506 [0.321,0.798] | 0.5 [0.368,0.678] | 0.541 [0.442,0.663] |
| Richer | 0.587 [0.357,0.965] | 0.291 [0.161,0.524] | 0.394 [0.246,0.63] | 0.348 [0.252,0.483] | 0.418 [0.338,0.516] |
| Richest | 0.288 [0.163,0.509] | 0.14 [0.072,0.271] | 0.173 [0.101,0.297] | 0.252 [0.164,0.386] | 0.219 [0.17,0.283] |
| **Household Size** |  |  |  |  |  |
| <=4 | 1.000 | 1.000 | 1.000 | 1.000 | 1.000 |
| 5-9 | 0.931 [0.681,1.273] | 0.929 [0.681,1.268] | 0.859 [0.661,1.118] | 0.84 [0.688,1.025] | 0.854 [0.752,0.97] |
| >=10 | 0.898 [0.536,1.503] | 0.787 [0.473,1.307] | 0.654 [0.407,1.052] | 0.744 [0.435,1.272] | 0.721 [0.565,0.92] |
| **Sex of head of household** |  |  |  |  |  |
| Male | 1.000 | 1.000 | 1.000 | 1.000 | 1.000 |
| Female | 0.955 [0.658,1.384] | 0.861 [0.625,1.187] | 0.95 [0.726,1.243] | 0.946 [0.766,1.17] | 0.952 [0.831,1.09] |
| **Year of Survey** |  |  |  |  |  |
| 2005 |  |  |  |  | 1.000 |
| 2010 |  |  |  |  | 1.032 [0.839,1.268] |
| 2014 |  |  |  |  | 0.473 [0.381,0.587] |
| 2021 |  |  |  |  | 0.21 [0.165,0.267] |

a= Cambodia DHS survey year 2005, b= Cambodia DHS survey year 2010, c= Cambodia DHS survey year 2014, d= Cambodia DHS survey year 2021, e= Cambodia DHS survey year 2005-2021

Source: Authors estimation using Demographic and Health Survey (DHS) Data.

**Appendix 2 TABLE 3** | Multinomial logistic regression results of inadequate consumption of Grains, roots, and tubers and associated factors among children aged 6–23 months in Cambodia: based on 2005–2021 DHS.

|  | **CDHS 2004** | **CDHS 2010** | **CDHS 2014** | **CDHS 2021** | **Combined** |
| --- | --- | --- | --- | --- | --- |
| **Sample characteristics** | Odds Ratio^a^ [95% CI] | Odds Ratio^b^ [95% CI] | Odds Ratio^c^ [95% CI] | Odds Ratio^d^ [95% CI] | Odds Ratio^e^ [95% CI] |
| **Child Age** |  |  |  |  |  |
| 6-11months | 1.000 | 1.000 | 1.000 | 1.000 | 1.000 |
| 12-17months | 0.142 [0.104,0.195] | 0.239 [0.161,0.355] | 0.269 [0.178,0.406] | 0.63 [0.432,0.92] | 0.256 [0.214,0.305] |
| 18-23months | 0.079 [0.053,0.118] | 0.261 [0.174,0.392] | 0.236 [0.155,0.359] | 0.403 [0.263,0.615] | 0.191 [0.156,0.233] |
| **Child Sex** |  |  |  |  |  |
| Male | 1.000 | 1.000 | 1.000 | 1.000 | 1.000 |
| Female | 1.043 [0.811,1.341] | 1.174 [0.862,1.6] | 0.995 [0.726,1.364] | 1.107 [0.799,1.534] | 1.073 [0.93,1.238] |
| **Birth order** |  |  |  |  |  |
| First | 1.000 | 1.000 | 1.000 | 1.000 | 1.000 |
| Second or third | 1.954 [0.187,20.322] | 1.328 [1.075,1.670] | 1.815 [0.931,3.539] | 0.776 [0.592,1.017] | 0.819 [0.162,4.14] |
| Fourth or higher | 1.348 [0.126,14.343] | 0.727 [0.436,0.954] | 1.854 [0.924,3.718] | 0.646 [0.47,0.888] | 0.695 [0.135,3.57] |
| **Mother's age** |  |  |  |  |  |
| 15-19yrs | 1.000 | 1.000 | 1.000 | 1.000 | 1.000 |
| 20-34yrs | 1.063 [0.577,1.957] | 1.009 [0.508,2.001] | 1.18 [0.559,2.488] | 1.019 [0.493,2.105] | 1.118 [0.804,1.554] |
| 35-49yrs | 1.406 [0.69,2.864] | 0.956 [0.408,2.234] | 1.486 [0.598,3.688] | 1.155 [0.487,2.734] | 1.27 [0.856,1.883] |
| **Mother's Education** |  |  |  |  |  |
| No education | 1.000 | 1.000 | 1.000 | 1.000 | 1.000 |
| Primary | 0.743 [0.534,1.035] | 1.25 [0.799,1.955] | 0.726 [0.443,1.192] | 0.979 [0.546,1.758] | 0.838 [0.682,1.031] |
| Secondary | 0.958 [0.594,1.545] | 0.8 [0.439,1.457] | 0.581 [0.321,1.052] | 0.87 [0.454,1.667] | 0.753 [0.578,0.981] |
| Higher | 2.341 [0.443,12.365] | 0.396 [0.072,2.165] | 0.957 [0.348,2.633] | 1.253 [0.446,3.521] | 0.943 [0.545,1.633] |
| **Mother's Occupation** |  |  |  |  |  |
| Not working | 1.000 | 1.000 | 1.000 | 1.000 | 1.000 |
| White collar | 1.011 [0.224,4.553] | 0.988 [0.607,1.609] | 0.55 [0.335,0.904] | 0.739 [0.458,1.193] | 0.771 [0.602,0.986] |
| Agri worker | 0.888 [0.201,3.919] | 0.795 [0.498,1.269] | 0.407 [0.261,0.636] | 0.764 [0.448,1.3] | 0.626 [0.496,0.79] |
| Service/manual work | 1.344 [0.304,5.928] | 0.737 [0.397,1.37] | 0.853 [0.52,1.399] | 0.747 [0.459,1.215] | 0.89 [0.7,1.132] |
| **Marital Status** |  |  |  |  |  |
| Married | 1.000 | 1.000 | 1.000 | 1.000 | 1.000 |
| Not married | 0.865 [0.417,1.791] | 0.78 [0.317,1.918] | 0.352 [0.104,1.195] | 1.308 [0.399,4.285] | 0.691 [0.447,1.069] |
| **Preceding birth interval** |  |  |  |  |  |
| first birth | 1.000 | 1.000 | 1.000 | 1.000 | 1.000 |
| <36months | 0.467 [0.044,4.886] | 3.200 [0.620,16.516] | 1.124 [0.693,1.823] | 1.835 [0.599,5.614] | 1.221 [0.24,6.202] |
| >=36months | 0.488 [0.046,5.078] | 2.643 [0.515,13.539] | 1.209 [0.526,2.776] | 1.361 [0.446,4.151] | 1.202 [0.237,6.085] |
| **Media exposure** |  |  |  |  |  |
| No | 1.000 | 1.000 | 1.000 | 1.000 | 1.000 |
| Partial | 0.905 [0.628,1.304] | 0.663 [0.424,1.035] | 1.295 [0.819,2.046] | 0.948 [0.648,1.385] | 0.973 [0.802,1.181] |
| Full | 0.756 [0.441,1.294] | 0.615 [0.34,1.114] | 1.184 [0.616,2.275] | 2.784 [1.461,5.305] | 0.996 [0.752,1.32] |
| **Wanted Pregnancy** |  |  |  |  |  |
| Wanted | 1.000 | 1.000 | 1.000 | 1.000 | 1.000 |
| Unwanted/Mistimed | 1.173 [0.875,1.571] | 1.142 [0.746,1.748] | 0.876 [0.546,1.405] | 0.994 [0.631,1.565] | 1.109 [0.922,1.335] |
| **ANC Visit during pregnancy** |  |  |  |  |  |
| <4 visit | 1.000 | 1.000 | 1.000 | 1.000 | 1.000 |
| >=4 visit | 0.943 [0.686,1.297] | 1.143 [0.788,1.657] | 0.852 [0.568,1.278] | 0.662 [0.433,1.013] | 0.86 [0.717,1.032] |
| **Place of delivery** |  |  |  |  |  |
| Health facility-Pub | 1.000 | 1.000 | 1.000 | 1.000 | 1.000 |
| Health facility-Pri | 1.242 [0.66,2.337] | 0.827 [0.445,1.537] | 1.093 [0.675,1.768] | 1.124 [0.692,1.825] | 1.04 [0.8,1.352] |
| Home/Other | 0.716 [0.488,1.051] | 1.127 [0.765,1.661] | 0.853 [0.493,1.476] | 1.208 [0.524,2.785] | 0.917 [0.737,1.141] |
| **Visited health facility in last 12 months** |  |  |  |  |  |
| No | 1.000 | 1.000 | 1.000 | 1.000 | 1.000 |
| Yes | 1.072 [0.824,1.396] | 0.681 [0.487,0.954] | 0.957 [0.68,1.348] | 0.572 [0.406,0.805] | 0.826 [0.711,0.959] |
| **Father's education** |  |  |  |  |  |
| no education | 1.000 | 1.000 | 1.000 | 1.000 | 1.000 |
| primary | 0.907 [0.631,1.304] | 0.616 [0.389,0.976] | 1.251 [0.701,2.232] | 1.815 [0.931,3.539] | 0.956 [0.763,1.197] |
| secondary | 1.052 [0.684,1.615] | 0.651 [0.386,1.098] | 1.058 [0.558,2.006] | 1.854 [0.924,3.718] | 0.98 [0.76,1.263] |
| higher | 1.736 [0.625,4.821] | 0.588 [0.208,1.657] | 1.265 [0.477,3.356] | 1.392 [0.503,3.848] | 1.016 [0.641,1.611] |
| **Father's occupation** |  |  |  |  |  |
| Not working | 1.000 | 1.000 | 1.000 | 1.000 | 1.000 |
| white collar | 0.271 [0.094,0.779] | 1.558 [0.338,7.165] | 0.446 [0.109,1.828] | 0.697 [0.322,1.51] | 0.561 [0.353,0.893] |
| Agri worker | 0.413 [0.153,1.11] | 1.342 [0.304,5.907] | 0.602 [0.139,2.602] | 0.653 [0.318,1.339] | 0.631 [0.406,0.979] |
| service/manual work | 0.383 [0.14,1.042] | 1.061 [0.24,4.673] | 0.488 [0.115,2.068] | 0.624 [0.319,1.221] | 0.542 [0.352,0.835] |
| **Place of residence** |  |  |  |  |  |
| Urban | 1.000 | 1.000 | 1.000 | 1.000 | 1.000 |
| Rural | 0.965 [0.67,1.39] | 0.821 [0.498,1.353] | 0.973 [0.6,1.577] | 1.47 [0.97,2.229] | 1.041 [0.848,1.277] |
| **Wealth Index** |  |  |  |  |  |
| Poorest | 1.000 | 1.000 | 1.000 | 1.000 | 1.000 |
| Poorer | 1.423 [0.981,2.064] | 1.375 [0.847,2.231] | 0.784 [0.476,1.291] | 0.797 [0.458,1.387] | 1.11 [0.89,1.384] |
| Middle | 1.327 [0.882,1.997] | 1.217 [0.721,2.055] | 0.645 [0.355,1.17] | 1.17 [0.687,1.993] | 1.108 [0.872,1.408] |
| Richer | 1.172 [0.726,1.892] | 1.151 [0.633,2.093] | 0.824 [0.452,1.501] | 1.191 [0.674,2.104] | 1.067 [0.818,1.39] |
| Richest | 1.023 [0.56,1.87] | 1.275 [0.605,2.684] | 0.932 [0.46,1.888] | 1.249 [0.594,2.63] | 1.155 [0.832,1.605] |
| **Household Size** |  |  |  |  |  |
| <=4 | 1.000 | 1.000 | 1.000 | 1.000 | 1.000 |
| 5-9 | 1.113 [0.812,1.525] | 0.849 [0.587,1.229] | 1.104 [0.76,1.604] | 1.132 [0.794,1.616] | 1.028 [0.868,1.218] |
| >=10 | 0.932 [0.545,1.593] | 0.899 [0.488,1.654] | 0.923 [0.448,1.898] | 0.561 [0.161,1.944] | 0.889 [0.648,1.22] |
| **Sex of head of household** |  |  |  |  |  |
| Male | 1.000 | 1.000 | 1.000 | 1.000 | 1.000 |
| Female | 1.054 [0.73,1.522] | 1.1 [0.74,1.635] | 1.054 [0.7,1.587] | 0.964 [0.657,1.414] | 0.963 [0.797,1.163] |
| **Year of Survey** |  |  |  |  |  |
| 2005 |  |  |  |  | 1.000 |
| 2010 |  |  |  |  | 0.439 [0.348,0.553] |
| 2014 |  |  |  |  | 0.429 [0.329,0.559] |
| 2021 |  |  |  |  | 0.274 [0.201,0.374] |

a= Cambodia DHS survey year 2005, b= Cambodia DHS survey year 2010, c= Cambodia DHS survey year 2014, d= Cambodia DHS survey year 2021, e= Cambodia DHS survey year 2005-2021

Source: Authors estimation using Demographic and Health Survey (DHS) Data.

**Appendix 2 TABLE 4** | Multinomial logistic regression results of inadequate consumption of Vitamin A rich fruits and vegetables and associated factors among children aged 6–23 months in Cambodia: based on 2005–2021 DHS.

|  | **CDHS 2004** | **CDHS 2010** | **CDHS 2014** | **CDHS 2021** | **Combined** |
| --- | --- | --- | --- | --- | --- |
| **Variables** | Odds Ratio^a^ [95% CI] | Odds Ratio^b^ [95% CI] | Odds Ratio^c^ [95% CI] | Odds Ratio^d^ [95% CI] | Odds Ratio^e^ [95% CI] |
| **Child Age** |  |  |  |  |  |
| 6-11months | 1.000 | 1.000 | 1.000 | 1.000 | 1.000 |
| 12-17months | 0.335 [0.267,0.42] | 0.315 [0.25,0.395] | 0.353 [0.28,0.446] | 0.66 [0.532,0.818] | 0.415 [0.372,0.463] |
| 18-23months | 0.249 [0.196,0.317] | 0.257 [0.202,0.326] | 0.29 [0.229,0.367] | 0.697 [0.56,0.869] | 0.363 [0.324,0.406] |
| **Child Sex** |  |  |  |  |  |
| Male | 1.000 | 1.000 | 1.000 | 1.000 | 1.000 |
| Female | 0.958 [0.797,1.152] | 0.972 [0.808,1.168] | 1.111 [0.92,1.342] | 1.01 [0.847,1.205] | 1.012 [0.926,1.107] |
| **Birth order** |  |  |  |  |  |
| First | 1.000 | 1.000 | 1.000 | 1.000 | 1.000 |
| Second or third | 1.441 [0.165,12.578] | 1.173 [0.114,12.029] | 0.347 [0.033,3.646] | 0.784 [0.648,0.949] | 0.57 [0.181,1.794] |
| Fourth or higher | 1.146 [0.129,10.142] | 0.855 [0.081,8.92] | 0.245 [0.022,2.639] | 0.543 [0.439,0.671] | 0.488 [0.153,1.551] |
| **Mother's age** |  |  |  |  |  |
| 15-19yrs | 1.000 | 1.000 | 1.000 | 1.000 | 1.000 |
| 20-34yrs | 0.944 [0.593,1.502] | 0.823 [0.539,1.257] | 0.918 [0.603,1.399] | 1.05 [0.692,1.591] | 0.965 [0.781,1.191] |
| 35-49yrs | 0.966 [0.566,1.65] | 0.938 [0.558,1.578] | 0.808 [0.472,1.385] | 0.876 [0.539,1.425] | 0.907 [0.705,1.166] |
| **Mother's Education** |  |  |  |  |  |
| No education | 1.000 | 1.000 | 1.000 | 1.000 | 1.000 |
| Primary | 0.948 [0.743,1.211] | 0.988 [0.75,1.303] | 1.001 [0.728,1.376] | 0.99 [0.727,1.347] | 0.968 [0.845,1.109] |
| Secondary | 0.936 [0.649,1.351] | 0.675 [0.475,0.961] | 0.876 [0.604,1.271] | 0.847 [0.601,1.194] | 0.834 [0.705,0.987] |
| Higher | 3.149 [0.667,14.855] | 1.384 [0.627,3.058] | 1.335 [0.698,2.553] | 0.874 [0.49,1.559] | 1.068 [0.761,1.499] |
| **Mother's Occupation** |  |  |  |  |  |
| Not working | 1.000 | 1.000 | 1.000 | 1.000 | 1.000 |
| White collar | 1.985 [0.629,6.259] | 1.086 [0.807,1.461] | 0.97 [0.72,1.306] | 0.723 [0.558,0.937] | 0.872 [0.751,1.012] |
| Agri worker | 2.086 [0.671,6.482] | 0.912 [0.685,1.215] | 0.927 [0.71,1.211] | 0.805 [0.605,1.072] | 0.82 [0.71,0.946] |
| Service/manual work | 2.276 [0.731,7.079] | 1.114 [0.788,1.575] | 1.259 [0.923,1.718] | 0.979 [0.754,1.271] | 1.048 [0.902,1.218] |
| **Marital Status** |  |  |  |  |  |
| Married | 1.000 | 1.000 | 1.000 | 1.000 | 1.000 |
| Not married | 0.902 [0.544,1.496] | 1.196 [0.73,1.96] | 0.751 [0.46,1.228] | 1.604 [0.82,3.134] | 1.072 [0.835,1.375] |
| **Preceding birth interval** |  |  |  |  |  |
| first birth | 1.000 | 1.000 | 1.000 | 1.000 | 1.000 |
| <36months | 0.749 [0.085,6.552] | 0.857 [0.083,8.821] | 2.322 [0.219,24.582] | 1.586 [0.502,5.010] | 1.616 [0.512,5.099] |
| >=36months | 0.705 [0.08,6.157] | 0.783 [0.076,8.047] | 2.485 [0.235,26.182] | 1.607 [0.509,5.064] | 1.621 [0.515,5.105] |
| **Media exposure** |  |  |  |  |  |
| No | 1.000 | 1.000 | 1.000 | 1.000 | 1.000 |
| Partial | 0.832 [0.634,1.092] | 0.941 [0.708,1.251] | 0.687 [0.523,0.901] | 0.745 [0.612,0.909] | 0.75 [0.666,0.844] |
| Full | 0.627 [0.423,0.931] | 0.854 [0.596,1.223] | 0.532 [0.364,0.777] | 0.659 [0.415,1.047] | 0.653 [0.549,0.778] |
| **Wanted Pregnancy** |  |  |  |  |  |
| Wanted | 1.000 | 1.000 | 1.000 | 1.000 | 1.000 |
| Unwanted/Mistimed | 0.94 [0.755,1.169] | 1.322 [1.018,1.717] | 0.975 [0.736,1.292] | 1.296 [1.009,1.665] | 1.106 [0.98,1.248] |
| **ANC Visit during pregnancy** |  |  |  |  |  |
| <4 visit | 1.000 | 1.000 | 1.000 | 1.000 | 1.000 |
| >=4 visit | 0.824 [0.649,1.046] | 0.778 [0.625,0.968] | 0.69 [0.538,0.883] | 0.875 [0.679,1.126] | 0.806 [0.718,0.904] |
| **Place of delivery** |  |  |  |  |  |
| Health facility-Pub | 1.000 | 1.000 | 1.000 | 1.000 | 1.000 |
| Health facility-Pri | 1.425 [0.865,2.346] | 0.995 [0.717,1.382] | 1.028 [0.769,1.373] | 0.819 [0.614,1.094] | 0.951 [0.811,1.115] |
| Home/Other | 0.666 [0.494,0.898] | 1.154 [0.917,1.452] | 1.201 [0.87,1.658] | 1 [0.636,1.57] | 0.967 [0.841,1.111] |
| **Visited health facility in last 12 months** |  |  |  |  |  |
| No | 1.000 | 1.000 | 1.000 | 1.000 | 1.000 |
| Yes | 0.89 [0.731,1.084] | 0.801 [0.657,0.977] | 0.964 [0.786,1.183] | 0.864 [0.714,1.046] | 0.866 [0.788,0.951] |
| **Father's education** |  |  |  |  |  |
| no education | 1.000 | 1.000 | 1.000 | 1.000 | 1.000 |
| primary | 0.93 [0.71,1.219] | 0.803 [0.594,1.085] | 1.05 [0.747,1.476] | 0.919 [0.671,1.258] | 0.863 [0.746,0.998] |
| secondary | 1.012 [0.731,1.4] | 0.771 [0.551,1.079] | 0.899 [0.62,1.302] | 0.826 [0.594,1.148] | 0.807 [0.687,0.949] |
| higher | 0.789 [0.342,1.821] | 0.815 [0.447,1.485] | 0.715 [0.404,1.267] | 0.704 [0.42,1.18] | 0.658 [0.497,0.872] |
| **Father's occupation** |  |  |  |  |  |
| Not working | 1.000 | 1.000 | 1.000 | 1.000 | 1.000 |
| white collar | 0.71 [0.29,1.737] | 2.576 [0.829,8.006] | 1.641 [0.588,4.577] | 1.17 [0.712,1.923] | 1.068 [0.785,1.451] |
| Agri worker | 0.941 [0.399,2.219] | 2.629 [0.866,7.978] | 1.332 [0.467,3.797] | 1.407 [0.886,2.237] | 1.083 [0.808,1.451] |
| service/manual work | 0.922 [0.389,2.183] | 2.4 [0.791,7.279] | 1.496 [0.53,4.225] | 1.228 [0.788,1.911] | 1.067 [0.802,1.421] |
| **Place of residence** |  |  |  |  |  |
| Urban | 1.000 | 1.000 | 1.000 | 1.000 | 1.000 |
| Rural | 1.512 [1.142,2.001] | 0.706 [0.53,0.939] | 1.057 [0.79,1.414] | 1.11 [0.869,1.417] | 1.01 [0.886,1.151] |
| **Wealth Index** |  |  |  |  |  |
| Poorest | 1.000 | 1.000 | 1.000 | 1.000 | 1.000 |
| Poorer | 0.852 [0.651,1.115] | 1.22 [0.907,1.64] | 1.026 [0.76,1.384] | 1.16 [0.874,1.54] | 1.072 [0.934,1.231] |
| Middle | 0.829 [0.618,1.113] | 1.413 [1.032,1.934] | 1.241 [0.877,1.757] | 1.319 [0.975,1.785] | 1.167 [1.004,1.356] |
| Richer | 0.909 [0.644,1.282] | 1.254 [0.884,1.78] | 1.211 [0.836,1.754] | 1.106 [0.802,1.526] | 1.097 [0.929,1.294] |
| Richest | 0.941 [0.602,1.47] | 1.433 [0.924,2.222] | 1.256 [0.808,1.953] | 1.123 [0.741,1.702] | 1.235 [1.004,1.52] |
| **Household Size** |  |  |  |  |  |
| <=4 | 1.000 | 1.000 | 1.000 | 1.000 | 1.000 |
| 5-9 | 0.952 [0.756,1.198] | 1.11 [0.892,1.381] | 1.109 [0.889,1.382] | 1.031 [0.851,1.249] | 1.045 [0.941,1.159] |
| >=10 | 0.98 [0.663,1.449] | 1.123 [0.77,1.637] | 1.147 [0.76,1.733] | 0.937 [0.559,1.571] | 1.109 [0.908,1.354] |
| **Sex of head of household** |  |  |  |  |  |
| Male | 1.000 | 1.000 | 1.000 | 1.000 | 1.000 |
| Female | 1.044 [0.788,1.381] | 1.105 [0.874,1.396] | 0.756 [0.597,0.957] | 1.082 [0.879,1.331] | 0.998 [0.89,1.119] |
| **Year of Survey** |  |  |  |  |  |
| 2005 |  |  |  |  | 1.000 |
| 2010 |  |  |  |  | 0.797 [0.684,0.929] |
| 2014 |  |  |  |  | 0.784 [0.66,0.932] |
| 2021 |  |  |  |  | 0.919 [0.757,1.114] |

a= Cambodia DHS survey year 2005, b= Cambodia DHS survey year 2010, c= Cambodia DHS survey year 2014, d= Cambodia DHS survey year 2021, e= Cambodia DHS survey year 2005-2021

Source: Authors estimation using Demographic and Health Survey (DHS) Data.

**Appendix 2 TABLE 5** | Multinomial logistic regression results of inadequate consumption of Other Fruits and Vegetables and associated factors among children aged 6–23 months in Cambodia: based on 2005–2021 DHS.

|  | **CDHS 2004** | **CDHS 2010** | **CDHS 2014** | **CDHS 2021** | **Combined** |
| --- | --- | --- | --- | --- | --- |
| **Variable** | Odds Ratio^a^ [95% CI] | Odds Ratio^b^ [95% CI] | Odds Ratio^c^ [95% CI] | Odds Ratio^d^ [95% CI] | Odds Ratio^e^ [95% CI] |
| **Child Age** |  |  |  |  |  |
| 6-11months | 1.000 | 1.000 | 1.000 | 1.000 | 1.000 |
| 12-17months | 0.409 [0.312,0.537] | 0.453 [0.336,0.611] | 0.327 [0.246,0.434] | 0.45 [0.36,0.562] | 0.414 [0.364,0.472] |
| 18-23months | 0.343 [0.26,0.453] | 0.349 [0.258,0.473] | 0.211 [0.159,0.279] | 0.365 [0.289,0.459] | 0.321 [0.282,0.366] |
| **Child Sex** |  |  |  |  |  |
| Male | 1.000 | 1.000 | 1.000 | 1.000 | 1.000 |
| Female | 1.115 [0.905,1.374] | 0.984 [0.784,1.235] | 0.991 [0.804,1.221] | 1.03 [0.859,1.234] | 1.048 [0.948,1.159] |
| **Birth order** |  |  |  |  |  |
| First | 1.000 | 1.000 | 1.000 | 1.000 | 1.000 |
| Second or third | 1.4 [0.13,15.002] | 1.153 [0.065,20.29] | 0.897 [0.083,9.608] | 0.273 [0.017,4.269] | 0.998 [0.295,3.374] |
| Fourth or higher | 1.359 [0.124,14.797] | 1.061 [0.058,19.123] | 0.879 [0.079,9.695] | 0.337 [0.02,5.41] | 1.058 [0.309,3.622] |
| **Mother's age** |  |  |  |  |  |
| 15-19yrs | 1.000 | 1.000 | 1.000 | 1.000 | 1.000 |
| 20-34yrs | 1.238 [0.738,2.078] | 1.007 [0.574,1.765] | 0.818 [0.495,1.35] | 1.083 [0.702,1.67] | 1.055 [0.827,1.346] |
| 35-49yrs | 1.537 [0.844,2.799] | 1.108 [0.563,2.181] | 0.759 [0.407,1.417] | 1.055 [0.638,1.746] | 1.062 [0.797,1.415] |
| **Mother's Education** |  |  |  |  |  |
| No education | 1.000 | 1.000 | 1.000 | 1.000 | 1.000 |
| Primary | 1.11 [0.837,1.472] | 0.84 [0.577,1.223] | 0.823 [0.558,1.212] | 1.058 [0.77,1.454] | 0.965 [0.821,1.133] |
| Secondary | 1.291 [0.858,1.941] | 0.585 [0.375,0.913] | 0.703 [0.454,1.09] | 0.816 [0.574,1.16] | 0.789 [0.652,0.955] |
| Higher | 1.847 [0.353,9.649] | 0.895 [0.346,2.319] | 0.57 [0.285,1.14] | 0.832 [0.464,1.491] | 0.758 [0.53,1.085] |
| **Mother's Occupation** |  |  |  |  |  |
| Not working | 1.000 | 1.000 | 1.000 | 1.000 | 1.000 |
| White collar | 0.54 [0.111,2.636] | 0.908 [0.645,1.279] | 1.048 [0.764,1.438] | 0.634 [0.487,0.826] | 0.847 [0.721,0.995] |
| Agri worker | 0.429 [0.089,2.073] | 1.398 [0.979,1.997] | 1.172 [0.86,1.597] | 0.797 [0.593,1.07] | 0.935 [0.794,1.101] |
| Service/manual work | 0.568 [0.117,2.742] | 1.027 [0.686,1.537] | 1.183 [0.843,1.661] | 0.965 [0.737,1.263] | 1.06 [0.898,1.251] |
| **Marital Status** |  |  |  |  |  |
| Married | 1.000 | 1.000 | 1.000 | 1.000 | 1.000 |
| Not married | 0.699 [0.399,1.223] | 0.814 [0.449,1.473] | 0.78 [0.466,1.303] | 0.903 [0.452,1.803] | 0.78 [0.591,1.029] |
| **Preceding birth interval** |  |  |  |  |  |
| first birth | 1.000 | 1.000 | 1.000 | 1.000 | 1.000 |
| <36months | 0.697 [0.064,7.485] | 0.771 [0.043,13.675] | 1.585 [0.145,17.237] | 2.975 [0.189,46.787] | 1.023 [0.301,3.47] |
| >=36months | 0.544 [0.05,5.828] | 0.764 [0.043,13.473] | 1.155 [0.107,12.462] | 2.906 [0.186,45.206] | 0.872 [0.258,2.95] |
| **Media exposure** |  |  |  |  |  |
| No | 1.000 | 1.000 | 1.000 | 1.000 | 1.000 |
| Partial | 0.66 [0.471,0.926] | 0.789 [0.528,1.177] | 0.807 [0.577,1.128] | 0.718 [0.585,0.88] | 0.711 [0.62,0.816] |
| Full | 0.472 [0.302,0.739] | 0.836 [0.521,1.34] | 0.613 [0.4,0.938] | 0.873 [0.547,1.392] | 0.635 [0.523,0.772] |
| **Wanted Pregnancy** |  |  |  |  |  |
| Wanted | 1.000 | 1.000 | 1.000 | 1.000 | 1.000 |
| Unwanted/Mistimed | 1.112 [0.867,1.426] | 1.164 [0.826,1.641] | 1.091 [0.797,1.494] | 1.031 [0.796,1.334] | 1.07 [0.932,1.227] |
| **ANC Visit during pregnancy** |  |  |  |  |  |
| <4 visit | 1.000 | 1.000 | 1.000 | 1.000 | 1.000 |
| >=4 visit | 0.825 [0.637,1.067] | 1.036 [0.791,1.356] | 0.891 [0.67,1.186] | 0.885 [0.682,1.15] | 0.9 [0.79,1.027] |
| **Place of delivery** |  |  |  |  |  |
| Health facility-Pub | 1.000 | 1.000 | 1.000 | 1.000 | 1.000 |
| Health facility-Pri | 1.097 [0.64,1.878] | 0.576 [0.405,0.819] | 0.923 [0.684,1.245] | 0.703 [0.524,0.943] | 0.748 [0.633,0.884] |
| Home/Other | 1.008 [0.73,1.393] | 1.307 [0.978,1.747] | 1.329 [0.899,1.964] | 0.789 [0.494,1.26] | 1.113 [0.947,1.309] |
| **Visited health facility in last 12 months** |  |  |  |  |  |
| No | 1.000 | 1.000 | 1.000 | 1.000 | 1.000 |
| Yes | 1.045 [0.837,1.303] | 1.125 [0.884,1.432] | 0.861 [0.684,1.082] | 0.643 [0.528,0.783] | 0.843 [0.758,0.936] |
| **Father's education** |  |  |  |  |  |
| no education | 1.000 | 1.000 | 1.000 | 1.000 | 1.000 |
| primary | 0.715 [0.516,0.99] | 0.69 [0.448,1.064] | 0.991 [0.658,1.49] | 0.767 [0.553,1.064] | 0.759 [0.638,0.904] |
| secondary | 0.662 [0.453,0.968] | 0.753 [0.476,1.191] | 0.98 [0.636,1.511] | 0.96 [0.682,1.351] | 0.806 [0.668,0.973] |
| higher | 0.972 [0.398,2.37] | 0.638 [0.31,1.313] | 0.96 [0.522,1.764] | 0.929 [0.55,1.57] | 0.817 [0.605,1.104] |
| **Father's occupation** |  |  |  |  |  |
| Not working | 1.000 | 1.000 | 1.000 | 1.000 | 1.000 |
| white collar | 0.293 [0.088,0.977] | 3.543 [1.078,11.634] | 1.649 [0.633,4.295] | 0.931 [0.558,1.555] | 0.836 [0.602,1.161] |
| Agri worker | 0.41 [0.126,1.331] | 3.635 [1.136,11.622] | 2.04 [0.762,5.457] | 0.836 [0.516,1.354] | 0.926 [0.675,1.27] |
| service/manual work | 0.49 [0.151,1.594] | 2.541 [0.798,8.094] | 1.938 [0.734,5.113] | 0.868 [0.548,1.375] | 0.864 [0.635,1.174] |
| **Place of residence** |  |  |  |  |  |
| Urban | 1.000 | 1.000 | 1.000 | 1.000 | 1.000 |
| Rural | 1.435 [1.066,1.931] | 0.805 [0.576,1.126] | 0.719 [0.523,0.986] | 1.195 [0.93,1.535] | 1.002 [0.868,1.156] |
| **Wealth Index** |  |  |  |  |  |
| Poorest | 1.000 | 1.000 | 1.000 | 1.000 | 1.000 |
| Poorer | 1.15 [0.837,1.58] | 0.71 [0.481,1.047] | 1.136 [0.793,1.628] | 0.97 [0.724,1.299] | 1.032 [0.878,1.213] |
| Middle | 0.934 [0.668,1.306] | 1.03 [0.677,1.567] | 0.909 [0.611,1.351] | 1.1 [0.805,1.502] | 1.07 [0.899,1.273] |
| Richer | 0.724 [0.495,1.058] | 0.823 [0.533,1.272] | 0.805 [0.53,1.223] | 1.001 [0.72,1.393] | 0.904 [0.75,1.088] |
| Richest | 0.761 [0.466,1.242] | 0.699 [0.413,1.183] | 0.654 [0.399,1.07] | 0.722 [0.472,1.104] | 0.784 [0.624,0.986] |
| **Household Size** |  |  |  |  |  |
| <=4 | 1.000 | 1.000 | 1.000 | 1.000 | 1.000 |
| 5-9 | 1.224 [0.948,1.581] | 0.914 [0.699,1.194] | 1.173 [0.919,1.496] | 0.909 [0.746,1.106] | 1 [0.891,1.123] |
| >=10 | 1.215 [0.783,1.886] | 0.903 [0.55,1.483] | 1.42 [0.893,2.259] | 0.743 [0.435,1.268] | 1.079 [0.857,1.36] |
| **Sex of head of household** |  |  |  |  |  |
| Male | 1.000 | 1.000 | 1.000 | 1.000 | 1.000 |
| Female | 1.017 [0.74,1.396] | 1.517 [1.121,2.053] | 0.913 [0.709,1.175] | 1.051 [0.849,1.301] | 1.096 [0.965,1.245] |
| **Year of Survey** |  |  |  |  |  |
| 2005 |  |  |  |  | 1.000 |
| 2010 |  |  |  |  | 1.656 [1.381,1.986] |
| 2014 |  |  |  |  | 1.012 [0.832,1.23] |
| 2021 |  |  |  |  | 0.352 [0.283,0.438] |

a= Cambodia DHS survey year 2005, b= Cambodia DHS survey year 2010, c= Cambodia DHS survey year 2014, d= Cambodia DHS survey year 2021, e= Cambodia DHS survey year 2005-2021

Source: Authors estimation using Demographic and Health Survey (DHS) Data.

**Appendix 2 TABLE 6** | Multinomial logistic regression results of inadequate consumption of Eggs and associated factors among children aged 6–23 months in Cambodia: based on 2005–2021 DHS.

|  | **CDHS 2004** | **CDHS 2010** | **CDHS 2014** | **CDHS 2021** | **Combined** |
| --- | --- | --- | --- | --- | --- |
| **Variables** | Odds Ratio^a^ [95% CI] | Odds Ratio^b^ [95% CI] | Odds Ratio^c^ [95% CI] | Odds Ratio^d^ [95% CI] | Odds Ratio^e^ [95% CI] |
| **Child Age** |  |  |  |  |  |
| 6-11months | 1.000 | 1.000 | 1.000 | 1.000 | 1.000 |
| 12-17months | 0.565 [0.42,0.761] | 0.612 [0.487,0.77] | 0.5 [0.394,0.634] | 0.635 [0.509,0.792] | 0.588 [0.522,0.661] |
| 18-23months | 0.536 [0.394,0.728] | 0.52 [0.41,0.66] | 0.432 [0.342,0.547] | 0.542 [0.432,0.679] | 0.522 [0.463,0.588] |
| **Child Sex** |  |  |  |  |  |
| Male | 1.000 | 1.000 | 1.000 | 1.000 | 1.000 |
| Female | 1.141 [0.902,1.444] | 0.987 [0.821,1.187] | 0.982 [0.814,1.185] | 1.006 [0.841,1.204] | 1.026 [0.933,1.128] |
| **Birth order** |  |  |  |  |  |
| First | 1.000 | 1.000 | 1.000 | 1.000 | 1.000 |
| Second or third | 0.781 [0.067,9.059] | 0.518 [0.061,4.365] | 0.788 [0.103,6.036] | 0.256 [0.019,3.363] | 0.587 [0.197,1.749] |
| Fourth or higher | 1.06 [0.089,12.556] | 0.602 [0.07,5.183] | 0.738 [0.094,5.807] | 0.216 [0.016,2.925] | 0.643 [0.213,1.938] |
| **Mother's age** |  |  |  |  |  |
| 15-19yrs | 1.000 | 1.000 | 1.000 | 1.000 | 1.000 |
| 20-34yrs | 0.958 [0.518,1.77] | 1.026 [0.662,1.592] | 1.13 [0.741,1.724] | 1.053 [0.685,1.618] | 1.034 [0.825,1.295] |
| 35-49yrs | 0.8 [0.397,1.613] | 1.05 [0.615,1.792] | 1.037 [0.606,1.775] | 0.978 [0.594,1.611] | 0.943 [0.721,1.233] |
| **Mother's Education** |  |  |  |  |  |
| No education | 1.000 | 1.000 | 1.000 | 1.000 | 1.000 |
| Primary | 0.961 [0.695,1.33] | 0.759 [0.568,1.013] | 0.899 [0.645,1.252] | 0.709 [0.512,0.983] | 0.832 [0.714,0.971] |
| Secondary | 0.797 [0.509,1.247] | 0.627 [0.439,0.897] | 0.802 [0.548,1.172] | 0.702 [0.49,1.005] | 0.753 [0.628,0.903] |
| Higher | 0.265 [0.058,1.205] | 0.667 [0.308,1.446] | 1.104 [0.584,2.086] | 0.707 [0.398,1.253] | 0.794 [0.565,1.115] |
| **Mother's Occupation** |  |  |  |  |  |
| Not working | 1.000 | 1.000 | 1.000 | 1.000 | 1.000 |
| White collar | 0.286 [0.034,2.407] | 0.869 [0.648,1.164] | 0.945 [0.705,1.266] | 0.795 [0.614,1.03] | 0.877 [0.755,1.019] |
| Agri worker | 0.25 [0.03,2.092] | 1.047 [0.784,1.397] | 0.993 [0.755,1.306] | 1.381 [1.024,1.863] | 1.051 [0.904,1.22] |
| Service/manual work | 0.261 [0.031,2.177] | 0.729 [0.518,1.026] | 1.164 [0.852,1.59] | 1.078 [0.827,1.406] | 0.995 [0.851,1.162] |
| **Marital Status** |  |  |  |  |  |
| Married | 1.000 | 1.000 | 1.000 | 1.000 | 1.000 |
| Not married | 0.865 [0.465,1.608] | 0.715 [0.438,1.164] | 1.199 [0.738,1.948] | 1.029 [0.515,2.057] | 0.941 [0.722,1.227] |
| **Preceding birth interval** |  |  |  |  |  |
| first birth | 1.000 | 1.000 | 1.000 | 1.000 | 1.000 |
| <36months | 1.046 [0.09,12.163] | 2.044 [0.241,17.309] | 1.54 [0.198,11.935] | 3.559 [0.269,47.018] | 1.745 [0.584,5.213] |
| >=36months | 1.115 [0.096,12.912] | 1.756 [0.208,14.827] | 1.435 [0.186,11.048] | 4.142 [0.316,54.131] | 1.698 [0.57,5.06] |
| **Media exposure** |  |  |  |  |  |
| No | 1.000 | 1.000 | 1.000 | 1.000 | 1.000 |
| Partial | 0.537 [0.358,0.805] | 1.325 [0.988,1.776] | 0.988 [0.744,1.31] | 0.738 [0.604,0.902] | 0.834 [0.734,0.948] |
| Full | 0.463 [0.276,0.776] | 1.48 [1.031,2.125] | 0.655 [0.45,0.953] | 0.622 [0.393,0.982] | 0.74 [0.618,0.887] |
| **Wanted Pregnancy** |  |  |  |  |  |
| Wanted | 1.000 | 1.000 | 1.000 | 1.000 | 1.000 |
| Unwanted/Mistimed | 1.113 [0.839,1.478] | 0.984 [0.75,1.291] | 1.277 [0.961,1.697] | 1.278 [0.989,1.652] | 1.162 [1.018,1.327] |
| **ANC Visit during pregnancy** |  |  |  |  |  |
| <4 visit | 1.000 | 1.000 | 1.000 | 1.000 | 1.000 |
| >=4 visit | 0.89 [0.669,1.184] | 0.808 [0.649,1.006] | 0.848 [0.658,1.093] | 0.915 [0.704,1.191] | 0.874 [0.773,0.988] |
| **Place of delivery** |  |  |  |  |  |
| Health facility-Pub | 1.000 | 1.000 | 1.000 | 1.000 | 1.000 |
| Health facility-Pri | 0.911 [0.511,1.624] | 0.972 [0.707,1.336] | 1.11 [0.837,1.471] | 0.886 [0.665,1.18] | 0.981 [0.837,1.151] |
| Home/Other | 0.93 [0.651,1.329] | 1.056 [0.838,1.329] | 1.101 [0.789,1.537] | 1.454 [0.887,2.381] | 1.034 [0.891,1.2] |
| **Visited health facility in last 12 months** |  |  |  |  |  |
| No | 1.000 | 1.000 | 1.000 | 1.000 | 1.000 |
| Yes | 1.067 [0.831,1.368] | 1.031 [0.846,1.256] | 0.913 [0.742,1.122] | 1.026 [0.844,1.246] | 0.992 [0.898,1.096] |
| **Father's education** |  |  |  |  |  |
| no education | 1.000 | 1.000 | 1.000 | 1.000 | 1.000 |
| primary | 1.038 [0.721,1.494] | 0.676 [0.487,0.937] | 1.134 [0.798,1.61] | 0.886 [0.636,1.232] | 0.894 [0.758,1.053] |
| secondary | 0.861 [0.568,1.304] | 0.608 [0.427,0.868] | 0.858 [0.59,1.248] | 0.885 [0.628,1.248] | 0.765 [0.641,0.913] |
| higher | 1.492 [0.549,4.058] | 0.571 [0.313,1.04] | 0.833 [0.478,1.449] | 0.732 [0.438,1.225] | 0.717 [0.54,0.953] |
| **Father's occupation** |  |  |  |  |  |
| Not working | 1.000 | 1.000 | 1.000 | 1.000 | 1.000 |
| white collar | 0.752 [0.228,2.477] | 2.099 [0.739,5.962] | 0.775 [0.305,1.968] | 0.729 [0.441,1.206] | 0.845 [0.615,1.161] |
| Agri worker | 0.975 [0.305,3.117] | 1.89 [0.682,5.239] | 0.823 [0.316,2.141] | 0.822 [0.51,1.323] | 0.9 [0.663,1.223] |
| service/manual work | 0.724 [0.227,2.308] | 1.708 [0.616,4.732] | 0.815 [0.316,2.096] | 0.78 [0.495,1.228] | 0.826 [0.613,1.113] |
| **Place of residence** |  |  |  |  |  |
| Urban | 1.000 | 1.000 | 1.000 | 1.000 | 1.000 |
| Rural | 1.156 [0.826,1.618] | 0.85 [0.639,1.131] | 0.98 [0.735,1.307] | 0.986 [0.769,1.264] | 0.963 [0.841,1.103] |
| **Wealth Index** |  |  |  |  |  |
| Poorest | 1.000 | 1.000 | 1.000 | 1.000 | 1.000 |
| Poorer | 0.87 [0.601,1.259] | 0.87 [0.646,1.173] | 0.943 [0.696,1.278] | 0.757 [0.565,1.014] | 0.895 [0.77,1.04] |
| Middle | 0.684 [0.465,1.006] | 1.001 [0.73,1.374] | 1.041 [0.731,1.481] | 0.851 [0.624,1.161] | 0.953 [0.81,1.121] |
| Richer | 0.665 [0.431,1.024] | 0.981 [0.694,1.388] | 1.043 [0.716,1.519] | 0.927 [0.669,1.286] | 0.953 [0.799,1.136] |
| Richest | 0.831 [0.482,1.435] | 0.967 [0.627,1.491] | 0.774 [0.497,1.203] | 0.986 [0.649,1.498] | 0.927 [0.746,1.151] |
| **Household Size** |  |  |  |  |  |
| <=4 | 1.000 | 1.000 | 1.000 | 1.000 | 1.000 |
| 5-9 | 1.14 [0.858,1.515] | 0.806 [0.648,1.003] | 1.029 [0.825,1.282] | 1.097 [0.903,1.334] | 1 [0.897,1.115] |
| >=10 | 0.941 [0.578,1.529] | 0.901 [0.607,1.337] | 0.971 [0.644,1.464] | 1.061 [0.63,1.786] | 1 [0.806,1.241] |
| **Sex of head of household** |  |  |  |  |  |
| Male | 1.000 | 1.000 | 1.000 | 1.000 | 1.000 |
| Female | 1.023 [0.722,1.45] | 1.028 [0.815,1.297] | 0.998 [0.793,1.257] | 0.984 [0.797,1.214] | 1.012 [0.899,1.14] |
| **Year of Survey** |  |  |  |  |  |
| 2005 |  |  |  |  | 1.000 |
| 2010 |  |  |  |  | 0.442 [0.372,0.525] |
| 2014 |  |  |  |  | 0.369 [0.306,0.447] |
| 2021 |  |  |  |  | 0.296 [0.239,0.366] |

a= Cambodia DHS survey year 2005, b= Cambodia DHS survey year 2010, c= Cambodia DHS survey year 2014, d= Cambodia DHS survey year 2021, e= Cambodia DHS survey year 2005-2021

Source: Authors estimation using Demographic and Health Survey (DHS) Data.

**Appendix 2 TABLE 7** | Multinomial logistic regression results of inadequate consumption of Flesh Foods and associated factors among children aged 6–23 months in Cambodia: based on 2005–2021 DHS.

|  | **CDHS 2004** | **CDHS 2010** | **CDHS 2014** | **CDHS 2021** | **Combined** |
| --- | --- | --- | --- | --- | --- |
| **Variables** | Odds Ratio^a^ [95% CI] | Odds Ratio^b^ [95% CI] | Odds Ratio^c^ [95% CI] | Odds Ratio^d^ [95% CI] | Odds Ratio^e^ [95% CI] |
| **Child Age** |  |  |  |  |  |
| 6-11months | 1.000 | 1.000 | 1.000 | 1.000 | 1.000 |
| 12-17months | 0.177 [0.136,0.23] | 0.183 [0.139,0.241] | 0.214 [0.16,0.286] | 0.226 [0.173,0.296] | 0.207 [0.182,0.237] |
| 18-23months | 0.104 [0.076,0.142] | 0.132 [0.098,0.179] | 0.126 [0.091,0.174] | 0.137 [0.101,0.185] | 0.134 [0.115,0.155] |
| **Child Sex** |  |  |  |  |  |
| Male | 1.000 | 1.000 | 1.000 | 1.000 | 1.000 |
| Female | 1.113 [0.895,1.385] | 1.08 [0.868,1.344] | 0.946 [0.749,1.195] | 0.983 [0.789,1.224] | 1.037 [0.93,1.157] |
| **Birth order** |  |  |  |  |  |
| First | 1.000 | 1.000 | 1.000 | 1.000 | 1.000 |
| Second or third | 1.002 [0.083,12.017] | 0.924 [0.062,13.611] | 0.886 [0.113,6.916] | 1.332 [0.067,26.451] | 0.542 [0.135,2.174] |
| Fourth or higher | 0.868 [0.071,10.565] | 1.013 [0.067,15.223] | 0.78 [0.083,7.319] | 1.45 [0.07,29.881] | 0.546 [0.134,2.214] |
| **Mother's age** |  |  |  |  |  |
| 15-19yrs | 1.000 | 1.000 | 1.000 | 1.000 | 1.000 |
| 20-34yrs | 1.451 [0.832,2.528] | 0.666 [0.416,1.068] | 0.8 [0.489,1.307] | 0.961 [0.595,1.553] | 0.974 [0.762,1.244] |
| 35-49yrs | 2.069 [1.094,3.912] | 0.687 [0.381,1.238] | 0.735 [0.387,1.396] | 0.969 [0.545,1.724] | 1.033 [0.769,1.389] |
| **Mother's Education** |  |  |  |  |  |
| No education | 1.000 | 1.000 | 1.000 | 1.000 | 1.000 |
| Primary | 1 [0.754,1.326] | 0.72 [0.527,0.983] | 0.798 [0.547,1.165] | 0.803 [0.551,1.17] | 0.818 [0.697,0.96] |
| Secondary | 0.827 [0.532,1.284] | 0.528 [0.349,0.799] | 0.748 [0.48,1.163] | 0.788 [0.516,1.203] | 0.748 [0.612,0.915] |
| Higher | 3.35 [0.658,17.055] | 0.37 [0.135,1.008] | 0.681 [0.295,1.574] | 1.192 [0.577,2.46] | 0.831 [0.54,1.277] |
| **Mother's Occupation** |  |  |  |  |  |
| Not working | 1.000 | 1.000 | 1.000 | 1.000 | 1.000 |
| White collar | 1.147 [0.282,4.659] | 0.87 [0.609,1.242] | 0.7 [0.484,1.011] | 0.669 [0.483,0.925] | 0.702 [0.584,0.843] |
| Agri worker | 1.262 [0.317,5.013] | 1.013 [0.725,1.416] | 0.546 [0.396,0.753] | 0.786 [0.552,1.119] | 0.694 [0.585,0.823] |
| Service/manual work | 1.623 [0.408,6.457] | 0.846 [0.553,1.292] | 1.017 [0.697,1.483] | 0.856 [0.622,1.18] | 0.873 [0.729,1.047] |
| **Marital Status** |  |  |  |  |  |
| Married | 1.000 | 1.000 | 1.000 | 1.000 | 1.000 |
| Not married | 1.036 [0.567,1.893] | 0.99 [0.543,1.803] | 0.875 [0.467,1.64] | 1.672 [0.723,3.866] | 1.041 [0.765,1.416] |
| **Preceding birth interval** |  |  |  |  |  |
| first birth | 1.000 | 1.000 | 1.000 | 1.000 | 1.000 |
| <36months | 0.92 [0.076,11.067] | 0.857 [0.057,12.684] | 1.124 [0.692,1.824] | 0.687 [0.034,13.801] | 1.689 [0.42,6.792] |
| >=36months | 0.812 [0.067,9.759] | 1.054 [0.071,15.573] | 1.209 [0.524,2.785] | 0.599 [0.03,11.841] | 1.672 [0.416,6.706] |
| **Media exposure** |  |  |  |  |  |
| No | 1.000 | 1.000 | 1.000 | 1.000 | 1.000 |
| Partial | 0.581 [0.431,0.785] | 0.602 [0.437,0.829] | 0.895 [0.647,1.239] | 0.952 [0.741,1.224] | 0.743 [0.645,0.856] |
| Full | 0.559 [0.351,0.892] | 0.598 [0.393,0.908] | 0.66 [0.411,1.062] | 1.946 [1.136,3.333] | 0.723 [0.583,0.896] |
| **Wanted Pregnancy** |  |  |  |  |  |
| Wanted | 1.000 | 1.000 | 1.000 | 1.000 | 1.000 |
| Unwanted/Mistimed | 0.958 [0.74,1.239] | 1.067 [0.787,1.447] | 0.834 [0.584,1.19] | 0.932 [0.682,1.272] | 0.971 [0.839,1.125] |
| **ANC Visit during pregnancy** |  |  |  |  |  |
| <4 visit | 1.000 | 1.000 | 1.000 | 1.000 | 1.000 |
| >=4 visit | 0.848 [0.634,1.134] | 0.791 [0.609,1.026] | 0.966 [0.711,1.312] | 0.546 [0.403,0.739] | 0.773 [0.672,0.889] |
| **Place of delivery** |  |  |  |  |  |
| Health facility-Pub | 1.000 | 1.000 | 1.000 | 1.000 | 1.000 |
| Health facility-Pri | 0.625 [0.327,1.194] | 0.794 [0.52,1.212] | 0.911 [0.632,1.312] | 0.689 [0.478,0.993] | 0.775 [0.632,0.951] |
| Home/Other | 0.774 [0.542,1.105] | 0.956 [0.724,1.262] | 0.948 [0.637,1.412] | 0.782 [0.444,1.376] | 0.923 [0.78,1.093] |
| **Visited health facility in last 12 months** |  |  |  |  |  |
| No | 1.000 | 1.000 | 1.000 | 1.000 | 1.000 |
| Yes | 0.996 [0.79,1.255] | 0.854 [0.674,1.083] | 1.091 [0.846,1.406] | 0.653 [0.515,0.827] | 0.822 [0.733,0.922] |
| **Father's education** |  |  |  |  |  |
| no education | 1.000 | 1.000 | 1.000 | 1.000 | 1.000 |
| primary | 0.792 [0.586,1.073] | 0.894 [0.635,1.259] | 0.744 [0.498,1.111] | 1.013 [0.69,1.488] | 0.818 [0.69,0.969] |
| secondary | 0.728 [0.501,1.06] | 0.694 [0.469,1.026] | 0.773 [0.498,1.201] | 1.007 [0.671,1.51] | 0.76 [0.628,0.919] |
| higher | 0.919 [0.339,2.491] | 0.868 [0.421,1.789] | 0.594 [0.287,1.226] | 0.772 [0.398,1.5] | 0.653 [0.458,0.931] |
| **Father's occupation** |  |  |  |  |  |
| Not working | 1.000 | 1.000 | 1.000 | 1.000 | 1.000 |
| white collar | 0.813 [0.289,2.285] | 1.497 [0.448,5.003] | 1.362 [0.915,2.027] | 1.392 [0.739,2.623] | 1.384 [0.942,2.034] |
| Agri worker | 1.072 [0.402,2.859] | 1.423 [0.44,4.593] | 0.951 [0.704,1.285] | 1.6 [0.89,2.877] | 1.333 [0.926,1.918] |
| service/manual work | 0.811 [0.3,2.19] | 1.597 [0.495,5.148] | 1 [0,0] | 1.352 [0.768,2.38] | 1.255 [0.878,1.794] |
| **Place of residence** |  |  |  |  |  |
| Urban | 1.000 | 1.000 | 1.000 | 1.000 | 1.000 |
| Rural | 1.484 [1.064,2.069] | 0.972 [0.679,1.391] | 1.328 [0.927,1.901] | 1.241 [0.916,1.682] | 1.18 [1.003,1.389] |
| **Wealth Index** |  |  |  |  |  |
| Poorest | 1.000 | 1.000 | 1.000 | 1.000 | 1.000 |
| Poorer | 0.957 [0.703,1.302] | 1.459 [1.038,2.05] | 0.805 [0.558,1.161] | 0.878 [0.616,1.252] | 1.036 [0.878,1.222] |
| Middle | 0.647 [0.456,0.917] | 1.185 [0.815,1.721] | 0.85 [0.558,1.293] | 1.038 [0.718,1.499] | 0.959 [0.8,1.15] |
| Richer | 0.825 [0.546,1.247] | 1.278 [0.842,1.941] | 0.802 [0.512,1.256] | 0.957 [0.642,1.426] | 0.982 [0.802,1.201] |
| Richest | 1.252 [0.739,2.121] | 1.73 [1.022,2.928] | 1.051 [0.621,1.779] | 0.917 [0.54,1.559] | 1.251 [0.971,1.611] |
| **Household Size** |  |  |  |  |  |
| <=4 | 1.000 | 1.000 | 1.000 | 1.000 | 1.000 |
| 5-9 | 1.165 [0.883,1.538] | 1.027 [0.788,1.337] | 1.119 [0.852,1.469] | 0.965 [0.76,1.227] | 1.063 [0.935,1.208] |
| >=10 | 1.185 [0.749,1.875] | 1.027 [0.66,1.599] | 0.85 [0.499,1.448] | 0.953 [0.501,1.813] | 1.038 [0.816,1.32] |
| **Sex of head of household** |  |  |  |  |  |
| Male | 1.000 | 1.000 | 1.000 | 1.000 | 1.000 |
| Female | 1.018 [0.73,1.419] | 1.151 [0.871,1.52] | 0.879 [0.655,1.181] | 1.116 [0.865,1.441] | 1.06 [0.921,1.219] |
| **Year of Survey** |  |  |  |  |  |
| 2005 |  |  |  |  | 1.000 |
| 2010 |  |  |  |  | 1.072 [0.89,1.292] |
| 2014 |  |  |  |  | 0.819 [0.661,1.014] |
| 2021 |  |  |  |  | 0.875 [0.691,1.108] |

a= Cambodia DHS survey year 2005, b= Cambodia DHS survey year 2010, c= Cambodia DHS survey year 2014, d= Cambodia DHS survey year 2021, e= Cambodia DHS survey year 2005-2021

Source: Authors estimation using Demographic and Health Survey (DHS) Data.

**Appendix 2 TABLE 8** | Multinomial logistic regression results of inadequate consumption of Legumes and nuts and associated factors among children aged 6–23 months in Cambodia: based on 2005–2021 DHS.

|  | **CDHS 2004** | **CDHS 2010** | **CDHS 2014** | **CDHS 2021** | **Combined** |
| --- | --- | --- | --- | --- | --- |
| **Variables** | Odds Ratio^a^ [95% CI] | Odds Ratio^b^ [95% CI] | Odds Ratio^c^ [95% CI] | Odds Ratio^d^ [95% CI] | Odds Ratio^e^ [95% CI] |
| **Child Age** |  |  |  |  |  |
| 6-11months | 1.000 | 1.000 | 1.000 | 1.000 | 1.000 |
| 12-17months | 0.363 [0.219,0.602] | 0.566 [0.355,0.902] | 0.372 [0.231,0.597] | 0.142 [0.104,0.195] | 0.45 [0.347,0.583] |
| 18-23months | 0.327 [0.193,0.555] | 0.477 [0.299,0.762] | 0.314 [0.198,0.498] | 0.079 [0.053,0.118] | 0.384 [0.296,0.497] |
| **Child Sex** |  |  |  |  |  |
| Male | 1.000 | 1.000 | 1.000 | 1.000 | 1.000 |
| Female | 1.335 [0.918,1.94] | 1.128 [0.795,1.601] | 1.042 [0.749,1.451] | 1.043 [0.811,1.341] | 1.231 [1.019,1.488] |
| **Birth order** |  |  |  |  |  |
| First | 1.000 | 1.000 | 1.000 | 1.000 | 1.000 |
| Second or third | 0.219 [0.016,2.989] | 0.365 [0.024,5.566] | 1.124 [0.692,1.825] | 1.954 [0.187,20.322] | 0.365 [0.07,1.883] |
| Fourth or higher | 0.186 [0.013,2.658] | 0.269 [0.016,4.346] | 1.208 [0.524,2.785] | 1.348 [0.126,14.343] | 0.366 [0.068,1.949] |
| **Mother's age** |  |  |  |  |  |
| 15-19yrs | 1.000 | 1.000 | 1.000 | 1.000 | 1.000 |
| 20-34yrs | 1.368 [0.542,3.45] | 0.751 [0.279,2.023] | 0.759 [0.31,1.857] | 1.063 [0.577,1.957] | 0.877 [0.527,1.46] |
| 35-49yrs | 2.051 [0.696,6.045] | 0.954 [0.302,3.013] | 0.941 [0.314,2.816] | 1.406 [0.69,2.864] | 1.124 [0.619,2.041] |
| **Mother's Education** |  |  |  |  |  |
| No education | 1.000 | 1.000 | 1.000 | 1.000 | 1.000 |
| Primary | 1.151 [0.685,1.934] | 0.711 [0.395,1.276] | 1.969 [1.127,3.438] | 0.743 [0.534,1.035] | 1.053 [0.776,1.428] |
| Secondary | 1.18 [0.583,2.388] | 0.513 [0.256,1.028] | 2.201 [1.157,4.187] | 0.958 [0.594,1.545] | 0.97 [0.674,1.397] |
| Higher | 1.042 [0.085,12.647] | 0.44 [0.107,1.797] | 1.036 [0.395,2.716] | 2.341 [0.443,12.365] | 0.589 [0.311,1.115] |
| **Mother's Occupation** |  |  |  |  |  |
| Not working | 1.000 | 1.000 | 1.000 | 1.000 | 1.000 |
| White collar | 1.038 [0.615,1.751] | 0.765 [0.445,1.316] | 1.278 [0.769,2.122] | 1.011 [0.224,4.553] | 0.934 [0.68,1.283] |
| Agri worker | 1.15 [0.706,1.875] | 0.983 [0.562,1.719] | 1.208 [0.737,1.979] | 0.888 [0.201,3.919] | 0.977 [0.707,1.35] |
| Service/manual work | 1 [0,0] | 0.755 [0.397,1.437] | 0.896 [0.544,1.476] | 1.344 [0.304,5.928] | 0.755 [0.548,1.04] |
| **Marital Status** |  |  |  |  |  |
| Married | 1.000 | 1.000 | 1.000 | 1.000 | 1.000 |
| Not married | 1.195 [0.411,3.47] | 0.952 [0.371,2.442] | 0.74 [0.365,1.498] | 0.865 [0.417,1.791] | 0.848 [0.531,1.356] |
| **Preceding birth interval** |  |  |  |  |  |
| first birth | 1.000 | 1.000 | 1.000 | 1.000 | 1.000 |
| <36months | 4.644 [0.338,63.733] | 2.454 [0.159,37.838] | 0.84 [0.539,1.309] | 0.467 [0.044,4.886] | 2.88 [0.553,15.003] |
| >=36months | 3.177 [0.234,42.977] | 2.381 [0.155,36.454] | 0.797 [0.477,1.332] | 0.488 [0.046,5.078] | 2.228 [0.43,11.528] |
| **Media exposure** |  |  |  |  |  |
| No | 1.000 | 1.000 | 1.000 | 1.000 | 1.000 |
| Partial | 0.663 [0.326,1.35] | 0.792 [0.428,1.467] | 0.767 [0.439,1.338] | 0.905 [0.628,1.304] | 0.74 [0.543,1.009] |
| Full | 1.277 [0.523,3.114] | 1.132 [0.543,2.359] | 0.635 [0.321,1.258] | 0.756 [0.441,1.294] | 0.765 [0.52,1.126] |
| **Wanted Pregnancy** |  |  |  |  |  |
| Wanted | 1.000 | 1.000 | 1.000 | 1.000 | 1.000 |
| Unwanted/Mistimed | 0.804 [0.524,1.233] | 0.852 [0.522,1.39] | 0.726 [0.455,1.159] | 1.173 [0.875,1.571] | 0.763 [0.597,0.974] |
| **ANC Visit during pregnancy** |  |  |  |  |  |
| <4 visit | 1.000 | 1.000 | 1.000 | 1.000 | 1.000 |
| >=4 visit | 0.862 [0.557,1.334] | 0.8 [0.526,1.216] | 0.775 [0.478,1.257] | 0.943 [0.686,1.297] | 0.88 [0.691,1.119] |
| **Place of delivery** |  |  |  |  |  |
| Health facility-Pub | 1.000 | 1.000 | 1.000 | 1.000 | 1.000 |
| Health facility-Pri | 3.379 [0.936,12.198] | 0.742 [0.43,1.279] | 1.191 [0.744,1.906] | 1.242 [0.66,2.337] | 1.054 [0.772,1.441] |
| Home/Other | 0.781 [0.456,1.337] | 0.751 [0.489,1.154] | 1.464 [0.744,2.883] | 0.716 [0.488,1.051] | 0.841 [0.638,1.108] |
| **Visited health facility in last 12 months** |  |  |  |  |  |
| No | 1.000 | 1.000 | 1.000 | 1.000 | 1.000 |
| Yes | 0.799 [0.542,1.177] | 1 [0.69,1.449] | 1.214 [0.855,1.725] | 1.072 [0.824,1.396] | 0.957 [0.787,1.164] |
| **Father's education** |  |  |  |  |  |
| no education | 1.000 | 1.000 | 1.000 | 1.000 | 1.000 |
| primary | 0.431 [0.201,0.924] | 1.354 [0.747,2.454] | 1.326 [0.716,2.455] | 0.907 [0.631,1.304] | 0.86 [0.605,1.222] |
| secondary | 0.293 [0.13,0.66] | 1.544 [0.807,2.954] | 1.173 [0.61,2.255] | 1.052 [0.684,1.615] | 0.776 [0.534,1.129] |
| higher | 0.349 [0.078,1.555] | 1.099 [0.378,3.189] | 1.098 [0.446,2.701] | 1.736 [0.625,4.821] | 0.769 [0.437,1.352] |
| **Father's occupation** |  |  |  |  |  |
| Not working | 1.000 | 1.000 | 1.000 | 1.000 | 1.000 |
| white collar | 0.42 [0.048,3.623] | 0.988 [0.564,1.73] | 0.651 [0.137,3.087] | 0.271 [0.094,0.779] | 0.778 [0.354,1.706] |
| Agri worker | 0.696 [0.083,5.797] | 0.968 [0.605,1.547] | 0.872 [0.173,4.376] | 0.413 [0.153,1.11] | 0.978 [0.447,2.137] |
| service/manual work | 0.482 [0.058,4.006] | 1 [0,0] | 0.989 [0.202,4.837] | 0.383 [0.14,1.042] | 0.955 [0.442,2.067] |
| **Place of residence** |  |  |  |  |  |
| Urban | 1.000 | 1.000 | 1.000 | 1.000 | 1.000 |
| Rural | 1.079 [0.644,1.806] | 0.802 [0.475,1.352] | 0.665 [0.405,1.092] | 0.965 [0.67,1.39] | 0.93 [0.715,1.21] |
| **Wealth Index** |  |  |  |  |  |
| Poorest | 1.000 | 1.000 | 1.000 | 1.000 | 1.000 |
| Poorer | 0.929 [0.507,1.702] | 0.669 [0.363,1.232] | 0.786 [0.431,1.432] | 1.423 [0.981,2.064] | 0.858 [0.616,1.194] |
| Middle | 0.71 [0.379,1.33] | 0.715 [0.375,1.363] | 0.558 [0.295,1.056] | 1.327 [0.882,1.997] | 0.697 [0.496,0.979] |
| Richer | 0.546 [0.28,1.065] | 0.536 [0.272,1.053] | 0.56 [0.285,1.099] | 1.172 [0.726,1.892] | 0.625 [0.436,0.895] |
| Richest | 0.552 [0.242,1.255] | 0.62 [0.266,1.444] | 0.549 [0.247,1.216] | 1.023 [0.56,1.87] | 0.736 [0.476,1.137] |
| **Household Size** |  |  |  |  |  |
| <=4 | 1.000 | 1.000 | 1.000 | 1.000 | 1.000 |
| 5-9 | 1.263 [0.808,1.973] | 1.025 [0.68,1.544] | 0.965 [0.656,1.421] | 1.113 [0.812,1.525] | 1.015 [0.814,1.265] |
| >=10 | 0.996 [0.455,2.182] | 1.248 [0.561,2.776] | 0.932 [0.459,1.892] | 0.932 [0.545,1.593] | 1.068 [0.706,1.614] |
| **Sex of head of household** |  |  |  |  |  |
| Male | 1.000 | 1.000 | 1.000 | 1.000 | 1.000 |
| Female | 0.731 [0.434,1.23] | 1.518 [0.931,2.476] | 0.618 [0.429,0.89] | 1.054 [0.73,1.522] | 0.913 [0.721,1.156] |
| **Year of Survey** |  |  |  |  |  |
| 2005 |  |  |  |  | 1.000 |
| 2010 |  |  |  |  | 0.925 [0.691,1.238] |
| 2014 |  |  |  |  | 0.782 [0.565,1.082] |
| 2021 |  |  |  |  | 3.765 [2.352,6.027] |

a= Cambodia DHS survey year 2005, b= Cambodia DHS survey year 2010, c= Cambodia DHS survey year 2014, d= Cambodia DHS survey year 2021, e= Cambodia DHS survey year 2005-2021

Source: Authors estimation using Demographic and Health Survey (DHS) Data.
